# Fossil coleoid cephalopod from the Mississippian Bear Gulch Lagerstätte sheds light on early vampyropod evolution

**DOI:** 10.1038/s41467-022-28333-5

**Published:** 2022-03-08

**Authors:** Christopher D. Whalen, Neil H. Landman

**Affiliations:** 1 Department of Invertebrate Paleontology, American Museum of Natural History, New York, NY 10024 USA; 2 Department of Earth and Planetary Sciences, Yale University, New Haven, CT 06511 USA

**Keywords:** Palaeontology, Phylogenetics, Taxonomy, Bayesian inference, Marine biology

## Abstract

We describe an exceptionally well-preserved vampyropod, *Syllipsimopodi bideni* gen. et sp. nov., from the Carboniferous (Mississippian) Bear Gulch Lagerstätte of Montana, USA. The specimen possesses a gladius and ten robust arms bearing biserial rows of suckers; it is the only known vampyropod to retain the ancestral ten-arm condition. *Syllipsimopodi* is the oldest definitive vampyropod and crown coleoid, pushing back the fossil record of this group by ~81.9 million years, corroborating molecular clock estimates. Using a Bayesian tip-dated phylogeny of fossil neocoleoid cephalopods, we demonstrate that *Syllipsimopodi* is the earliest-diverging known vampyropod. This strongly challenges the common hypothesis that vampyropods descended from a Triassic phragmoteuthid belemnoid. As early as the Mississippian, vampyropods were evidently characterized by the loss of the chambered phragmocone and primordial rostrum—traits retained in belemnoids and many extant decabrachians. A pair of arms may have been elongated, which when combined with the long gladius and terminal fins, indicates that the morphology of the earliest vampyropods superficially resembled extant squids.

## Abstract (web summary)

The authors describe a new cephalopod from the Carboniferous (Mississippian) Bear Gulch Lagerstätte of Montana, USA. This specimen extends the fossil record of vampyropods back by ~82 million years and changes our understanding of their evolution.

## Introduction

**Fig. 1:**
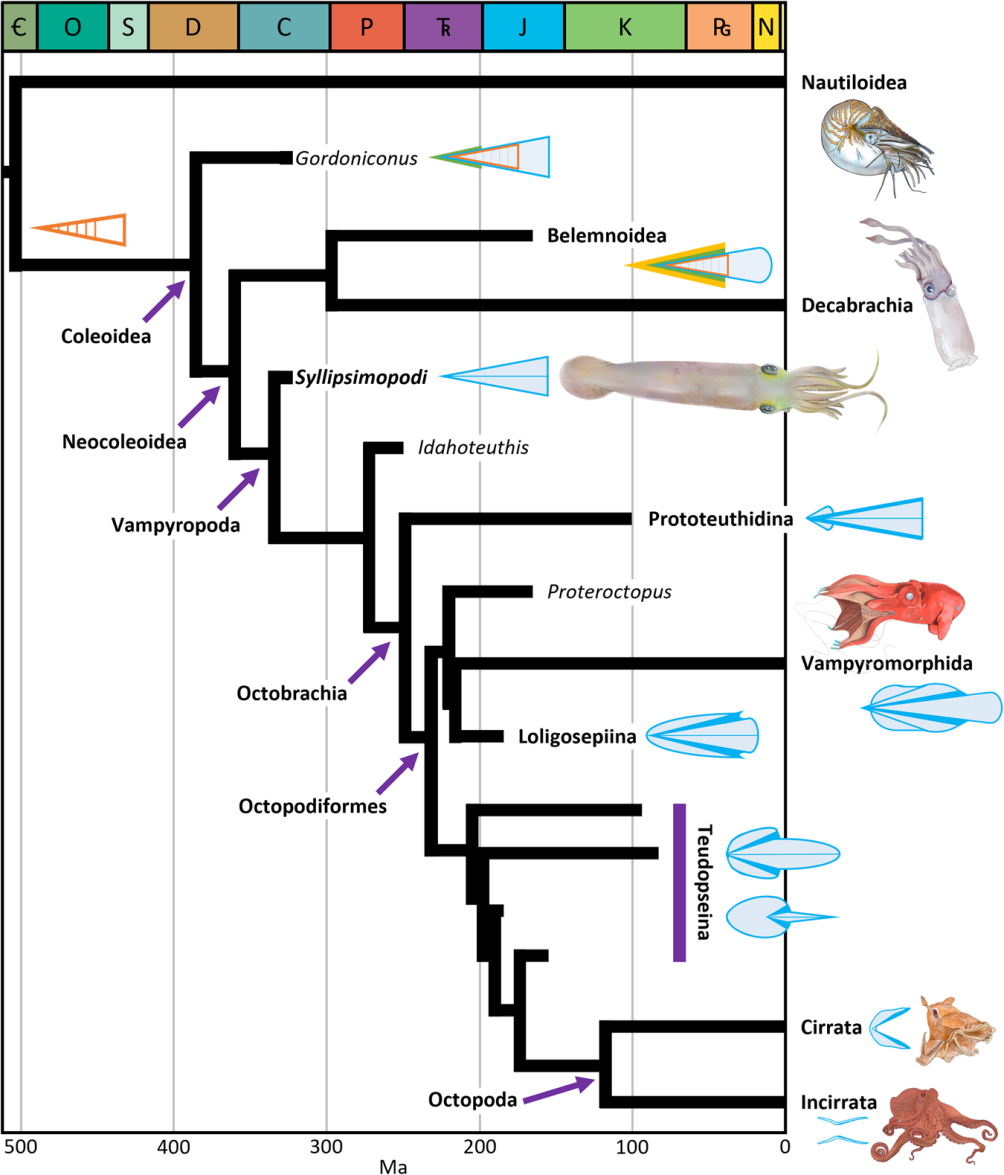
Overview of neocoleoid interrelationships and divergence time estimates, showing the position of S*yllipsimopodi bideni* gen. et sp. nov. Based on our Bayesian tip-dated phylogenetic reconstruction (Fig. 6). Shells color coded: blue = proostracum/gladius (hyperbolar zones and lateral reinforcements in darker blue), orange = phragmocone, green = primordial rostrum, yellow = rostrum. Geologic period abbreviations (colors from International Commission on Stratigraphy):  = Cambrian (dark green), O = Ordovician (teal), S = Silurian (light blue), D = Devonian (brown), C = Carboniferous (blue), P = Permian (red orange), T_R_ = Triassic (purple), J = Jurassic (cyan), K = Cretaceous (green), P_G_ = Paleogene (orange), N = Neogene (yellow), unlabeled = Quaternary (pale yellow). Purple arrows indicate named nodes, purple bar indicates teudopseid grade. Artistic depictions created by K. Whalen.

Vampyropoda, the clade combining octopods, vampyromorphs, and their relatives, is one of three main groups of coleoid (internally-shelled) cephalopods, the other two being Decabrachia (squids, cuttlefishes, bobtail squids, and *Spirula*) and the extinct Belemnoidea (Fig. 1). The heavily-mineralized belemnoids are the most common fossil coleoids, but they appear to lack living descendants^1,2^ and thus provide limited insight into the evolution of extant groups (although some have proposed that sepiids and/or spirulids are living belemnoids^3–5^). While less common, non-mineralized fossil vampyropods are known from several Mesozoic lagerstätten—their unique biochemistry appears to improve the preservation potential of their soft tissues^6^.

**Fig. 2:**
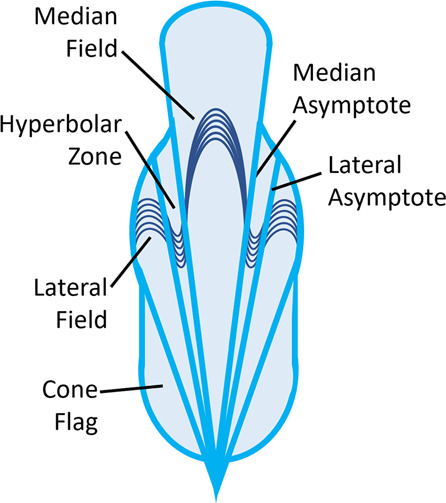
Idealized drawing of vampyropod gladius (based on *Vampyroteuthis*). Showing median field, hyperbolar zones, lateral fields, and cone flags, with examples of growth lines. Asymptotes denote borders of hyperbolar zones.

The gladius is a flattened shell-remnant found in vampyromorphs, extinct vampyropods, and many extant decabrachians; some extant octopods retain a vestigial gladius in the form of fin supports (cirrates) or stylets (incirrates)^2,7,14^. The gladius is typically composed of three parts (Fig. 2)—a central median field (or rachis) is laterally flanked by hyperbolar zones (or vanes) that are themselves flanked by lateral fields (or wings); there may additionally be cone flags lateral to the later fields (Fig. 2)^2,14^. Zones are often distinguished by growth line orientations: anteriorly convex median fields and anterolaterally convex lateral fields are separated by anteriorly concave hyperbolar zones^2^ (Fig. 2). Most of the gladius is dorsally situated, but the posterior often has a much shorter ventral and lateral component extending anterior from the apex in a cup or cone shape; this is the conus^2^.

The oldest known definitive vampyropod was considered to be a poorly preserved and fragmentary specimen of the prototeuthid *Germanoteuthis* from the Middle Triassic (Ladinian, ~241.5–237.0 Ma)^7,8^. However, molecular divergence time estimates suggest vampyropods diverged from decabrachians in the Carboniferous-Permian (289.7 ± 37.6 Ma)^9^. *Pohlsepia*^10^ was proposed as a Carboniferous cirrate octopod, but this is very controversial and the fossil seems unlikely to be a cephalopod or mollusc^7,9,11–13^.

**Fig. 3:**
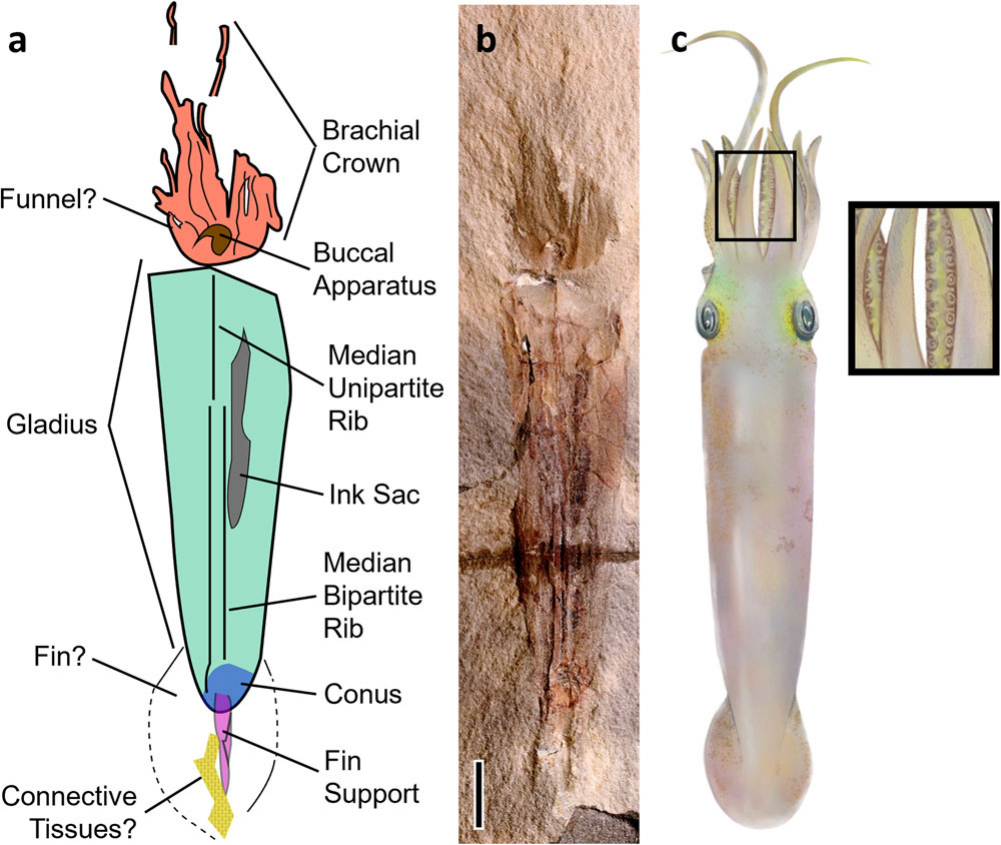
*Syllipsimopodi bideni* gen. et sp. nov., holotype ROMIP 64897. **a** Schematic drawing of *Syllipsimopodi bideni* gen. et sp. nov.; teal = gladius, orange = head (including arms), brown = buccal apparatus, gray = ink sac, blue = conus, magenta = fin support, patterned yellow = scale-like patches (possible connective tissue remnant). **b** Increased contrast false color image of *Syllipsimopodi*, holotype ROMIP 64897. Scale = 1 cm. **c** Artistic reconstruction showing suckers (created by K. Whalen).

Here we describe the oldest and earliest diverging vampyropod, *Syllipsimopodi bideni* gen. et sp. nov. (Figs. 2, 3), from the Mississippian (Serpukhovian, ~330.3–323.4 Ma)^8^ Bear Gulch Lagerstätte of Montana, USA. It possesses a gladius, fins, 10 arms, and suckers—evidence of a coleoid affinity. The fossil demonstrates that vampyropods originated in the Palaeozoic, corroborating molecular clock estimates^9^ and extending the stratigraphic range of known fossil vampyropods by ~81.9 million years. We substantially revise and expand the Sutton et al.^1,2^ neocoleoid morphological character-taxon matrix in order to place *Syllipsimopodi* into a Bayesian tip-dated phylogenetic framework using the Fossilized Birth-Death (FBD) model (Supplementary Data 1). All major neocoleoid groups are covered, with an emphasis on gladius-bearing fossil species.

## Results

Class: Cephalopoda Cuvier 1795^15^

Subclass: Coleoidea Bather 1888^16^

Clade: Vampyropoda von Boletzky 1992^17^

*Syllipsimopodi bideni* gen. et sp. nov.

**Etymology**. The genus name is derived from the Greek συλλήψιμος (syllípsimos) for prehensile and πόδι (pódi) for foot. The name prehensile-foot is chosen because this is the oldest known cephalopod to develop suckers, allowing the arms, which are modifications of the molluscan foot, to better grasp prey and other objects. The species name is to celebrate the recently inaugurated (at the time of submission) 46^th^ President of the United States, Joseph R. Biden.

**Holotype.** ROMIP 64897 (Royal Ontario Museum).

**Material.** The type and only specimen was donated to the Royal Ontario Museum by B. Hawes in 1988; accession number 88-72717. There is no counterpart.

**Locality.** Bear Gulch Limestone, Heath Formation, Big Snowy Group, Fergus County, Montana, USA^18^. The Bear Gulch Limestone is a plattenkalk, or lithographic limestone, similar to the more famous Jurassic Solnhofen Limestone of Germany^19^. Deposition occurred in a low-latitude shallow marine bay subject to oscillating semi-arid and tropical conditions^18^. Exceptional preservation is likely a result of  microturbidites deposited by seasonal monsoons^18^. Monsoonal rainfall would have rapidly introduced terrestrial sediments and biomatter into the bay, feeding algal blooms that created short-lived anoxic zones simultaneous with the saline instability caused by the rapid injection of voluminous freshwater^18^. Bear Gulch is perhaps best known for the pelagic fauna of the central basin and bay mouth – a diverse array of vertebrates^20–22^ (especially chondrichthyans^22–25^ and coelacanthiforms^22,26^), malacostracans^19,27^, polychaetes^22^, and cephalopods^13,28–30^, which are preserved in such exquisite detail that vascularization can sometimes be distinguished^31^. Benthic fossils are very rare in the central basin^18,22^, but marginal facies preserve gastropods, worms, asterozoans, and abundant sponges, which acted as a substrate for various brachiopods, bivalves, and conulariids^18,19,22^. Crinoids, blastoids, bryozoans, and corals are almost absent; algae (especially dasyclads) are common throughout^18,22^.

**Horizon**. Bear Gulch Limestone, Arnsbergian E2b (~328.3–324.5 Ma), Serpukhovian (Namurian), Mississippian, Carboniferous^8,18,32^.

**Fig. 4:**
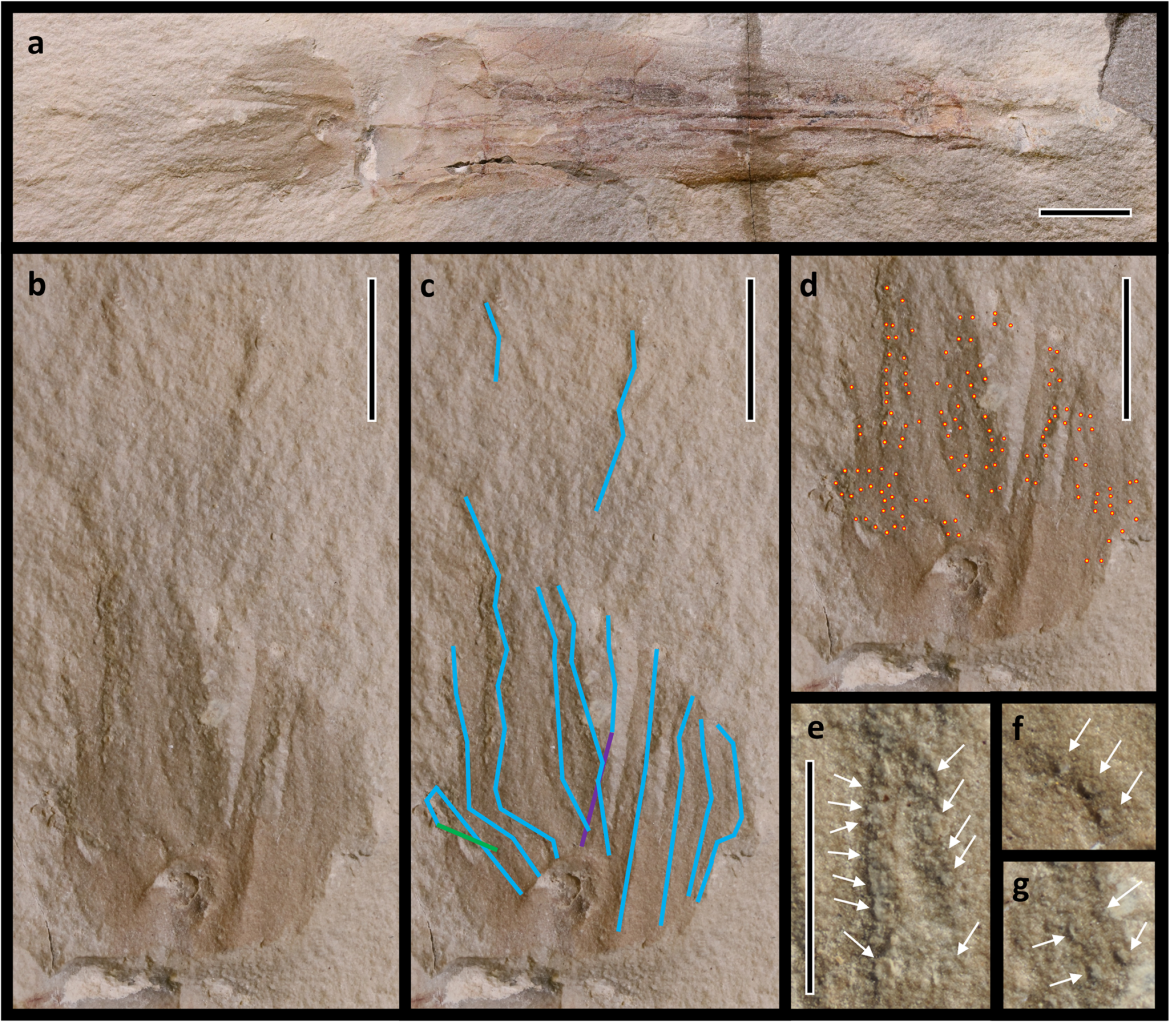
*Syllipsimopodi bideni* gen. et sp. nov., holotype ROMIP 64897, showing arm crown. **a**–**d** Scale = 1 cm. **a** Complete body fossil. **b**–**d** Showing arm crown; **c** arm traces in blue, purple indicates the arm is overlapping below two other arms, green indicates the arm is overlapping above itself; **d** red and yellow circles mark individual suckers. **e**–**g** scale = 5 mm; closeup of arms showing suckers, select suckers indicated with white arrows.

**Diagnosis.** (Figs. 3, 4) Coleoid with simple, nearly triangular gladius, bearing funnel-like conus and median field with median rib, but no hyperbolar zones, cone flags, or lateral reinforcements; lateral fields unlikely. Lacking chambered phragmocone, primordial rostrum, or rostrum. Ten arms bearing biserial rows of suckers but no hooks or cirri; two arms may be elongated (though this could be taphonomic). Ink sac present. Terminal median fin support and one fin pair present.

**Description**. The gladius median field is simple; it is widest at the extreme anterior with straight lateral edges and a flat (not rounded/pointed) anterior edge (Figs. 3a, 4). The median asymptote angle is ~13.8°. The gladius length to width ratio is 3.17; the median field is ~6.5 cm long, which is ~55% of the total body length. The median field bears a prominent median rib, which is diagenetically distorted and broken in various places (Supplementary Fig. 2). The rib appears to be posteriorly bipartite and anteriorly unipartite (Figs. 2, 3). Gladius growth lines are poorly preserved and only clearly visible in one place on the lateral edge (Supplementary Fig. 3). The funnel-like conus is ~6.8 mm long (Figs. 2, 3).

**Fig. 5:**
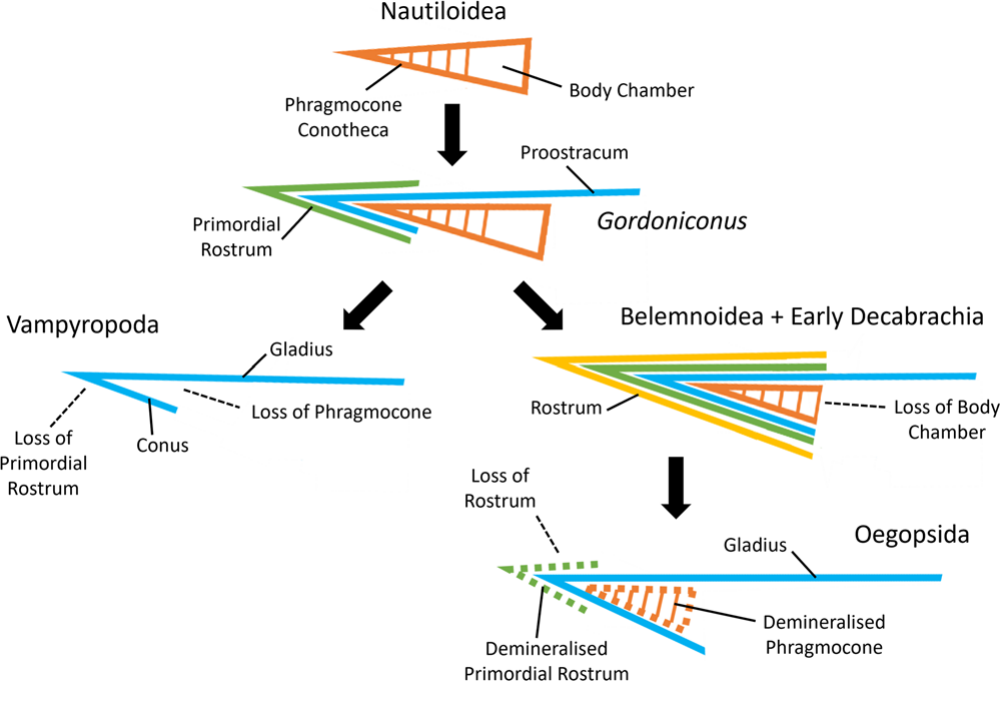
Overview of coleoid shell evolution, showing our interpretations of the gladius/proostracum. Early coleoids, such as *Gordoniconus*^13^, add the primordial rostrum^85^ and proostracum^14^; vampyropods lose the phragmocone and primordial rostrum, the proostracum is now a gladius^14^; belemnoids and early decabrachians lose the body chamber^86^ and add the rostrum^85^; oegopsids lose the rostrum, some retain a demineralized primordial rostrum^85^ and phragmocone^71^, the proostracum is now a gladius^14^. Structures only labeled when they appear (solid black line) or are lost (dashed black line). Shell tissues: orange = phragmocone + body chamber (dashed = demineralized), blue = proostracum/gladius, green = primordial rostrum (dashed = demineralized), yellow = rostrum.

A cigar-shaped central fin support measuring ~13.1 mm long is preserved posterior to the gladius (Fig. 3; Supplementary Fig. 4); an originally cartilaginous composition seems most likely. It is possible that this fin support is a vestige of the phragmocone, but we consider this alternative unlikely because there is no evidence of a siphuncle or septa. These are unlikely to have been dissolved without leaving a trace since septa can clearly be observed in the co-occurring coleoid *Gordoniconus*^13^. Septa are dissolved in Bear Gulch ammonoids^30^; but since *Gordoniconus* is a coleoid, we consider it a better taphonomic comparator. Also, the fin support is posterior to and thus external of the conus; the phragmocone should be internal to the conus (Fig. 5). We consider a primordial rostrum identity unlikely because it seems doubtful that a primordial rostrum (or rostrum) would be present in the absence of a phragmocone. The fin support is associated with patches of a shiny fibrous mineral (Fig. 3; Supplementary Fig. 4), presumed to be connective tissue remnants. The holotype appears to preserve a faint outline of a single pair of short terminal lobate fins measuring ~2.3 cm long anteroposteriorly and ~1.7 cm laterally across at the widest position (Figs. 3, 4).

Ten sucker-bearing arms are preserved, measuring ~2.1–2.4 mm wide at the midlength (Fig. 4b, c). Developmental evidence and phylogenetic inference have long suggested that the ten-arm condition is ancestral for cephalopods and vampyropods^33–35^, but no ten-armed fossils have been documented outside of the decabrachian-belemnoid clade prior to the discovery of *Syllipsimopodi*. *Syllipsimopodi* is the first and only known vampyropod to possess ten robust, functional appendages; all other known vampyropods have either reduced arm pair II to filaments (i.e., Prototeuthidina^7^, Loligosepiina^7,36^, Teudopseina^7^, Vampyromorphida), or lost the arm pair entirely (i.e., Octopoda).

Two arms, measuring ~4.0 and ~4.1 cm long (~27% of the total body length), might have been elongated relative to the other eight arms (Fig. 4b, c). These elongated arms do not have any obvious manus, as in decabrachian tentacles, and are not significantly thinner than the unmodified arms, as in vampyromorph filaments (Fig. 4b, c). Of the shorter arms, the three best preserved measure ~1.7, ~1.9, and ~1.9 cm long, respectively (~13% of the total body length); the remaining five arms are either incomplete or preserved in a contorted orientation (Fig. 4b, c). All arms appear to have suckers along the base and midlength (Fig. 4d). The better-preserved shorter arms appear to show distal suckers; it is unclear if the elongated arms bear suckers distally (Fig. 4d). Suckers are commonly between ~0.31 and ~0.62 mm in diameter. Suckers appear to have been biserial wherever present, but both rows are not always preserved along the entire arm length. Sucker rows are laterally separated by ~0.5 mm (Fig. 4e–g). Within a row, suckers are immediately adjacent proximodistally or separated by up to ~0.4 mm (Fig. 4e–g). There is no evidence that suckers were stalked.

Most arms are incompletely preserved, so it is possible that the apparent elongation of two of the arms is a taphonomic artifact. We consider this unlikely because exactly two arms are elongated and the elongated arms are of approximately similar lengths, suggesting they are from the same arm pair. Furthermore, the better-preserved shorter arms are each of approximately similar lengths. Probability suggests a preservational artifact would result in a non-two number of unequally elongated arms associated with shortened arms of dissimilar lengths. Additional fossil specimens will be necessary to test this hypothesis though. Based on the phylogenetic affinity of *Syllipsimopodi*, we interpret these as arm pair II. However, precise arm identities cannot be determined; this is an inference not an observation. *Syllipsimopodi bideni* is conservatively coded unknown in the phylogenetic analysis for all characters related to the modification/elimination of arm pair II (Supplementary Data 1).

A possible funnel measuring ~2.4 mm long is preserved at the lateral edge of the head (Fig. 3). A funnel identification is advocated because suckers appear to be absent on the structure (Fig. 4d), and we cannot easily connect it to one of the ten identified arms (Fig. 4b, c).

The buccal apparatus is preserved as a dark rectangular patch within a light circular patch that is distinct from the surrounding arm/head tissues (Fig. 3, Supplementary Fig. 5). The dark structure (~3.5 × ~1.3 mm) appears to be texturally distinct from the other preserved tissues, suggesting it could be a remnant of the beak. An intriguing S-shaped band measuring ~0.33 mm in width is preserved within the buccal apparatus. It is possible that this band is a remnant of the radula, but we suspect it is more likely to be a superimposed sucker-bearing arm because of the relatively long length (in comparison to the dark rectangular patch) and the apparent circular shapes lining parts of the band.

A dark, contiguous, anteroposteriorly elongate, saclike structure is preserved laterally offset from the central median ridge of the gladius. We interpret this as the ink sac. The ink sac measures ~2.6 cm long anteroposteriorly and ~0.3 cm wide laterally at the widest point (Figs. 3, 4).

**Ecological interpretations**. The preserved soft tissues and gladius suggest a torpedo-shaped body reminiscent of extant squids. The fins appear to have been large enough to potentially function as a viable supplement to jet swimming, but their apparent circular shape and terminal position would seem to suggest a stabilizing role may have been more important. If the arms are preserved to approximately their total lengths, then one arm pair was considerably longer than the other four pairs, which were shorter (arm-to-body length ratio) than in most extant octobrachians^37^. It seems likely that the elongated arms captured prey to be confined and manipulated by the shorter arms, analogous to extant decabrachians. The type does not preserve identifiable gut contents, so diet is unknown. While *Vampyroteuthis* remains the best living analog for understanding extinct vampyropods (because it is the most plesiomorphic extant vampyropod), these observations suggest *Syllipsimopodi* may have filled a niche more similar to extant inshore squids, i.e., a midlevel nektic predator. It is not inconceivable that *Syllipsimopodi* may have used its sucker-laden arms to pry small ammonoids out of their shells, or ventured more inshore to similarly extract small brachiopods, bivalves, and/or conulariids^18,22^; this is speculation though.

**Remarks**. There is considerable disagreement regarding the proper terminology for the group combining vampyromorphs, octopods, and their ancestors—Octobrachia, Octopodiformes, and Vampyropoda are the most common names. The *Treatise on Invertebrate Paleontology*^7^ recommends Octobrachia as a superorder for all coleoids that have either lost appendage pair II or modified it into filaments (i.e., Prototeuthidina, Loligosepiina, Teudopseina, Vampyromorphida, and Octopoda). We follow this recommendation (Fig. 1) and thus consider Octobrachia to be an apomorphy-defined taxon. We suggest that the name Octopodiformes be retained for the crown group (Fig. 1), since this appears to be the more popular term in the neontological literature. The name Vampyropoda, which is popular amongst paleontologists and has been formally ranked above Octobrachia^38^, should be retained for the total group (Fig. 1). This keeps the three most common terms accurate as typically used in the literature, and provides clade names necessary for greater nomenclatural precision, without inventing additional terms that would further confuse the discussion.

**Fig. 6:**
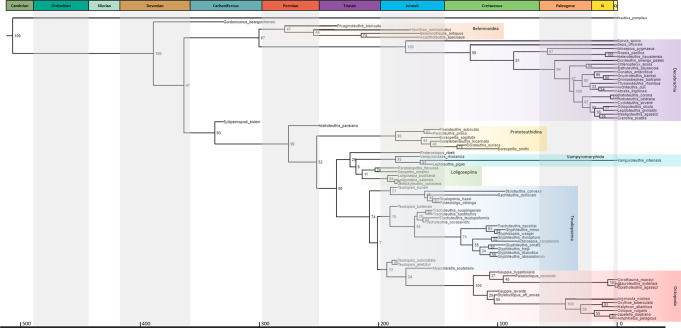
Bayesian tip-dated FBD (Fossilized Birth-Death) morphological phylogeny of neocoleoid cephalopods, showing the position of *Syllipsimopodi bideni* gen. et sp. nov. Numbers at nodes indicate posterior probabilities (percentage). Tips dated from the first appearance of the oldest member of the relevant lineage in the fossil record (see “Methods” and Supplementary Information). Showing geological timescale dated using Gradstein et al.^2^, dates in Ma and colors from International Commission on Stratigraphy; Q = Quaternary (pale yellow). Important taxa highlighted: orange = Belemnoidea, purple = Decabrachia, yellow = Prototeuthidina, green = Loligosepiina, cyan = Vampyromorphida, blue = ‘teudopseid’ grade, red = Octopoda. Tree does not show revised taxonomic designations: *Teudopsis bollensis* = *Briggsiteuthis bollensis* gen. et comb. nov., *Teudopsis jeletzkyi* = *Fuchsiteuthis jeletzkyi* gen. et comb. nov., *Teudopsis subcostata* = *Suttoniteuthis subcostata* gen. et comb. nov., *Glyphiteuthis rhinophora* = *Justinianiteuthis rhinophora* gen. et comb. nov., *Glyphiteuthis minor* = *Fisheriteuthis minor* gen. et comb. nov., and *Trachyteuthis bacchiai* = *Edmunditeuthis bacchiai* gen. et comb. nov. Tree drawn from MrBayes TRE output file using icytree.org. Source data are provided as a Source data file.

The Bayesian FBD (Fossilized Birth-Death) analysis reconstructs *Syllipsimopodi bideni* gen. et sp. nov. as the earliest-diverging vampyropod; the node is well supported with a posterior probability of 93% (Fig. 6). This placement is supported by the loss of the phragmocone, loss of the primordial rostrum, presence of a median ventral interruption on the gladius/proostracum, and dorsal shell (Supplementary Fig. 8, Supplementary Notes). The Early Triassic *Idahoteuthis*, which had been described as a possible myopsid squid^39^, is also recovered as an early vampyropod; the node has a posterior probability of 99% (Fig. 6). In addition to the characters shared with *Syllipsimopodi*, the position of *Idahoteuthis* is supported by the presence of lateral fields and the shape of the anterior median field (Supplementary Fig. 8, Supplementary Notes). Prototeuthidina is recovered as the earliest diverging octobrachian clade (Figs. 1, 6), unlike past parsimony analyses, which reconstructed the prototeuthids as derived loligosepiids^1,2^ or stem octopods^2^. These results better agree with recent non-cladistic arguments and the stratigraphic record – the oldest known octobrachians are the Triassic prototeuthids *Germanoteuthis* and *Reitneriteuthis*^7^. Rather than the basalmost octobrachian^1^, the Jurassic *Proteroctopus* is recovered as the basalmost stem vampyromorph (Figs. 1, 6). Loligosepiina is recovered as a clade sister to Vampyromorphida (Figs. 1, 6); previous cladograms reconstructed the loligosepiids as either a grade of stem octopodiforms^1^ or a grade of stem octopods^2^. *Vampyronassa* and *Leptoteuthis* (formerly a loligosepiid^7^ but never cladistically positioned within the group^1,2^) are recovered as vampyromorphs (Fig. 6).

Teudopseina is recovered as a paraphyletic grade of stem octopods (Figs. 1, 6). Past cladograms reconstructed them as total group vampyromorphs^2^, or as a combination of stem octopodiforms and stem octopods^1^; no phylogenies have recovered a monophyletic Teudopseina, Teudopseidae, or *Teudopsis*^1,2,40^. We establish new genera for *Teudopsis bollensis* (*Briggsiteuthis* gen. nov.), *Teudopsis jeletzkyi* (*Fuchsiteuthis* gen. nov.), and *Teudopsis subcostata* (*Suttoniteuthis* gen. nov.), which have each consistently been shown to be phylogenetically isolated from the type species, *Teudopsis bunelii*^1,2,38^ (Fig. 6). *T. jeletzkyi* and *T. subcostata* are not assigned the same genus because their clade (Fig. 6) is not found in other phylogenies^1,2^. Teudopseina could be maintained as a monophyletic rump group by restricting the suborder to Teudopseidae and Palaeololiginidae (Fig. 6)—this clade has been recovered in all phylogenies^1,2^. All analyses also suggest Teudopseidae is fully (no rump group) paraphyletic^1,2^ or polyphyletic (Fig. 6) with respect to Palaeololiginidae, which has nomenclatural seniority^7^, making Teudopseidae a junior synonym. The rump suborder Teudopseina would thus consist of one family, Paleololiginidae; we decline subordinal revision until interrelationships are more stable though. Unlike past studies^1,2^, we recover a monophyletic Trachyteuthidae (Fig. 6). We establish new genera for the trachyteuthids *Glyphiteuthis rhinophora* (*Justinianiteuthis* gen. nov.), *Glyphiteuthis minor* (*Fisheriteuthis* gen. nov.), and *Trachyteuthis bacchiai* (*Edmunditeuthis* gen. nov.) because each species has consistently been found isolated from its respective genus^1,2^ (Fig. 6). The latter two species were not reassigned to *Glyphidopsis* because that clade (Fig. 6) was not recovered in other phylogenies^1,2^. The Supplementary Discussion includes taxonomic details of the new genera.

We recovered belemnoid monophyly (Fig. 6), as in past analyses^1,2^; however, our results place Belemnoidea sister to Decabrachia (Fig. 1) rather than within Decabrachia, sister to Sepiida (cuttlefishes) and Sepiolida (bobtail squids)^1,2^. Our topology better concords with the stratigraphic record and molecular divergence time estimates. The oldest definitive fossil cuttlefish is the Maastrichtian *Ceratisepia*^41^ but the oldest definitive belemnoid is the Changhsingian phragmoteuthid *Permoteuthis*^42^. Both our morphological FBD tree (Fig. 6) and molecular clock estimates suggest cuttlefish split from *Spirula* in the Jurassic^43^. For belemnoids to be included within Decabrachia, the sepiid-spirulid split would need to occur in the Palaeozoic.

Phragmoteuthids have long been viewed as a likely precursor to the gladius-bearing coleoids, and hence vampyropods^44–46^, but this narrative has been rejected by all cladistic analyses^1,2^ (Fig. 6). Instead, phragmoteuthids consistently cluster with the other belemnoids and the belemnoids with decabrachians. Nevertheless, the idea has remained prominent in the literature^4,42^, in part because the tripartite phragmoteuthid proostracum seems reminiscent of vampyropod proostraca. This is explainable if lateral fields are a neocoleoid symplesiomorphy—meaning that either lateral fields were uniquely lost by *Syllipsimopodi*, or they were not well preserved in the *Syllipsimopodi* holotype (we could not conclusively determine presence/absence). Alternatively, the vampyropod lateral fields may have developed independently of comparable structures in belemnoids and decabrachians (Fig. 6). Past cladistic analyses^1,2^ did not test the stratigraphic arguments for phragmoteuthid origins. Phragmoteuthids originated in the latest Permian (Changhsingian)^42^, and the oldest vampyropods had previously been known from no earlier than the Middle Triassic (Ladinian)^7^; a comfortable timeline for divergence. However, our FBD phylogeny explicitly incorporates stratigraphy and still rejects a phragmoteuthid origin for Vampyropoda. This is unsurprising as *Syllipsimopodi* predates all known phragmoteuthids by nearly 70 million years, and it already possessed a dorsal gladius without a phragmocone or primordial rostrum.

Spirulida is here recovered as the basalmost decabrachian order (Fig. 6), but this may be biased by their plesiomorphic shell-condition. Nevertheless, results appear substantially more plausible than previous fossil-inclusive cladograms, which reconstructed spirulids as deeply nested within Decabrachia^1,2^. Molecular phylogenies have produced conflicting results for the position of *Spirula*, suggesting placement within Bathyteuthida^47^ or as sister to either Myopsida^48^, Oegopsida^9,49^, Bathyteuthida + Oegopsida^50^, or Sepiida^51^. We recover Sepiida as the next earliest diverging order, and the teuthids (Myopsida, Bathyteuthida, and Oegopsida) are recovered in a clade sister to Idiosepiida + Sepiolida (Fig. 6). Decabrachians were not a focus of this analysis though, and our limited sampling is not intended or expected to meaningfully resolve interrelationships within this group.

## Discussion

The phylogenetic analysis placed the origin of Neocoleoidea between 397.8 and 334.8 Ma, with a median of 363.4 Ma. This approximately coincides with the Frasnian-Famennian (371.1 Ma) and Devonian-Carboniferous boundaries (359.3 Ma)^8^: a dynamic interval noteworthy for the Late Devonian (Frasnian-Famennian) mass extinction, the initiation of the Late Palaeozoic Ice Age, and the end-Devonian mass extinction^8,52,53^. Collectively, these events fundamentally transformed the marine biosphere.

The mid-Palaeozoic greenhouse^54^ fostered the first forests^55^, one of the most extensive reef systems in Earth’s history^56^, the rise of vertebrates (i.e., fishes) as a major component of marine ecosystems^57–60^, and the origin of ammonoids^57–59,61,62^. Although conclusive fossil evidence is yet lacking, it stands to reason that this environment also cultivated the first coleoids; the divergence between *Gordoniconus* and Neocoleoidea is predicted to 431.6–355.5 Ma, with a median of 389.0 Ma (Middle Devonian, Eifelian^8^). The Big Five end-Frasnian event would mark the demise of the mid-Palaeozoic tabulate coral and stromatoporoid reefs (similarly extensive coral populations would not develop for ~200 million years)^56^ and was catastrophic for global biodiversity^63^, which continued to decline throughout the Fammenian^64^. However, except for extinctions among the remaining jawless ‘ostracoderm’ vertebrates^57^, swimming taxa appear to have been relatively unaffected^57,62,65^. In the immediate aftermath of the crisis, ammonoid disparity relative to diversity fell to its lowest Palaeozoic levels, but by the middle Famennian the same disparity metric reached its highest Palaeozoic value^62^. The end-Frasnian could have cleared ecospace for the nascent neocoleoids, and they may have benefited from the same conditions that fostered high ammonoid disparity in the Famennian.

The onset of the Late Palaeozoic Ice Age^52^, the first major glaciation since the end-Ordovician^54^, coincides with the end-Devonian mass extinction^53^. This event seems to have had less of a direct impact on benthic communities^56,64^, but it precipitated a major reorganization of the water column^57,65^. ‘Placoderm’ vertebrates, clymeniid ammonoids, and homoctenid tentaculitoids went extinct; the diversity of discosorid and oncocerid nautiloids as well as ‘acanthodian’ and sarcopterygian vertebrates was greatly diminished^57,65^. These negatively impacted groups are generally characterized as more heavily armoured, less hydrodynamic, and slower; in contrast the taxa that proliferated in the Mississippian oceans (e.g., euchondrichthyan and actinopterygian vertebrates, prolecanitid ammonoids) were generally less encumbered and more agile^57,65^. Our predicted origin of Neocoleoidea would neatly fit this pattern of marine reorganization and nektonization.

The Bear Gulch lagerstätte preserves a slice of the Carboniferous in the aftermath of another crisis, the mid-Serpukhovian mass extinction^53,57^. This event may be best known for concluding the Mississippian age of crinoids, but pelagic taxa were also impacted. Ammonoids, conodonts, and chondrichthyans in particular faced heightened extinction rates^53^. Ammonoid disparity precipitously contracted relative to diversity, and would not recover until the Moscovian^66^. Perhaps vampyropod origins were linked to these vacated niches; however, the divergence of *Syllipsimopodi* is estimated to the Tournaisian/Visean (354.4–330.3 Ma, median 337.5 Ma), making this scenario seem unlikely. Less controversially it could be argued that the traits exhibited by *Syllipsimopodi* contributed to the survival of vampyropods across several mass extinctions (mid-Serpukhovian, Guadalupian, end-Permian, and Late Triassic), through to their proliferation in the Jurassic^7^.

By internalizing the phragmocone, coleoids could add fins to supplement energetically expensive jet swimming. Furthermore, coleoids escaped the inherent restrictions imposed by the body chamber on the maximum size of the head, buccal apparatus, funnel, and appendages, and the novel proostracum provides a large, lightweight plate to anchor this expanded anterior. Expansion of the anterior increases the anterior weight, but the posterior phragmocone remains filled with buoyant gas, so the animal could be forced into a downward vertical orientation rather than the presumably preferable horizontal (although observations of in situ *Spirula* may challenge this conventional wisdom^67^). The earliest coleoids, such as *Gordoniconus*, appear to solve this problem by encapsulating the phragmocone posteriorly in a mineralized primordial rostrum that can function as a counterweight^13^ (with the added benefit of increased gliding momentum after each jet pulse). Belemnoids elaborated on this with heavier rostra and epirostra, and extinct decabrachians, such as *Belopterina*^68^ and *Anomalosaepia*^69^, encapsulated their phragmocones in elaborate, mineralized sheaths. Vampyropods, however, took a different approach—neutral buoyancy was maintained through the ionic composition and seawater content of their tissues^6^, rather than the gaseous content of a posterior phragmocone. Thus, preventing the orientation problem entirely. Vampyropods appear to have then faced selective pressure to eliminate the non-functional phragmocone despite potential benefits to glide momentum. This would have decreased metabolic expenses, reduced weight, and freed additional space for soft tissues beneath the posterior portion of the shell’s proostracal layer, which is now a gladius.

The gladius is considered a derivative of the proostracum, an anterodorsal projection of the internal coleoid shell^14^. A widely accepted hypothesis holds that the proostracum is a remnant of the dorsal surface of the shell’s body chamber^14^. However, it has been demonstrated that the belemnoid proostracum is a lamello-organic layer extending to and encapsulating the phragmocone apex; this layer is situated between the mineralized inner conotheca and mineralized outer primordial rostrum^70^. Therefore, (1) the proostracum was likely never mineralized and always of a composition similar to derived gladii, (2) the proostracum is evidently not composed of conothecal tissues (i.e., outer prismatic layer, nacreous layer, inner prismatic layer) and so cannot be formed through the partial loss of the conotheca, and (3) the proostracum is not restricted to the body chamber so it is unlikely to be a remnant of it. This reassessment of proostracal homology also implies that the conus is not homologous with or a remnant of the phragmocone. Instead, the conus is the portion of the shell apex composed of proostracal tissues; the conus would overlay the phragmocone apex if a phragmocone was present (Fig. 5). This would explain the absence of a siphuncle and septa in the vampyropod conus—vampyropods have completely lost a phragmocone and only retain the overlying proostracal layer. This covering tissue never lined the inner siphuncle or septa, so no remnant of these structures would be expected. In contrast, many decabrachians retain an unmineralized phragmocone as a distinct layer within the conus (and below the gladius), complete with siphuncle and septa^71^. So, it seems unlikely that the proostracum is a vestige of the body chamber. Instead, the proostracum appears more likely to be an anterior extension of the proostracal layer, which ancestrally encapsulated the coleoid shell. This proostracal layer may be homologous with the nautilid periostracum^14^.

*Syllipsimopodi* is the oldest known vampyropod and gladius bearing cephalopod by ~81.9 million years^7,8^, as such it provides unparalleled insight into the origins of the vampyropod bauplan. The median field of *Syllipsimopodi* resembles an acute triangle opening anteriorly from the apex and terminating in a flat anterior edge; no hyperbolar zones are present and lateral fields are either absent or small. The conus is small and posteriorly restricted. This simple gladius recalls the proostracum of the stem coleoid *Gordoniconus*, which appears to be a simple anterior continuation of the shell passed the phragmocone. The gladius of *Syllipsimopodi* also resembles prototeuthids, except the lateral edges of the prototeuthid median field are reinforced (a synapomorphy of the suborder), and prototeuthids have unambiguous (albeit simple) lateral fields near the shell apex. Loligosepiids and vampyromorphs add a simple curved anterior edge to the median field, more elongated lateral fields, and prominent hyperbolar zones; among these taxa, the funnel-shaped conus reminiscent of a phragmocone apex is also replaced by the cup-shaped conus. Teudopseids further elaborate on this structure with a distinctly pointed anterior edge of the median field, and more complex lateral fields and hyperbolar zones.

An evolutionary trajectory can thus be observed among vampyropods. (1) Stem coleoids (*Gordoniconus*) elongated the proostracal-layer anterior to the body chamber, forming the proostracum. (2) By the Late Mississippian, vampyropods (*Syllipsimopodi*) formed a gladius through elimination of the mineralized underlying phragmocone and overlying primordial rostrum. Two appendages (arm pair II) may have been elongated, converging on the tentacles of later decabrachians (arm pair IV), but without doubt all ten arms remained robust. (3) By the Early Triassic, noticeable lateral fields had been added to the gladius (*Idahoteuthis*). (4) Arm pair II, which may have already been developmentally isolated from the other arm pairs, was reduced to a vestigial filament in octobrachians. (5) Octopodiforms expanded the hyperbolar zone between the median and lateral fields; the conus took on a cup shape. Muensterelloid teudopseids fully opened the conus, forming a patella. (6) Octopods eliminated the median field, forming either fin supports (cirrates) or stylets (incirrates); arm pair II was lost.

Excluding octopods, the pattern of vampyropod evolution after origination seems to be one of addition not subtraction. Structures are added to the proostracum that are then expanded and elaborated via increasingly complex developmental controls; there is no evidence for a gradual evolutionary reduction of extensive phragmoteuthid-like lateral fields or of an anteriorly elongate conus. If the gladius is a remnant of the body chamber, then the ventrolateral reductions must have occurred rapidly. Alternatively, the vampyropod gladius formed via a simple mutation that turned off the developmental processes responsible for the formation of the biomineralized shell layers. The remaining structure would have been a simple dorsal plate that posteriorly wrapped around the apex (i.e., the proostracum); no complex sequence of gradual reductions is necessary. We suggest this latter scenario is a much more plausible model for the formation of the gladius. Even if evidence was equivocal, the scenario we propose requires fewer genetic alterations (1 change = loss of all mineralized tissues) than the conventional model (7 changes = loss of ventral body chamber, loss of lateral body chamber, loss of primordial rostrum, loss of septa, loss of siphuncle, shortening of conus, demineralization). However, evidence is not equivocal: no phylogeny has ever suggested vampyropods derived from a phragmoteuthid-like ancestor. Furthermore, *Syllipsimopodi* predates all known neocoleoids and already has a fully developed gladius without a phragmocone. *Syllipsimopodi* and our phylogeny demonstrate that vampyropods likely originated soon after the origin of neocoleoids. The rapid formation of the gladius can be more easily explained through the modification of one fundamental developmental process, than by the gradual loss of a series of complex structures.

*Syllipsimopodi bideni* gen. et sp. nov. is a Carboniferous gladius-bearing vampyropod with ten robust, sucker-bearing arms. It is the oldest known vampyropod and neocoleoid. Neocoleoids originated before the Serpukhovian; likely in the Late Devonian, potentially as a component of the broader pelagic reorganizations of the Famennian to Tournaisian. Our Bayesian FBD phylogeny reconstructs *Syllipsimopodi* as the basalmost vampyropod, *Idahoteuthis* as a vampyropod, Prototeuthidina as the basalmost octobrachian clade, *Proteroctopus* as the basalmost stem vampyromorph, Loligosepiina as a clade of stem vampyromorphs, Teudopseina as a paraphyletic grade of stem octopods, *Phragmoteuthis* as a belemnoid, and a monophyletic Belemnoidea as sister to Decabrachia. By the Serpukhovian, vampyropods already possessed a fully-formed gladius without a phragmocone. This, together with the reaffirmed rejection of the phragmoteuthid-origins hypothesis, suggests the proostracum/gladius is unlikely to be a remnant of the phragmocone body chamber.

The conventional model for the formation of the gladius could be plausible if vampyropods originated long after the Devonian origins of Neocoleoidea, if we had a sequence of fossils showing gradual reductions of the body chamber and phragmocone, if the earliest proostraca were mineralized, if vampyropods exhibited a general trend towards reduction of the gladius, or if vampyropods descended from phragmoteuthids. However, each of these lacks or is contradicted by evidence. *Syllipsimopodi* demonstrates that vampyropods existed in the Mississippian and already possessed a demineralized gladius without any discernible remnant of the phragmocone. *Syllipsimopodi* shows that the earliest gladii were simple structures that were elaborated on by succeeding lineages; not complex shells to be evolutionarily reduced to an essential minimum. Lastly, no fossils have ever been discovered that might indicate a gradual reduction of the body chamber except for phragmoteuthids, and our phylogenetic analysis reaffirms the growing consensus that phragmoteuthids are not stem vampyropods.

## Methods

This study complies with all relevant ethical regulations; no approval was necessary from any oversite board/committee.

### Phylogenetic analysis

We redesigned the morphological character-taxon matrix of Sutton et al.^2^ (expanded by Kruta et al.^1^) under a more extensive and explicit contingency coding framework; adding characters and deleting overlapping characters as necessary. All characters are detailed in the Supplementary Methods. We added *Syllipsimopodi bideni* (ROMIP 64897), *Gordoniconus beargulchensis*^13^ (AMNH 43264, 50267), *Idahoteuthis parisiana*^39^, and *Acanthoteuthis speciosus*^72^. We removed the Late Cretaceous teudopseids *Marekites vinarensis* and *Eoteuthoides caudata* because both are only preserved as a fragmentary conus, providing too few phylogenetically informative characters^7^. We also removed the Carboniferous species *Jeletzkya douglassae* and *Pohlsepia mazonensis*. All included taxa are detailed in the Supplementary Methods.

*Jeletzkya* was removed because almost no characters could be confidently coded. It has only been described on the basis of a radiograph showing a faint, vaguely spatulate structure and attached hook-bearing appendages^73^. The fossil was not prepped out of its concretion^73^ or micro-CT scanned, and it has never yet been clearly observed or redescribed. The spatulate structure was interpreted as a gladius, but this was not based on any clearly diagnostic characters and an alternative identification as a phragmocone, rostrum, or some other structure cannot be ruled out. *Jeletzkya* is described as having ten arms, but the authors did not indicate where each arm is in the radiograph; we can only distinguish 4 vaguely rectangular patches barely distinct from static^73^. Thus, the only characters that could reliably be coded would be ‘presence of a shell’ and ‘presence of arm hooks’. The hooks could be diagnostic for belemnoids, but the fossil is far too poorly understood for assignment to any particular cephalopod taxon, let alone inclusion in a phylogenetic analysis. Exclusion of *Jeletzkya* has minimal impact on this investigation as belemnoids are not the primary focus.

The Mazon Creek fossil *Pohlsepia* was initially described as a cirrate octopod^10^, however, it is unlikely to be a cirrate, vampyropod, cephalopod, or mollusc. *Pohlsepia* vastly predates the oldest known octopods (Late Cretaceous)^7^, molecular divergence time estimates for the cirrate–incirrate split (Jurassic)^9^, and all other seemingly-ancestral octobrachians except for *Syllipsimopodi*. *Pohlsepia* lacks a gladius (or gladius vestige), phragmocone, or primordial rostrum^10^—a highly unlikely combination in a Carboniferous cephalopod. The proposed appendages^10^ lack hooks, suckers, cirri, an arm web, and the characteristic 8/10 arm count; there is neither a beak, unambiguous ink sac, nor radula. The suggested fins^10^ could alternatively be interpreted as taphonomically deformed folds of tissue. The only confidently known characters, the bulbous body outline and presence of appendages, are not cephalopod diagnostic; a cnidarian affinity may be more likely. Because it lacks nearly all coleoid and cephalopod synapomorphies, past phylogenies recovered it at the base of the tree^1,2^. This effectively creates a second, misleading, outgroup that biases character polarity throughout the phylogeny. Until a redescription of *Pohlsepia* can confidently determine its affinity, the genus should not be included in any studies of cephalopod interrelationships.

The final matrix consists of 79 taxa and 153 morphological characters. Tip dates refer to the first appearance (FAD) of each genus, binned to age and dated using Gradstein et al.^8^, with two exceptions. (1) The species FADs were used if multiple species from the same genus were encoded in the analysis. (2) The order/family FAD was used for each order/family with only one included species, i.e. Phragmoteuthida (FAD from *Permoteuthis*)^42^, Belemnitida (FAD from *Sichuanobelus*)^74^, Spirulida (FAD from *Kostromateuthis*)^75^, Sepiida (FAD from *Ceratisepia*)^41^, and Argonautidae (FAD from *Obinautilus*)^7^.

The outgroup, *Nautilus*, was dated to the Darriwilian (Middle Ordovician) based on *Centrocyrtoceras*^76^. It has been argued on the basis of molecular clock estimates that nautilids diverged from coleoids near the Silurian-Devonian boundary^11^; however, no cephalopod fossils were used to calibrate this clock, and the margin of error for the nautilid-coleoid split is ±60 million years^11^. The oldest nautilids are the Silurian (Wenlock) lechritrochoceratids^76,77^. The lechritrochoceratids are thought to descend from the Middle Ordovician uranoceratid tarphycerid *Centrocyrtoceras*^76^. This narrative, that tarphycerids are stem nautilids^76^, had largely been supplanted by the argument that nautilids originated from an unknown orthoceratoid ancestor^11,33^. However, the tarphycerid hypothesis has recently been revived and expanded as part of a larger effort to reclassify the various orders of fossil nautiloids^78^. In this revived scenario, nautilids descend from tarphycerids, tarphycerids descend from oncocerids, and oncocerids descend from ellesmerocerids^78^—a modernization of several much older proposals for nautiloid interrelationships^79–82^. No hypothesis of nautilid origins has yet been tested phylogenetically. We prefer the tarphycerid hypothesis because it outlines a specific testable fossil sequence^76^. In contrast, no orthoceratoid fossils have ever been proposed as possible stem nautilids. We conservatively use the FAD of *Centrocyrtoceras* (as opposed to the FAD of Ellesmerocerida) to date *Nautilus* in the FBD analysis, because no specific sequence has been proposed linking tarphycerids to oncocerids^76,78^. *Centrocyrtoceras* remains within the molecular clock margin of error for the nautilid-coleoid split^11,76^.

The character-taxon matrix was initially composed as a NEXUS file in Mesquite 3.61. The tip-dated Bayesian analysis was performed in MrBayes 3.2.7 under the fossilized birth-death (FBD) process. We used the independent gamma rates (IGR) clock model and a normally distributed clock rate prior with a mean of 0.0025 and a standard deviation of 0.1 changes per million years. The tree age was set to a minimum of 486.9 Ma—the base of the Ordovician^8^ and first appearance of *Bactroceras*^83^, which may be the oldest known stem coleoid^78^. The tree mean was set to 529.0 Ma, the base of Cambrian Stage 2^8^, because the upper Terreneuvian marks the oldest known fossil cephalopod^84^. The MCMC analysis ran 40,000,000 simulations of 4 chains with a burn in of 25,000 generations.

### Nomenclatural acts

This published work and the nomenclatural acts it contains have been registered in ZooBank, the proposed online registration system for the International Code of Zoological Nomenclature (ICZN). The ZooBank LSIDs (Life Science Identifiers) can be resolved and the associated information viewed through any standard web browser by appending the LSID to the prefix ‘http://zoobank.org/’. The LSID for this publication is: urn:lsid:zoobank.org:pub:57F16B89-1162-4261-922E-AD3B7FB54765.

### Photography

Photographs for Fig. 3b and Fig. 4a–d were taken using a Canon EOS 60D camera with an EF-S60mm f/2.8 Macro USM lens and a Hoya 52 mm Circular Polarizing Pro 1 digital multi-coated glass filter; Cognisys Stackshot 3X Macro Rail Package and Helicon Focus 6.7.1 Pro were used to z-stack images. Photographs for Fig. 4e–g and Supplementary Figs. 4–8 were taken using a Nikon D300 camera. Composite images were stitched using Adobe Photoshop 2021.

### Reporting summary

Further information on research design is available in the Nature Research Reporting Summary linked to this article.

### Supplementary information

Supplementary Information

Description of Additional Supplementary Files

Supplementary Data 1

Reporting Summary

### Source data

Source Data

## Data Availability

All data generated or analysed during this study are included in this published article (and its Supplementary Information files). The NEXUS data file used in this study is available in the MorphoBank database under accession code http://morphobank.org/permalink/?P4160. The morphological character-taxon matrix and tip dates from the NEXUS file (Supplementary Data 1) are also provided in the Supplementary Methods. The Source data file is the MrBayes TRE output. Correspondence and requests for materials should be addressed to Christopher D. Whalen at cwhalen@amnh.org. The type and only specimen of *Syllipsimopodi bideni* gen. et sp. nov. is reposited at the Royal Ontario Museum (ROMIP 64897). Museum specimen identification numbers for all other directly analysed fossils are in the Supplementary Methods. No ethical approval or guidance was required because this study only analysed invertebrate fossils in museum collections; no new material was collected and this study does not include any archaeological remains or Recent specimens. Source data are provided with this paper.

## References

[1] Kruta I (2016). *Proteroctopus ribeti* in coleoid evolution. Palaeontology.

[2] Sutton M, Perales-Raya C, Gilbert I (2016). A phylogeny of fossil and living neocoleoid cephalopods. Cladistics.

[3] Hewitt RA, Jagt JWM (1999). Maastrichtian *Ceratisepia* and Mesozoic cuttlebone homeomorphs. Acta Palaeontol. Pol..

[4] Fuchs D (2019). Homology problems in cephalopod morphology: deceptive (dis)similarities between different types of ‘caecum’. Swiss J. Palaeontol..

[5] Fuchs D, Keupp H, Wiese F (2012). Protoconch morphology of *Conoteuthis* (Diplobelida, Coleoidea) and its implications on the presumed origin of the Sepiida. Cretac. Res..

[6] Clements T, Colleary C, De Baets K, Vinther J (2017). Bouyancy mechanisms limit preservation of coleoid cephalopod soft tissues in Mesozoic lagerstätten. Palaeontology.

[7] Fuchs D, Part M (2020). Chapter 23G: systematic descriptions: Octobrachia. Treatise Online.

[8] Gradstein, F. M. & Ogg, J. G. in *Geologic Time Scale**2020* (eds. Gradstein, F. M., Ogg, J. G., Schmitz, M. D. & Ogg, G. M.) 21–32 (Elsevier, 2020).

[9] Tanner AR (2017). Molecular clocks indicate turnover and diversification of modern coleoid cephalopods during the Mesozoic Marine Revolution. Proc. R. Soc. B.

[10] Kluessendorf J, Doyle P (2000). *Pohlsepia mazonensis*, an early ‘octopus’ from the Carboniferous of Illinois, USA. Palaeontology.

[11] Kröger B, Vinther J, Fuchs D (2011). Cephalopod origin and evolution: a congruent picture emerging from fossils, development and molecules. Bioessays.

[12] Fuchs D (2009). Octobrachia - a diphyletic taxon?. Berl. Paläobiologische Abhandlungen.

[13] Klug C (2019). Anatomy and evolution of the first Coleoidea in the Carboniferous. Commun. Biol..

[14] Fuchs D, Iba Y (2015). The gladiuses in coleoid cephalopods: homology, parallelism, or convergence?. Swiss J. Palaeontol..

[15] Cuvier G (1795). Second Mémoire sur l’organisation et les rapports des animaux à sang blanc, dans lequel on traite de la structure des Mollusques et de leur division en ordre, lu à la société d’Histoire Naturelle de Paris, le 11 prairial an troisième. ou J. des. Sci. des. Lett. des. Arts.

[16] Bather FA (1888). Shell-growth in Cephalopoda (Siphonopoda). J. Nat. Hist..

[17] von Boletzky S (1992). Evolutionary aspects of development, life style, and reproductive mode in incirrate octopods (Mollusca, Cephalopoda). Rev. suisse Zool..

[18] Grogan ED, Lund R (2002). The geological and biological environment of the Bear Gulch Limestone (Mississippian of Montana, USA) and a model for its deposition. Geodiversitas.

[19] Williams LA (1983). Deposition of the Bear Gulch Limestone: a Carboniferous plattenkalk from central Montana. Sedimentology.

[20] Lund R, Janvier P (1986). A second lamprey from the Lower Carboniferous (Namurian) of Bear Gulch, Montana (U.S.A.). Geobios.

[21] Mickle KE, Lund R, Grogan ED (2009). Three new palaeoniscoid fishes from the Bear Gulch Limestone (Serpukhovian, Mississippian) of Montana (USA) and the relationships of lower actinopterygians. Geodiversitas.

[22] Lund R, Greenfest-Allen E, Grogan ED (2012). Habitat and diversity of the Bear Gulch fish: life in a 318 million year old marine Mississippian bay. Palaeogeogr. Palaeoclimatol. Palaeoecol..

[23] Grogan ED, Lund R, Fath M (2014). A new petalodont chondrichthyan from the Bear Gulch Limestone of Montana, USA, with reassessment of *Netsepoye hawesi* and comments on the morphology of holomorphic petalodonts. Paleontol. J..

[24] Lund R (1989). New petalodonts (Chondrichthyes) from the Upper Mississippian Bear Gulch Limestone (Namurian E2b) of Montana. J. Vertebr. Paleontol..

[25] Lund R (1985). The morphology of *Falcatus falcatus* (St. John and Worthen), a Mississippian stethacanthid chondrichthyan from the Bear Gulch Limestone of Montana. J. Vertebr. Paleontol..

[26] Lund R, Lund W (1984). New genera and species of coelacanths from the Bear Gulch Limestone (Lower Carboniferous) of Montana (U.S.A.). Geobios.

[27] Schram FR, Horner J (1978). Crustacea of the Mississippian Bear Gulch Limestone of central Montana. J. Paleontol..

[28] Landman NH, Davis RA (1988). Jaw and crop preserved in an orthoconic nautiloid cephalopod from the Bear Gulch Limestone (Mississippian, Montana). N. Mex. Bur. Mines Miner. Resour..

[29] Mapes, R. H., Weller, E. A. & Doguzhaeva, L. A. in *Cephalopods - Present and Past* (Tanabe, K., Shigeta, Y., Sasaki, T. & Hirano, H. eds.) 155–170 (Tokai University Press, 2010).

[30] Landman, N. H., Mapes, R. H. & Cruz, C. in *Cephalopods—Present and Past* (eds. Tanabe, K., Shigeta, Y., Sasaki, T. & Hirano, H.) 147–153 (Tokai University Press, 2010).

[31] Grogan ED, Lund R (2000). *Debeerius ellefseni* (fam. nov., gen. nov., spec. nov.), an autodiastylic chondrichthyan from the Mississippian Bear Gulch limestone of Montana (USA), the relationships of the chondrichthyes, and comments on gnathostome evolution. J. Morphol..

[32] Pointon MA, Chew DM, Ovtcharova M, Sevastopulo GD, Crowley QG (2012). New high-precision U-Pb dates from western European carboniferous tuffs; implications for time scale calibration, the periodicity of late carboniferous cycles and stratigraphical correlation. J. Geol. Soc. Lond..

[33] Klug, C., Kröger, B., Vinther, J., Fuchs, D. & De Baets, K. in *Ammonoid paleobiology: from macroevolution to paleogeography* 3–24 (Springer, 2015).

[34] Sasaki, T., Shigeno, S. & Tanabe, K. in *Cephalopods—Present and Past* (Tanabe, K., Shigeta, Y., Sasaki, T. & Hirano, H. eds) 35–66 (Tokai University Press, 2010).

[35] Shigeno, S. et al. *in Cephalopods—Present Past*, 23–34 (Tokai University Press, 2010).

[36] Fuchs D, Keupp H, Schweigert G (2013). First record of a complete arm crown of the Early Jurassic coleoid Loligosepia (Cephalopoda). Palaontologische Z..

[37] Jereb, P., Roper, C. F. E., Norman, M. D. & Finn, J. K. *Cephalopods of the World, An Annotated and Illustrated Catalogue of Cephalopod Species Known to Date, Volume 3, Octopods and Vampire Squid*. (Food and Agriculture Organization of the United Nations, 2014).

[38] Fuchs D, Weis R (2010). Taxonomy, morphology and phylogeny of Lower Jurassic teudopseid coleoids (Cephalopoda). Neues Jahrb. f.ür. Geol. und Paläontologie - Abhandlungen.

[39] Doguzhaeva LA (2018). An Early Triassic gladius associated with soft tissue remains from Idaho, USA — a squid-like coleoid cephalopod at the onset of Mesozoic Era. Acta Palaeontol. Pol..

[40] Fuchs D (2020). The Muensterelloidea: phylogeny and character evolution of Mesozoic stem octopods. Pap. Palaeontol..

[41] Košťák M, Jagt JWM, Speijer RP, Stassen P, Steurbaut E (2013). New Paleocene sepiid coleoids (Cephalopoda) from Egypt: evolutionary significance and origin of the sepiid ‘rostrum’. PLoS ONE.

[42] Fuchs D, Donovan D (2018). Part M, Chapter 23C: systematic descriptions: Phragmoteuthida. Treatise Online.

[43] Warnke KM, Meyer A, Ebner B, Lieb B (2011). Assessing divergence time of Spirulida and Sepiida (Cephalopoda) based on hemocyanin sequences. Mol. Phylogenet. Evol..

[44] Jeletzky JA (1966). Comparative morphology, phylogeny, and classification of fossil Coleoidea. Univ. Kans. Paleontol. Contrib..

[45] Naef, A. Die fossilen tintenfische: eine paläozoologische monographie. 1–322 (*Jena Gustav Fischer,*1922).

[46] Doyle, P., Donovan, D. & Nixon, M. Phylogeny and systematics of the Coleoidea. (*Univ. Kansas Paleontol. Contrib*., 1994).

[47] Lindgren AR, Giribet G, Nishiguchi MK (2004). A combined approach to the phylogeny of Cephalopoda (Mollusca). Cladistics.

[48] Sanchez G (2018). Genus-level phylogeny of cephalopods using molecular markers: current status and problematic areas. PeerJ.

[49] Uribe JE, Zardoya R (2017). Revisiting the phylogeny of Cephalopoda using complete mitochondrial genomes. J. Mollusca. Stud..

[50] Lindgren AR, Pankey MS, Hochberg FG, Oakley TH (2012). A multi-gene phylogeny of Cephalopoda supports convergent morphological evolution in association with multiple habitat shifts in the marine environment. BMC Evol. Biol..

[51] Strugnell J, Norman M, Jackson J, Drummond AJ, Cooper A (2005). Molecular phylogeny of coleoid cephalopods (Mollusca: Cephalopoda) using a multigene approach; the effect of data partitioning on resolving phylogenies in a Bayesian framework. Mol. Phylogenet. Evol..

[52] Lakin JA, Marshall JEA, Troth I, Harding IC (2016). Greenhouse to icehouse: a biostratigraphic review of latest Devonian-Mississippian glaciations and their global effects. Geol. Soc. Spec. Publ..

[53] Bambach RK (2006). Phanerozoic biodiversity mass extinctions. Annu. Rev. Earth Planet. Sci..

[54] Royer DL, Berner RA, Montanez IP, Tabor NJ, Beerling DJ (2004). CO_2_ as a primary driver of Phanerozoic climate. GSA Today.

[55] Stein WE (2020). Mid-Devonian *Archaeopteris* roots signal revolutionary change in earliest fossil forests. Curr. Biol..

[56] Kiessling W (2009). Geologic and biologic controls on the evolution of reefs. Annu. Rev. Ecol. Evol. Syst..

[57] Whalen CD, Briggs DEG (2018). The Palaeozoic colonization of the water column and the rise of global nekton. Proc. R. Soc. B.

[58] Brett CE, Walker SE (2002). Predators and predation in Paleozoic marine environments. Paleontol. Soc. Pap..

[59] Klug C, Frey L, Pohle A, De Bates K, Korn D (2017). Palaeozoic evolution of animal mouthparts. Bull. Geosci..

[60] Friedman M, Sallan LC (2012). Five hundred million years of extinction and recovery: a phanerozoic survey of large-scale diversity patterns in fishes. Palaeontology.

[61] Korn, D. & Klug, C. in *Earth and Life: Global Biodiversity, Extinction Intervals and Biogeographic Perturbations Through Time* (ed. Talent, J. A.) 491–534 (Springer Science+Business Media, 2012).

[62] Whalen CD, Hull PM, Briggs DEG (2020). Paleozoic ammonoid ecomorphometrics test ecospace availability as a driver of morphological diversification. Sci. Adv..

[63] Alroy J (2008). Phanerozoic trends in the global diversity of marine invertebrates. Science.

[64] Fan J (2020). A high-resolution summary of Cambrian to Early Triassic marine invertebrate biodiversity. Science.

[65] Sallan LC, Coates MI (2010). End-Devonian extinction and a bottleneck in the early evolution of modern jawed vertebrates. Proc. Natl Acad. Sci. USA.

[66] McCoy, V. E. et al. Chemical signatures of soft tissues distinguish between vertebrates and invertebrates from the Carboniferous Mazon Creek Lagerstätte of Illinois. *Geobiology* **18**, 560–565 (2020). 10.1111/gbi.12397 32347003

[67] Lindsay DJ, Hunt JC, McNeil M, Beaman RJ, Vecchione M (2020). The first in situ observation of the ram’s horn squid *Spirula spirula* turns ‘common knowledge’ upside down. Diversity.

[68] Fuchs D, Lukeneder A (2014). Cenozoic coleoids (Cephalopoda) from Austria—a review of Schultz’s Catalogus Fossilium Austriae. Denisia.

[69] Yancey TE, Garvie CL (2011). Redescription of *Anomalosaepia* (Cephalopoda: Coleoida): a sepioid with a bimineralic calcite and aragonite skeleton. J. Paleontol..

[70] Doguzhaeva LA, Summesberger H (2012). Pro-ostraca of Triassic belemnoids (Cephalopoda) from Northern Calcareous Alps, with observations on their mode of preservation in an environment of northern Tethys which allowed for carbonization of non-biomineralized structures. Neues Jahrb. fur Geol. und Palaontologie - Abhandlungen.

[71] Arkhipkin AI, Bizikov VA, Fuchs D (2012). Vestigial phragmocone in the gladius points to a deepwater origin of squid (Mollusca: Cephalopoda). Deep. Res. I.

[72] Klug C, Schweigert G, Fuchs D, Kruta I, Tischlinger H (2016). Adaptations to squid-style high-speed swimming in Jurassic belemnitids. Biol. Lett..

[73] Johnson RG, Richardson ES (1968). Ten-armed fossil cephalopod from the Pennsylvanian of Illinois. Science.

[74] Iba Y, Sano S, Mutterlose J, Kondo Y (2012). Belemnites originated in the Triassic—a new look at an old group. Geology.

[75] Doguzhaeva LA (2000). A rare coleoid mollusc from the Upper Jurassic of Central Russia. Acta Palaeontol. Pol..

[76] Dzik J, Korn D (1992). Devonian ancestors of *Nautilus*. Paläontologische Z..

[77] Turek, V. in *Cephalopods - Present and Past*, 85–92 (Tokai University Press, 2010).

[78] King AH, Evans DH (2019). High-level classification of the nautiloid cephalopods: a proposal for the revision of the Treatise Part K. Swiss J. Palaeontol..

[79] Wade M (1988). Nautiloids and their descendants: cephalopod classification in 1986. N. Mex. Bur. Mines Miner. Resour. Mem..

[80] Holland CH (1987). The nautiloid cephalopods: a strange success. J. Geol. Soc. Lond..

[81] Teichert, C. *et al*. *Treatise on Invertebrate Paleontology, Part K, Mollusca 3: Cephalopoda—General Features, Endoceratoidea - Actinoceratoidea - Nautiloidea, Bactritoidea* (Geological Society of America and University of Kansas Press, 1964).

[82] Flower RH (1957). Nautiloids of the Paleozoic. Geol. Soc. Am. Mem..

[83] Aubrechtová M (2015). A revision of the Ordovician cephalopod *Bactrites sandbergeri* Barrande: systematic position and palaeobiogeography of *Bactroceras*. Geobios.

[84] Hildenbrand A, Austermann G, Fuchs D, Bengtson P, Stinnesbeck W (2021). A potential cephalopod from the early Cambrian of eastern Newfoundland, Canada. Commun. Biol..

[85] Fuchs D (2012). The ‘rostrum’ - problem in coleoid terminology—an attempt to clarify inconsistencies. Geobios.

[86] Doyle P, Shakides EV (2004). The Jurassic belemnite suborder Belemnotheutina. Palaeontology.

