# Supplementary material for: Fossil coleoid cephalopod from the Mississippian Bear Gulch Lagerstätte sheds light on early vampyropod evolution
Source: Nat Commun. 2022 Mar 8;13:1107. doi: 10.1038/s41467-022-28333-5 (PMC8904582; doi:10.1038/s41467-022-28333-5)
Supplement: Supplementary file 1 — Supplementary Information [file 41467_2022_28333_MOESM1_ESM.pdf]

Fossil coleoid cephalopod from the Mississippian Bear Gulch Lagerstätte sheds light on early vampyropod evolution

Christopher D. Whalen[1,2], Neil H. Landman[1]

[1]Department of Paleontology, American Museum of Natural History, New York, New York, 10024, USA. [2]Department of Earth and Planetary Sciences, Yale University, New Haven, Connecticut, 06511, USA. Correspondence and requests for materials should be addressed to C.D.W. (email: cwhalen@amnh.org).

SUPPLEMENTARY INFORMATION

I. Supplementary Figures……………………………..p2

II. Supplementary Methods..……………………….p10

III. Supplementary Notes………………………….p50

IV. Supplementary Discussion……………….....…..p59

V. Supplementary References…………………..……p62

I. SUPPLEMENTARY FIGURES

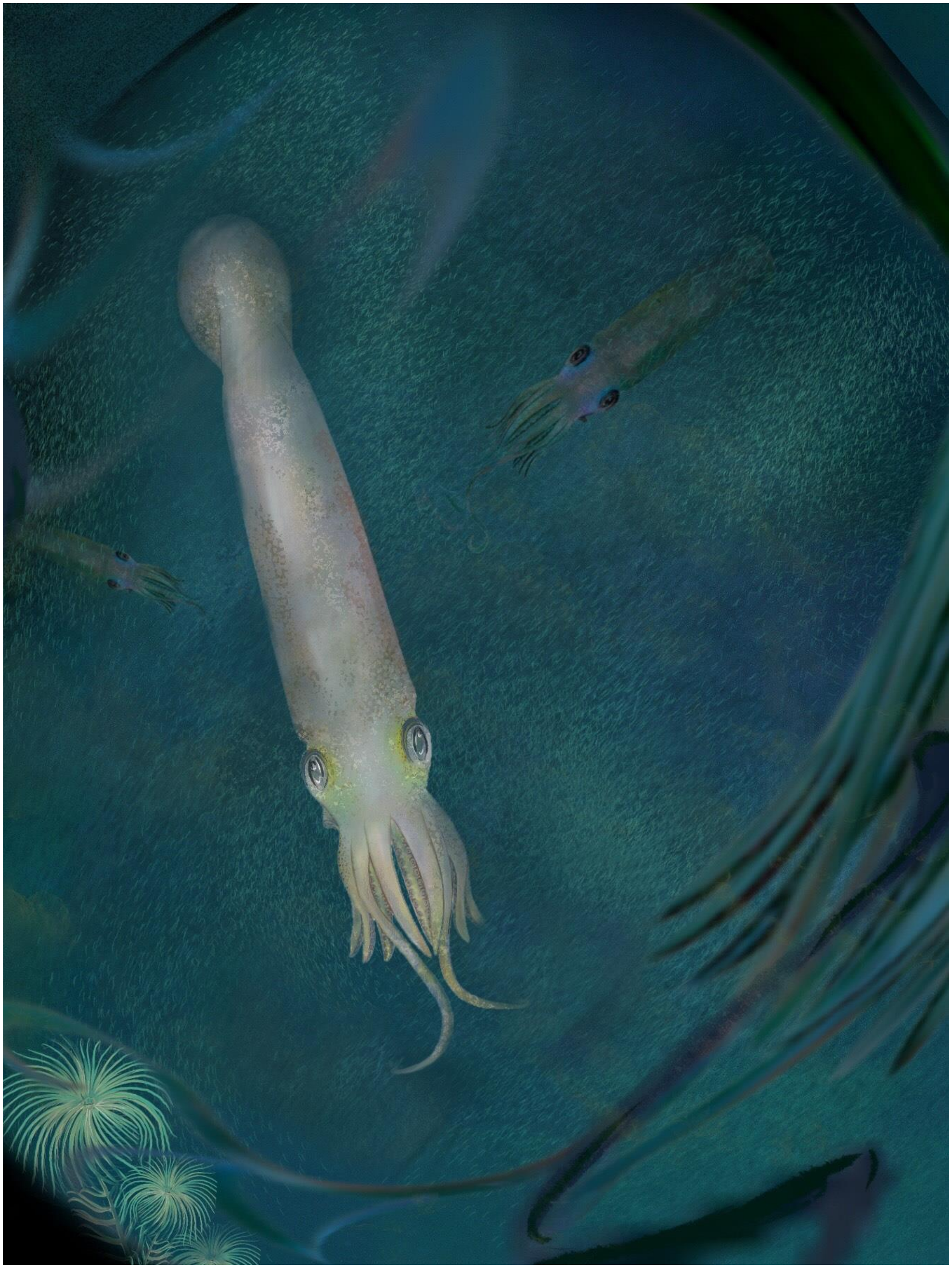

Supplementary Figure 1. Artistic reconstruction of *Syllipsimopodi bideni* gen. et sp. nov.; created by K. Whalen.

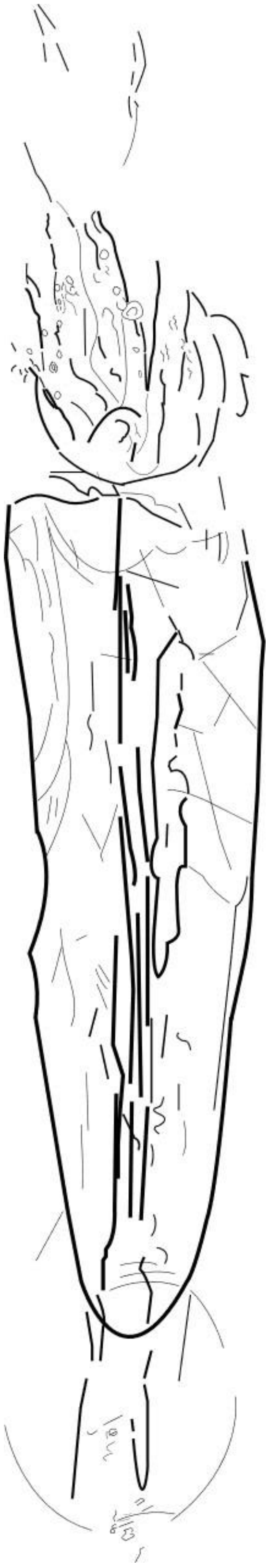

Supplementary Figure 2. Camera lucida drawing of *Syllipsimopodi bideni* gen. et sp. nov., holotype ROMIP 64897.

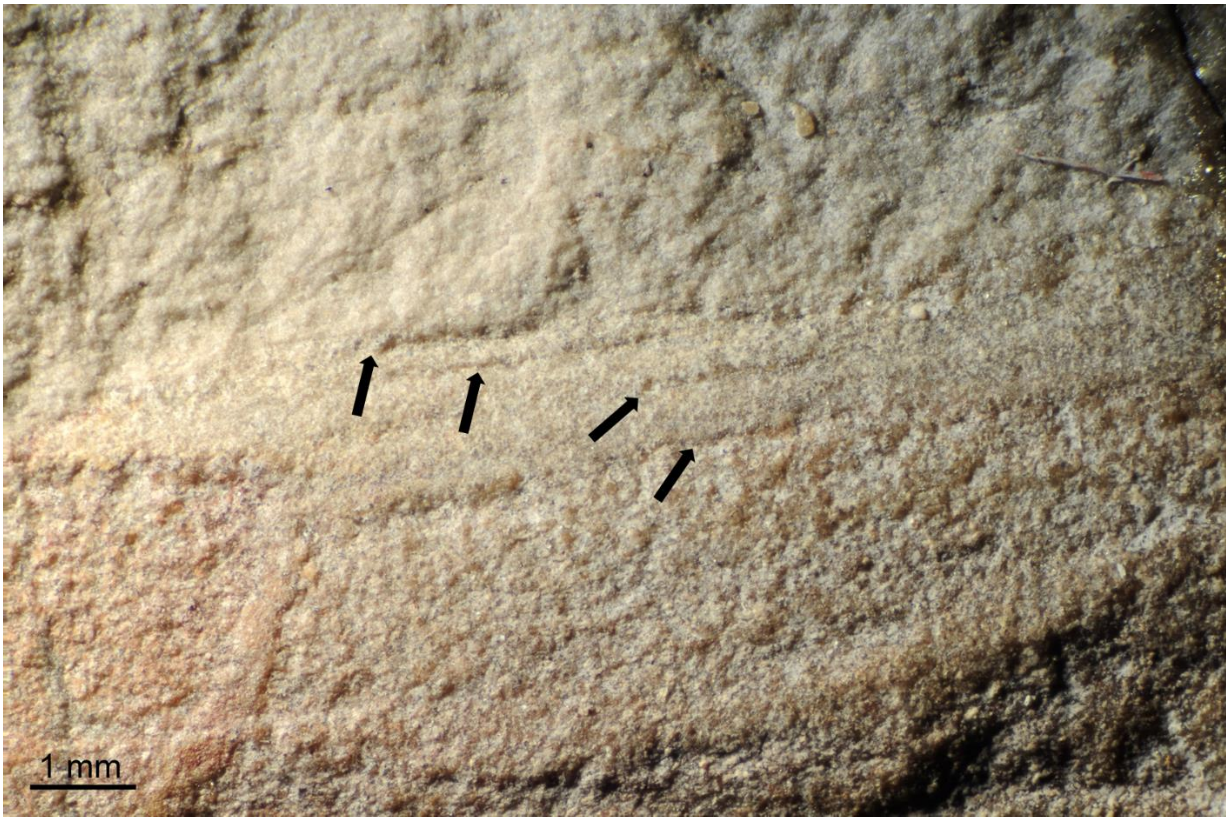

Supplementary Figure 3. *Syllipsimopodi bideni* gen. et sp. nov. (ROMIP 64897). Arrows pointing to growth lines on lateral edge of gladius.

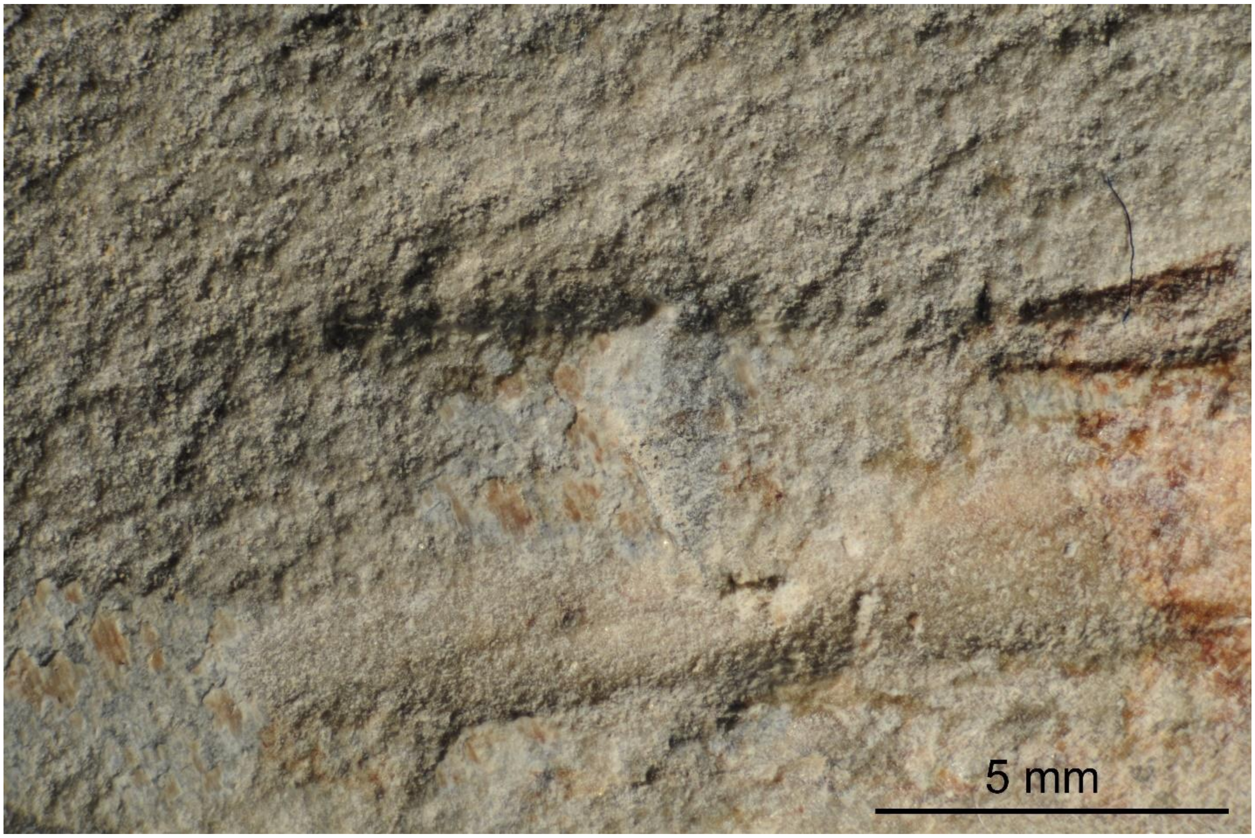

Supplementary Figure 4. Fin support and fibrous material (presumed connective tissues) of *Syllipsimopodi bideni* gen. et sp. nov. (ROMIP 64897).

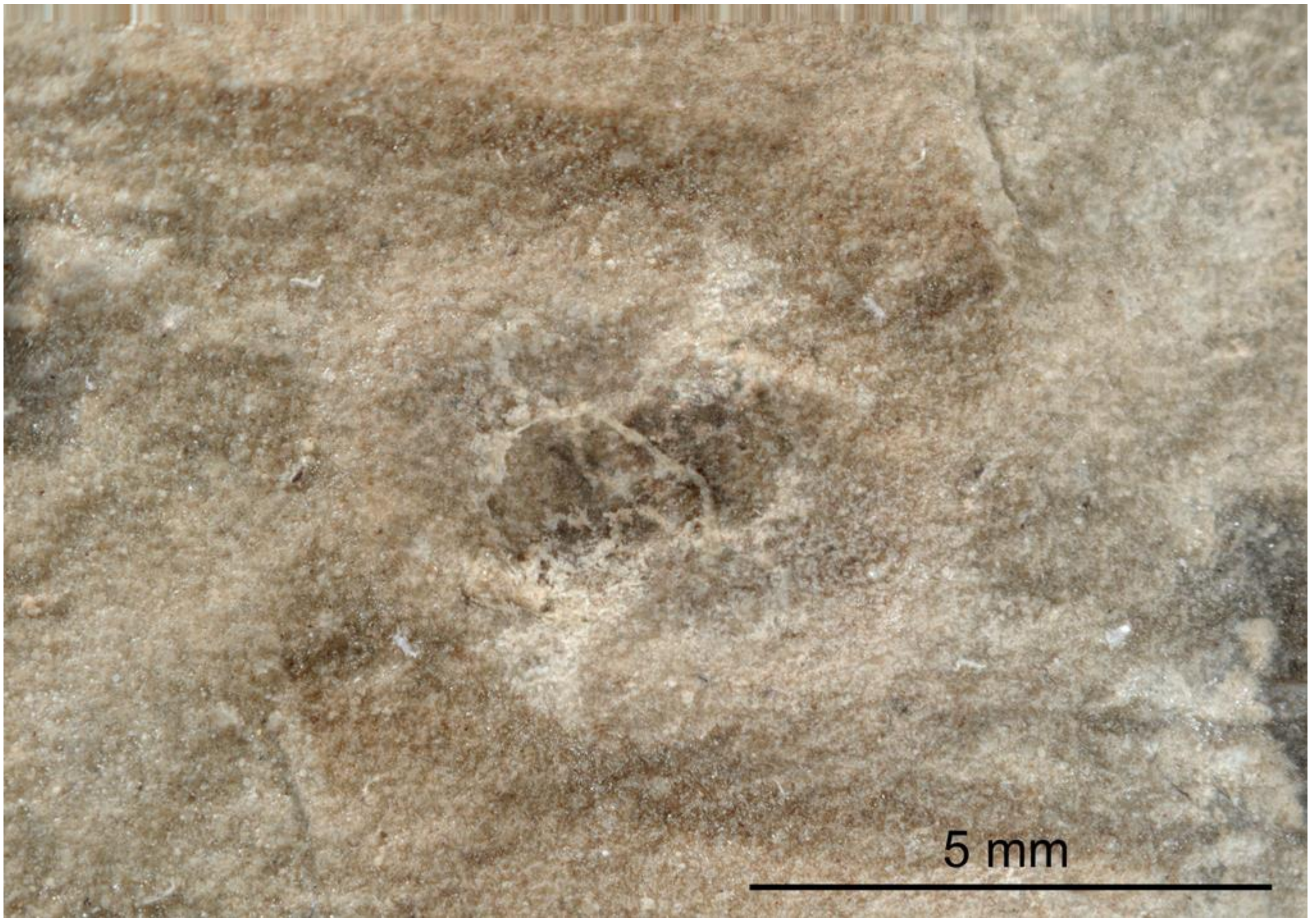

Supplementary Figure 5. Buccal apparatus of *Syllipsimopodi bideni* gen. et sp. nov. (ROMIP 64897).

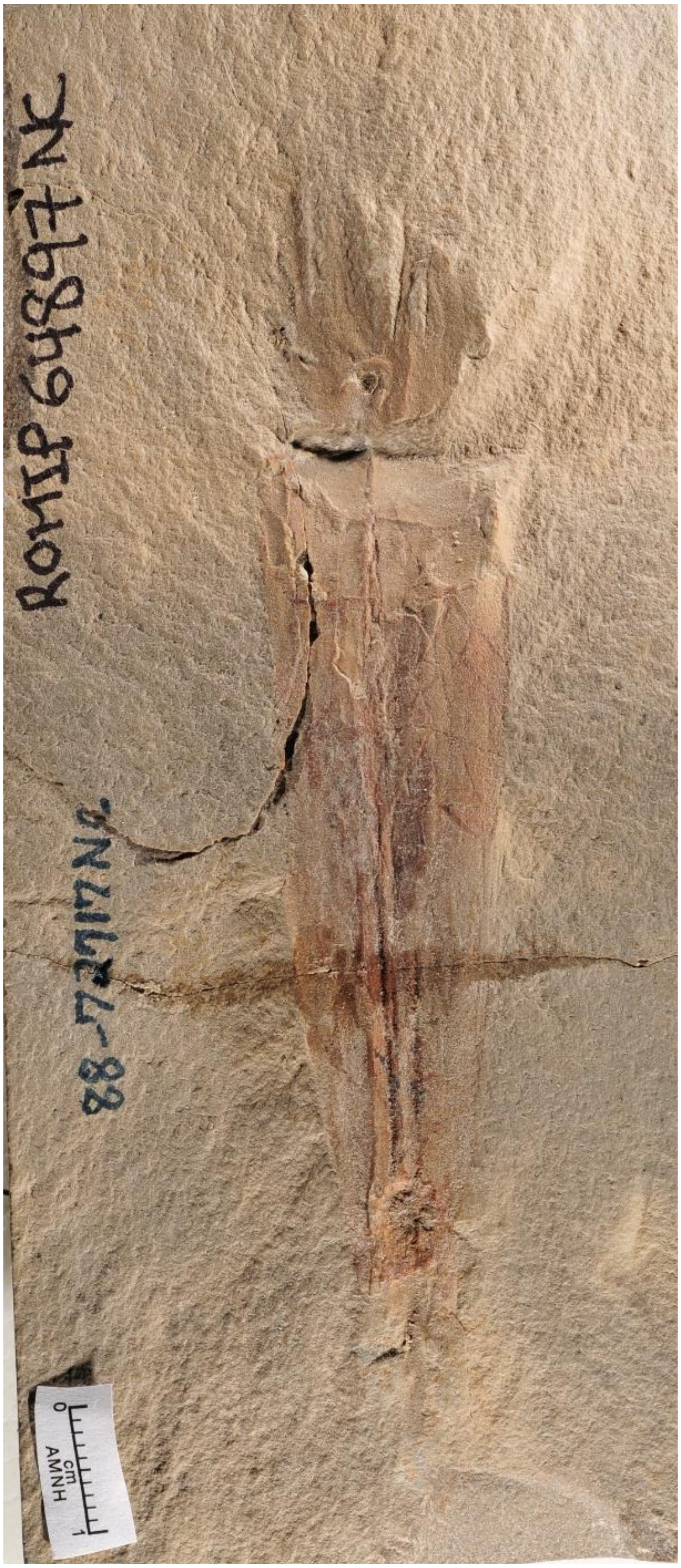

Supplementary Figure 6. *Syllipsimopodi bideni* gen. et sp. nov. (ROMIP 64897). Scale = 1cm.

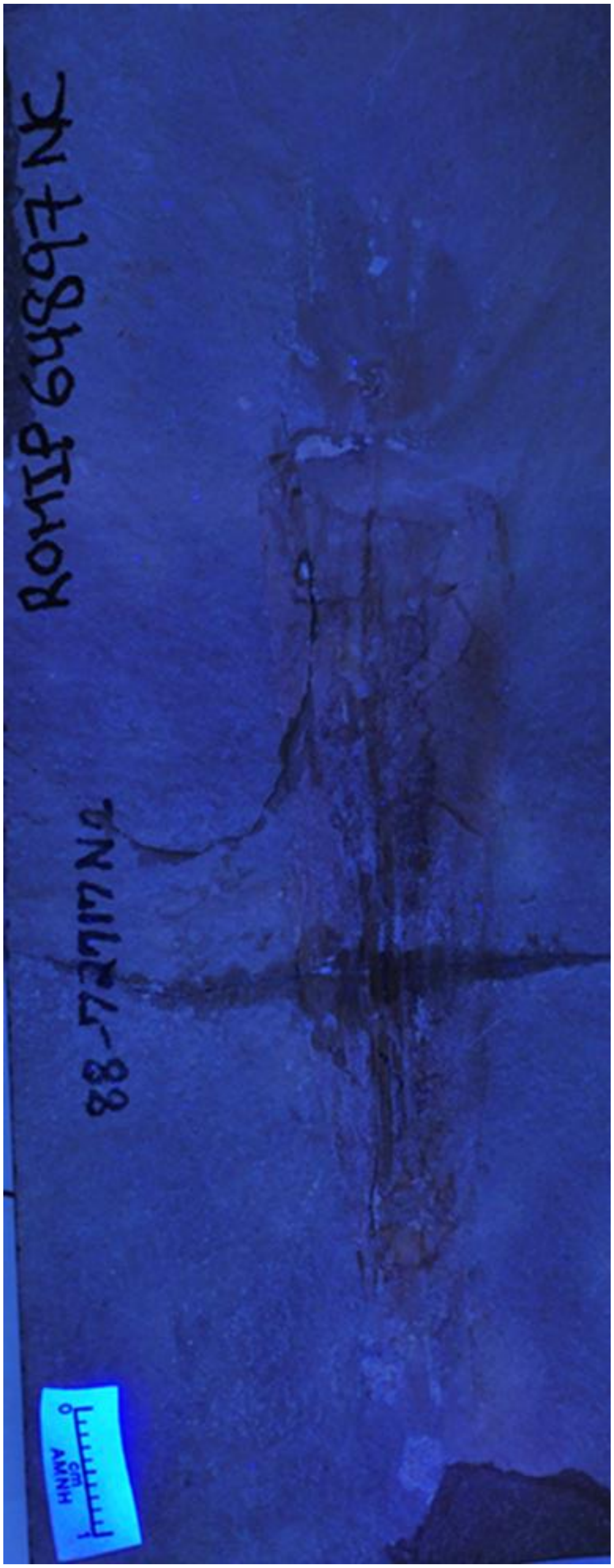

Supplementary Figure 7. *Syllipsimopodi bideni* gen. et sp. nov. (ROMIP 64897), photographed under UV light. Scale = 1cm.

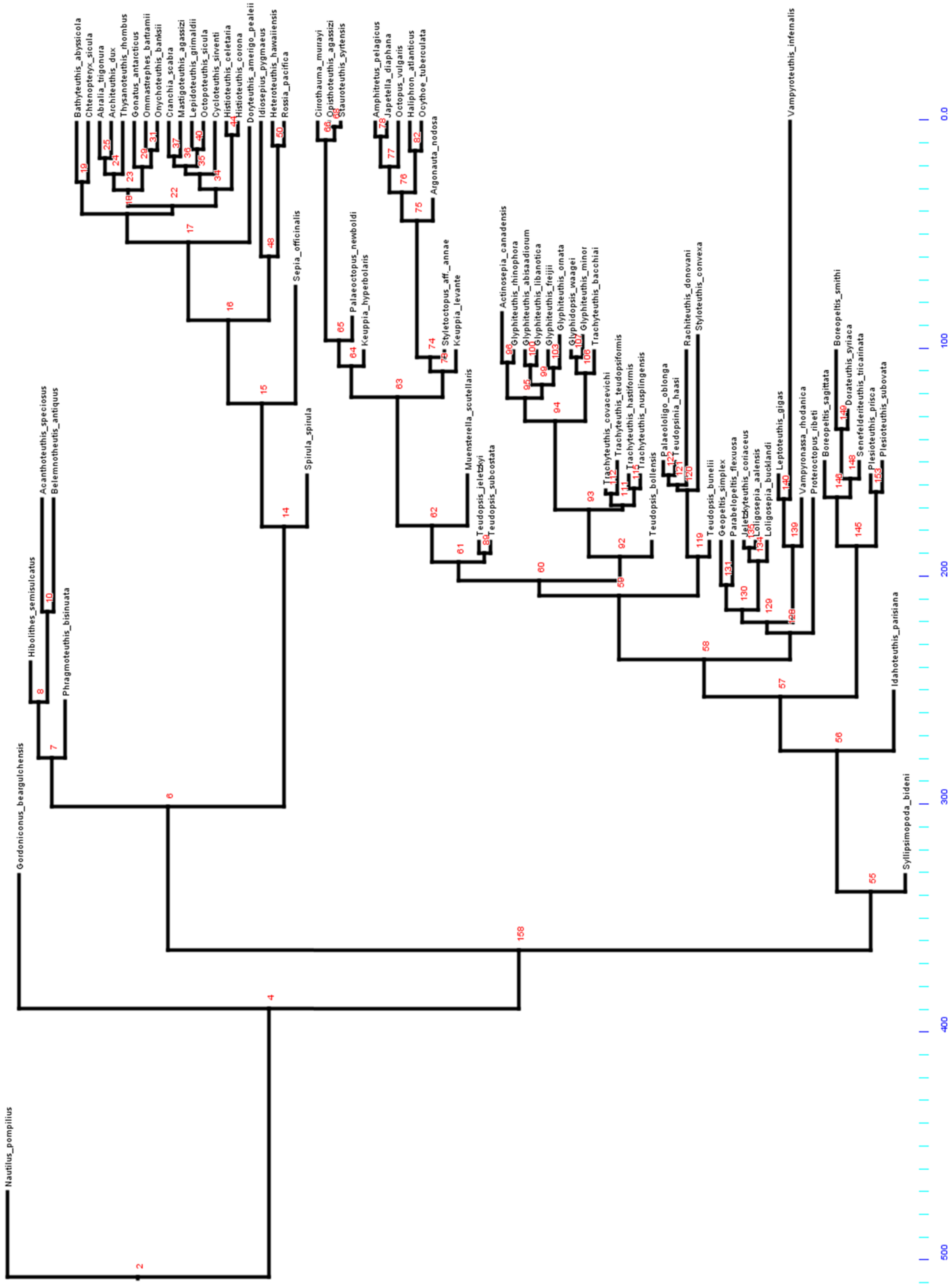

Supplementary Figure 8. Results of the Bayesian tip-dated phylogenetic analysis (Figure 6) with numbered nodes. Node numbers indicate character transitions (see Supplementary Notes). Source data are provided as a Source Data file.

II. SUPPLEMENTARY METHODS

Referenced list of all taxa included in the phylogenetic analysis.

SUBCLASS: Nautiloidea
- ORDER: Nautilida
  - *Nautilus pompilius*
    - Anatomy: Jereb & Roper, 2005; Kruta et al., 2016; Sutton et al., 2016
    - FAD: see *Centrocyrtoceras* (Dzik & Korn, 1992)
- ORDER: Barrandeocerida
  - *Centrocyrtoceras*
    - FAD: Ordovician, Darriwilian (Dzik & Korn, 1992)

SUBCLASS: Coleoidea
- ORDER: Donovanoconida
  - *Gordoniconus beargulchensis*
    - Anatomy: Klug et al., 2019
    - FAD: Carboniferous, Serpukhovian (Klug et al., 2019)
- SUPERORDER: Belemnoidea
  - ORDER: Phragmoteuthida
    - *Phragmoteuthis bisinuata*
      - Anatomy: Kruta et al., 2016; Sutton et al., 2016; Fuchs & Donovan, 2018
      - FAD: see *Permoteuthis* (Fuchs & Donovan, 2018)
    - *Permoteuthis*
      - FAD: Permian, Changhsingian (Fuchs & Donovan, 2018)
  - ORDER: Belemnoteuthidida
    - *Acanthoteuthis speciosus*
      - Anatomy: Klug et al., 2016
      - FAD: Jurassic, Callovian (Doyle & Shakides, 2004)
    - *Belemnotheutis antiquus*
      - Anatomy: Fuchs et al., 2007; Kruta et al., 2016; Sutton et al., 2016
      - FAD: Jurassic, Callovian (Fuchs et al., 2007a)
  - ORDER: Belemnitida
    - *Hibolithes semisulcatus*
      - Anatomy: Kruta et al., 2016; Sutton et al., 2016
      - FAD: see *Sichuanobelus* (Iba et al., 2012)
    - *Sichuanobelus*
      - FAD: Triassic, Carnian (Iba et al., 2012)
- SUPERORDER: Decabrachia
  - ORDER: Idiosepiida
    - *Idiosepius pygmaeus*
      - Anatomy: Jereb & Roper, 2005; Kruta et al., 2016; Sutton et al., 2016; Reid & Strugnell, 2018
      - FAD: Quaternary, Holocene, Recent

- ORDER: Sepiida
  - *Sepia officinalis*
    - Anatomy: Jereb & Roper, 2005; Kruta et al., 2016; Sutton et al., 2016
    - FAD: see *Ceratisepia* (Košťák et al., 2013)
  - *Ceratisepia*
    - FAD: Cretaceous, Maastrichtian (Košťák et al., 2013)
- ORDER: Sepiolida
  - *Heteroteuthis hawaiiensis*
    - Anatomy: Jereb & Roper, 2005; Kruta et al., 2016; Sutton et al., 2016
    - FAD: Quaternary, Holocene, Recent
  - *Rossia pacifica*
    - Anatomy: Jereb & Roper, 2005; Kruta et al., 2016; Sutton et al., 2016
    - FAD: Quaternary, Holocene, Recent
- ORDER: Myopsida
  - *Doryteuthis amerigo pealeii*
    - Anatomy: Jereb & Roper, 2010; Kruta et al., 2016; Sutton et al., 2016
    - FAD: Quaternary, Pleistocene (Neige et al., 2016)
- ORDER: Spirulida
  - *Spirula spirula*
    - Anatomy: Jereb & Roper, 2005; Kruta et al., 2016; Sutton et al., 2016; Oudot et al., 2020
    - FAD: see *Kostromateuthis* (Doguzhaeva, 2000)
  - *Kostromateuthis*
    - FAD: Jurassic, Kimmeridgian (Doguzhaeva, 2000)
- ORDER: Bathyteuthida
  - *Bathyteuthis abyssicola*
    - Anatomy: Jereb & Roper, 2010; Kruta et al., 2016; Sutton et al., 2016
    - FAD: Quaternary, Holocene, Recent
  - *Chtenopteryx sicula*
    - Anatomy: Jereb & Roper, 2010; Kruta et al., 2016; Sutton et al., 2016
    - FAD: Quaternary, Holocene, Recent
- ORDER: Oegopsida
  - *Abralia trigonura*
    - Anatomy: Jereb & Roper, 2010; Kruta et al., 2016; Sutton et al., 2016
    - FAD: Quaternary, Holocene, Recent
  - *Architeuthis dux*
    - Anatomy: Jereb & Roper, 2010; Kruta et al., 2016; Sutton et al., 2016

    - FAD: Quaternary, Holocene, Recent
  - *Cranchia scabra*
    - Anatomy: Jereb & Roper, 2010; Kruta et al., 2016; Sutton et al., 2016
    - FAD: Quaternary, Holocene, Recent
  - *Cycloteuthis sirventi*
    - Anatomy: Jereb & Roper, 2010; Kruta et al., 2016; Sutton et al., 2016
    - FAD: Quaternary, Holocene, Recent
  - *Gonatus antarcticus*
    - Anatomy: Arkhipin et al., 2012; Jereb & Roper, 2010; Kruta et al., 2016; Sutton et al., 2016
    - FAD: Quaternary, Holocene, Recent
  - *Histioteuthis celetaria*
    - Anatomy: Jereb & Roper, 2010; Kruta et al., 2016; Sutton et al., 2016
    - FAD: Quaternary, Holocene, Recent
  - *Histioteuthis corona*
    - Anatomy: Jereb & Roper, 2010; Kruta et al., 2016; Sutton et al., 2016
    - FAD: Quaternary, Holocene, Recent
  - *Lepidoteuthis grimaldii*
    - Anatomy: Jereb & Roper, 2010; Kruta et al., 2016; Sutton et al., 2016
    - FAD: Quaternary, Holocene, Recent
  - *Mastigoteuthis agassizi*
    - Anatomy: Jereb & Roper, 2010; Kruta et al., 2016; Sutton et al., 2016
    - FAD: Quaternary, Holocene, Recent
  - *Octopoteuthis sicula*
    - Anatomy: Jereb & Roper, 2010; Kruta et al., 2016; Sutton et al., 2016
    - FAD: Quaternary, Holocene, Recent
  - *Ommastrephes bartramii*
    - Anatomy: Jereb & Roper, 2010; Arkhipin et al., 2012; Kruta et al., 2016; Sutton et al., 2016
    - FAD: Quaternary, Holocene, Recent
  - *Onychoteuthis banksii*
    - Anatomy: Jereb & Roper, 2010; Kruta et al., 2016; Sutton et al., 2016
    - FAD: Quaternary, Holocene, Recent
  - *Thysanoteuthis rhombus*
    - Anatomy: Jereb & Roper, 2010; Kruta et al., 2016; Sutton et al., 2016
    - FAD: Quaternary, Holocene, Recent

CLADE: Vampyropoda

- *Syllipsimopodi bideni* gen. et sp. nov.
- *Idahoteuthis parisiana*
    - Anatomy: Doguzhaeva et al., 2018
    - FAD: Triassic, Olenekian (Doguzhaeva et al., 2018)

- SUPERORDER: Octobrachia
    - *Proteroctopus ribeti*
        - Anatomy: Kruta et al., 2016
        - FAD: Jurassic, Callovian (Fuchs, 2020)
    - SUBORDER: Loligosepiina
        - *Geopeltis simplex*
            - Anatomy: Kruta et al., 2016; Sutton et al., 2016; Fuchs, 2020
            - FAD: Jurassic, Toarcian (Fuchs, 2020)
        - *Jeletzkyteuthis coriaceus*
            - Anatomy: Kruta et al., 2016; Sutton et al., 2016; Fuchs, 2020
            - FAD: Jurassic, Toarcian (Fuchs, 2020)
        - *Leptoteuthis gigas*
            - Anatomy: Kruta et al., 2016; Sutton et al., 2016; Fuchs, 2020
            - FAD: Jurassic, Kimmeridgian (Fuchs, 2020)
        - *Loligosepia aalensis*
            - Anatomy: Kruta et al., 2016; Sutton et al., 2016; Fuchs, 2020
            - FAD: Jurassic, Toarcian (Fuchs & Weis, 2008)
        - *Loligosepia bucklandi*
            - Anatomy: Kruta et al., 2016; Sutton et al., 2016
            - FAD: Jurassic, Toarcian (Fuchs & Weis, 2008)
        - *Parabelopeltis flexuosa*
            - Anatomy: Kruta et al., 2016; Sutton et al., 2016; Fuchs, 2020
            - FAD: Jurassic, Toarcian (Fuchs, 2020)
    - SUBORDER: Prototeuthidina
        - *Boreopeltis sagittata*
            - Anatomy: Kruta et al., 2016; Sutton et al., 2016; Fuchs, 2020
            - FAD: Jurassic, Tithonian (Fuchs, 2020)
        - *Boreopeltis smithi*
            - Anatomy: Kruta et al., 2016; Sutton et al., 2016; Fuchs, 2020
            - FAD: Cretaceous, Cenomanian (Fuchs & Larson, 2011a)
        - *Dorateuthis syriaca*
            - Anatomy: Kruta et al., 2016; Sutton et al., 2016; Fuchs, 2020
            - FAD: Cretaceous, Barremian (Fuchs, 2020)
        - *Plesioteuthis prisca*
            - Anatomy: Fuchs et al., 2007; Kruta et al., 2016; Sutton et al., 2016
            - FAD: Jurassic, Kimmeridgian (Fuchs et al., 2007b)
        - *Plesioteuthis subovata*
            - Anatomy: Fuchs et al., 2007; Kruta et al., 2016; Sutton et al., 2016
            - FAD: Jurassic, Tithonian (Fuchs et al., 2007b)
        - *Senefelderiteuthis tricarinata*

        - Anatomy: Kruta et al., 2016; Sutton et al., 2016; Fuchs, 2020
        - FAD: Jurassic, Tithonian (Fuchs, 2020)
- SUBORDER: Teudopseina
    - *Actinosepia canadensis*
        - Anatomy: Kruta et al., 2016; Sutton et al., 2016; Fuchs, 2020
        - FAD: Cretaceous, Campanian (Fuchs, 2020)
    - *Glyphidopsis waagei*
        - Anatomy: Kruta et al., 2016; Sutton et al., 2016; Fuchs, 2020
        - FAD: Cretaceous, Cenomanian (Fuchs, 2020)
    - *Glyphiteuthis abisaadiorum*
        - Anatomy: Kruta et al., 2016; Sutton et al., 2016
        - FAD: Cretaceous, Cenomanian (Fuchs, 2020)
    - *Glyphiteuthis freijii*
        - Anatomy: Kruta et al., 2016; Sutton et al., 2016; Fuchs, 2020
        - FAD: Cretaceous, Cenomanian (Fuchs, 2020)
    - *Glyphiteuthis libanotica*
        - Anatomy: Kruta et al., 2016; Sutton et al., 2016
        - FAD: Cretaceous, Cenomanian (Fuchs, 2020)
    - *Glyphiteuthis minor*
        - Anatomy: Kruta et al., 2016; Sutton et al., 2016
        - FAD: Cretaceous, Turonian (Košťák, 2002)
    - *Glyphiteuthis ornata*
        - Anatomy: Kruta et al., 2016; Sutton et al., 2016; Fuchs, 2020
        - FAD: Cretaceous, Turonian (Fuchs, 2020)
    - *Glyphiteuthis rhinophora*
        - Anatomy: Kruta et al., 2016; Sutton et al., 2016
        - FAD: Cretaceous, Cenomanian (Fuchs, et al., 2010)
    - *Muensterella scutellaris*
        - Anatomy: Kruta et al., 2016; Sutton et al., 2016; Fuchs, 2020
        - FAD: Jurassic, Kimmeridgian (Fuchs, 2020)
    - *Palaeololigo oblonga*
        - Anatomy: Kruta et al., 2016; Sutton et al., 2016; Fuchs, 2020
        - FAD: Jurassic, Tithonian (Fuchs, 2020)
    - *Rachiteuthis donovani*
        - Anatomy: Kruta et al., 2016; Sutton et al., 2016; Fuchs, 2020
        - FAD: Cretaceous, Cenomanian (Fuchs, 2020)
    - *Styloteuthis convexa*
        - Anatomy: Kruta et al., 2016; Sutton et al., 2016
        - FAD: Cretaceous, Turonian (Fuchs, 2020)
    - *Teudopsinia haasi*
        - Anatomy: Kruta et al., 2016; Sutton et al., 2016
        - FAD: Jurassic, Tithonian (Fuchs, 2020)
    - *Teudopsis bollensis*
        - Anatomy: Kruta et al., 2016; Sutton et al., 2016
        - FAD: Jurassic, Toarcian (Fuchs, 2020)

- *Teudopsis bunelii*
  - Anatomy: Kruta et al., 2016; Sutton et al., 2016; Fuchs, 2016
  - FAD: Jurassic, Toarcian (Fuchs & Weis, 2010)
- *Teudopsis jeletzkyi*
  - Anatomy: Kruta et al., 2016; Sutton et al., 2016
  - FAD: Jurassic, Toarcian (Riccardi, 2005)
- *Teudopsis subcostata*
  - Anatomy: Kruta et al., 2016; Sutton et al., 2016; Fuchs, 2016
  - FAD: Jurassic, Toarcian (Fuchs & Weis, 2010)
- *Trachyteuthis bacchiai*
  - Anatomy: Kruta et al., 2016; Sutton et al., 2016
  - FAD: Cretaceous, Cenomanian (Fuchs & Larson, 2011b)
- *Trachyteuthis covacevichi*
  - Anatomy: Kruta et al., 2016; Sutton et al., 2016
  - FAD: Jurassic, Oxfordian (Fuchs & Schultze, 2008)
- *Trachyteuthis hastiformis*
  - Anatomy: Kruta et al., 2016; Sutton et al., 2016; Fuchs, 2020
  - FAD: Jurassic, Kimmeridgian (Fuchs et al., 2007c)
- *Trachyteuthis nusplingensis*
  - Anatomy: Kruta et al., 2016; Sutton et al., 2016
  - FAD: Jurassic, Kimmeridgian (Fuchs, 2020)
- *Trachyteuthis teudopsiformis*
  - Anatomy: Kruta et al., 2016; Sutton et al., 2016; Fuchs, 2020
  - FAD: Jurassic, Tithonian (Fuchs et al., 2007c)

- ORDER: Vampyromorphida
  - *Vampyronassa rhodanica*
    - Anatomy: Kruta et al., 2016; Sutton et al., 2016; Fuchs, 2020
    - FAD: Jurassic, Callovian (Fuchs, 2020)
  - *Vampyroteuthis infernalis*
    - Anatomy: Jereb et al., 2014; Fuchs & Iba, 2015; Kruta et al., 2016; Sutton et al., 2016
    - FAD: Quaternary, Holocene, Recent
- ORDER: Cirrata
  - *Cirrothauma murrayi*
    - Anatomy: Jereb et al., 2014; Kruta et al., 2016; Sutton et al., 2016
    - FAD: Quaternary, Holocene, Recent
  - *Opisthoteuthis agassizi*
    - Anatomy: Jereb et al., 2014; Kruta et al., 2016; Sutton et al., 2016
    - FAD: Quaternary, Holocene, Recent
  - *Stauroteuthis syrtensis*
    - Anatomy: Jereb et al., 2014; Kruta et al., 2016; Sutton et al., 2016
    - FAD: Quaternary, Holocene, Recent
- ORDER: Incirrata
  - *Amphitretus pelagicus*
    - Anatomy: Jereb et al., 2014; Kruta et al., 2016; Sutton et al., 2016

- FAD: Quaternary, Holocene, Recent

- *Argonauta nodosa*
  - Anatomy: Jereb et al., 2014; Kruta et al., 2016; Sutton et al., 2016
  - FAD: see *Obinautilus* (Fuchs, 2020)
- *Obinautilus*
  - FAD: Paleogene, Rupelian (Fuchs, 2020)
- *Haliphron atlanticus*
  - Anatomy: Jereb et al., 2014; Kruta et al., 2016; Sutton et al., 2016
  - FAD: Quaternary, Holocene, Recent
- *Japetella diaphana*
  - Anatomy: Jereb et al., 2014; Kruta et al., 2016; Sutton et al., 2016
  - FAD: Quaternary, Holocene, Recent
- *Keuppia hyperbolaris*
  - Anatomy: Kruta et al., 2016; Sutton et al., 2016; Fuchs, 2020
  - FAD: Cretaceous, Cenomanian (Fuchs, 2020)
- *Keuppia levante*
  - Anatomy: Kruta et al., 2016; Sutton et al., 2016; Fuchs, 2020
  - FAD: Cretaceous, Cenomanian (Fuchs, 2020)
- *Octopus vulgaris*
  - Anatomy: Jereb et al., 2014; Kruta et al., 2016; Sutton et al., 2016
  - FAD: Quaternary, Holocene, Recent
- *Ocythoe tuberculata*
  - Anatomy: Jereb et al., 2014; Kruta et al., 2016; Sutton et al., 2016
  - FAD: Quaternary, Holocene, Recent
- *Palaeoctopus newboldi*
  - Anatomy: Kruta et al., 2016; Sutton et al., 2016; Fuchs, 2020
  - FAD: Cretaceous, Santonian (Fuchs, 2020)
- *Styletoctopus aff. annae*
  - Anatomy: Kruta et al., 2016; Sutton et al., 2016; Fuchs, 2020
  - FAD: Cretaceous, Cenomanian (Fuchs, 2020)

Referenced list of all characters included in the phylogenetic analysis. The character list is based on the Sutton et al. (2016) phylogeny that was expanded by Kruta et al. (2016). Sutton et al (2016) relied on the phylogenies of Lindgren et al. (2004) and Young and Vecchione (1996). Modifications to the cited characters and their coding, as well as new characters, are discussed below. Otherwise, see Sutton et al. (2016) for character descriptions. Although Sutton et al. (2016) designed and coded some characters as contingent on others, they did not always explicitly list the relevant character dependencies in their character descriptions. Additionally, several cited characters are contingent on others, but they were not coded in a contingency framework by Sutton et al. (2016) and Kruta et al. (2016). We explicitly list all character dependencies below and have recoded some characters to better reflect these dependencies. For most characters, this only involved replacing absent (0) with inapplicable (-), although in some cases more substantial changes were necessitated. We also split most non-continuous ordered characters into nested contingent characters, which are less error prone (Brazeau, 2011). Although most characters are listed in the same order as in Kruta et al. (2016), some characters have been split, deleted, or in rare cases reshuffled, and new characters are inserted into the list when logical.

1. Shell/gladius/vestigial shell: absent (0); present (1)
    - Sutton et al. (2016) #0

2. Shell location in relation to the rest of the body: external (0); internal (1)
    - Sutton et al. (2016) #1
    - Dependency: Shell/gladius/vestigial shell = present (1)

3. Shell extent along A-P axis: anterior half (0); posterior half (1); whole or most of the body length (2)
    - Sutton et al. (2016) #2
    - Dependency: Shell location in relation to the rest of the body = internal (1)

4. Shell extent along D-V axis: dorsal half (0); whole or most of the body height (1)
    - Sutton et al. (2016) #3
    - Dependency: Shell location in relation to the rest of the body = internal (1)

5. Discrete proostracum developed: no (0); yes (1)
    - Sutton et al. (2016) #5
    - Dependency: Shell/gladius/vestigial shell = present (1)
    - Discussion: We maintain that the proostracum is homologous to the gladius; however, we do not accept that the gladius/proostracum is a dorsal extension of the conotheca from a vestigial living chamber. Doguzhaeva and Summesberger (2012) convincingly demonstrated that the belemnoid proostracum is a lamello-organic layer situated between the mineralized inner conotheca and mineralized outer primordial rostrum and rostrum. Thus, the proostracum is clearly distinct from the conotheca and cannot be homologized with a dorsal extension of it. Sutton et al. (2016) and Kruta et al. (2016) considered the proostracum homologous to the cuttlebone owing to the similar gross morphology. This was problematic in the traditional view of proostracal homology because the

cuttlebone is not a gladius, but a heavily modified phragmocone, where the ventral surface has been reduced and successive chambers are closely packed and vaulted above one another (Fuchs & Iba, 2015). However, this problem disappears once the proposed homology of the proostracum with the dorsal living chamber is rejected. In sepiids, the proostracal layer likely remains external to and overlying the modified phragmocone; it does not extend anterior to it. Discussions of sepiid proostracal anatomy would be therefore be valid.

6. Anterodorsally extended proostracum: absent (0); present (1)
    - New Character
    - Dependency: Discrete proostracum developed: yes (1)
    - This character is intended to describe the common condition whereby the proostracum extends far anterior of the phragmocone and living chamber. *Gordoniconus* is coded as absent; all other proostracum/gladius bearing taxa except for *Sepia* are coded as present.

7. Septate phragmocone: absent (0); present (1)
    - Sutton et al. (2016) #7
    - Dependency: Shell/gladius/vestigial shell = present (1)
    - Discussion: Since the proostracum is not homologous to the conotheca, the conus is also not homologous to the phragmocone. Instead, the conus is what remains of the proostracal layer that coated the phragmocone at the apex. The distinction between septate and non-septate phragmocones is thus meaningless; all phragmocones are septate. This character now refers to the presence/absence of a phragmocone, the coding of which is equivalent to Sutton et al.'s (2016) and Kruta et al.'s (2016) coding for the presence/absence of septa.

8. Mineralized phragmocone: absent (0); present (1)
    - Sutton et al. (2016) #4
    - Dependency: Septate phragmocone = present (1)
    - Discussion: Character redefined from Sutton et al. (2016) "Calcium carbonate in the shell" since it is highly uncertain which fossil gladii, if any, were originally mineralized rather than diagenetically altered (Donovan, 2016).

9. Siphuncle linking chambers, or homologous structure: absent (0); present (1)
    - Sutton et al. (2016) #8
    - Dependency: Septate phragmocone = present (1)
    - Discussion: Recoded as present in all belemnoids and inapplicable in all vampyropods, including *Vampyroteuthis*. Coded as absent in *Ommastrephes* (Arkhipin et al., 2012).

10. Position of siphuncle within the shell: ventral (0); central (1)
    - Sutton et al. (2016) #9
    - Dependency: Siphuncle linking chambers, or homologous structure = present (1)

11. Median field (or homologous rachis): absent (0); present (1)

- Sutton et al. (2016) #11
- Dependency: Discrete proostracum developed = yes (1)

12. Distinct hyperbolar zones (or homologous vanes): absent (0); present (1)
    - Sutton et al. (2016) #12
    - Dependency: Discrete proostracum developed = yes (1)
    - Discussion: Added the clarification "distinct" because technically several taxa have hyperbolar zones (such as the prototeuthids), but they are so thin that they can and should be treated as absent. Recoded from "?" to present in *Proteroctopus* and to absent in *Hibolithes* and *Plesioteuthis subovata.*

13. Lateral fields (or homologous wings): absent (0); present (1)
    - Sutton et al. (2016) #13
    - Dependency: Discrete proostracum developed = yes (1)
    - Discussion: Recoded from "?" to absent in *Plesioteuthis subovata.*

14. Primordial rostrum: absent (0); present (1)
    - New Character
    - Dependency: Shell location in relation to the rest of the body = internal (1)
    - Discussion: Many structures termed "rostra" in the literature are actually primordial rostra; the rostrum is a distinct calcitic or aragonitic structure deposited on the primordial rostrum (Fuchs, 2012). True rostra are only known from aulacocerids, belemnitids, belemnoteuthidids, and sepiids (Yancey et al., 2011; Fuchs, 2012; Košťák et al., 2013). Fuchs (2012) considered the spirulid and sepiid sheaths to be homologous to the primordial rostrum; however, some fossil sepiids clearly possess a bimineralic sheath composed of an underlying aragonitic primordial rostrum and overlying outer calcitic rostrum (Yancey et al., 2011; Košťák et al., 2013). Extant cuttlefish have apparently lost this outer calcitic layer. The unmineralized "rostrum" of some extant decabrachians is considered homologous with a primordial rostrum. The "outer plate" of spirulids is sometimes compared to the outer prismatic layer of the conotheca, but it is more likely to be a primordial rostrum since it is microtuberculated like other primordial rostra and since it overlies the periostracum (Doguzhaeva, 1996; Oudot et al., 2020). We code *Spirula* as present.

15. Mineralized primordial rostrum: absent (0); present (1)
    - New Character
    - Dependency: Primordial rostrum = present (1)

16. Primordial rostrum length: short, up to 25% of the shell length (0); strongly developed, 25% or more of the shell length (1)
    - Sutton et al. (2016) #15
    - Dependency: Primordial rostrum = present (1)
    - Discussion: Rephrased to refer to the more widely distributed primordial rostrum as opposed to the less common rostrum proper.

17. Rostrum or guard: absent (0); present (1)
    - Sutton et al. (2016) #14
    - Dependency: Shell location in relation to the rest of the body = internal (1)
    - Discussion: A tertiary formation deposited on the secondary primordial rostrum, sensu Fuchs 2012.

18. Conus (primary cone): absent (0); present (1)
    - Sutton et al. (2016) #16
    - Dependency: Discrete proostracum developed = yes (1)
    - Discussion: Since we do not consider the proostracum to be homologous to the phragmocone conotheca, the conus is also not homologous to (or a remnant of) the phragmocone. We consider the proostracum to be a distinct tissue layer enveloping the conotheca. So, rather than a vestigial phragmocone without septa, the conus is instead the apical portion of the proostracal layer. Under this framework, the conus never possessed septa since the proostracal layer does not appear to line the internal surfaces of the phragmocone. The coding of this character is relatively unchanged despite our revised homology statement. This is because all taxa with a proostracum layer and a phragmocone are assumed to possess the proostracum layer at the apex, which is a conus (albeit a conus infilled by the phragmocone). All extant octopods retaining a gladius vestige have a conus if the right and left lateral halves are posteriorly connected (as they are in cirrates). *Nautilus*, which lacks a proostracum, is recoded as inapplicable. Recoded from "?" to present in *Proteroctopus, Jeletzkyteuthis, Trachyteuthis hastiformis, Trachyteuthis teudopsiformis, Teudopsis subcostata, Teudopsis bunelii, Muensterella, Actinosepia, Glyphidopsis, Geopeltis, Parabelopeltis, Glyphiteuthis freijii, Glyphiteuthis ornata,* and *Vampyronassa*. Recoded from "?" to present in *Plesioteuthis subovata;* we consider the posterior preservation of the neotype sufficient for this determination, contra Sutton et al. (2014) and Fuchs et al. (2007b). *Cranchia* was mistakenly coded absent, this has been corrected (Jereb & Roper, 2010). *Palaeololigo* and *Glyphiteuthis libanotica* were also incorrectly coded as absent; they have been changed to present.

19. Shell coiled: no (0); yes, endogastric (1); yes, exogastric (2)
    - Sutton et al. (2016) #17
    - Dependency: Shell/gladius/vestigial shell = present (1)
    - Discussion: State 2 added to distinguish the exogastric nautilid coiling from the independently evolved endogastric decabrachian coiling. As previously defined, the character incorrectly implied that *Spirula* was plesiomorphic for this trait. *Sepia* recoded as endogastrically coiled despite not completing a full volution (the unique morphology of the cuttlebone results from this partial endogastric coiling).

20. Condition of the primary cone: funnel-like cone (0); cup-like cone (1)
    - Sutton et al. (2016) #20
    - Dependency: Conus (primary cone) = present (1)

- Discussion: Reversed implied polarity since this character is now part of a series. *Proteroctopus* has a cup like conus morphologically similar to *Vampyroteuthis*. *Dorateuthis* recoded as funnel-like from unknown. *Parabelopeltis* recoded as cup-like from unknown.

21. Primary cone open ventrally: absent (0); present (1)
    - Sutton et al. (2016) #19
    - Dependency: Condition of the primary cone = cup-like cone (1)

22. Patella: absent (0); present (1)
    - New Character
    - Dependency: Primary cone open ventrally = present (1)
    - Discussion: Extreme opening of the conus, such that the adapical ventral lip of the conus is positioned posterior of the conus apex. This character more clearly captures the information from the former character "Median asymptotes posterior intersection: intersect at or very near to the posterior margin of the gladius (0); intersect within gladius (1)."

23. 'Cone flags': absent (0); present (1)
    - Sutton et al. (2016) #22
    - Dependency: Discrete proostracum developed = yes (1)
    - Discussion: Recoded from "?" to present in *Proteroctopus* and *Dorateuthis,* and from present to absent in *Leptoteuthis*. However, we echo Sutton et al.'s (2016) concern that this character is poorly defined.

24. Ventral folding of posterolateral gladius margin: flat, not folded (0); folded (1)
    - Sutton et al. (2016) #23
    - Dependency: Discrete proostracum developed = yes (1)
    - Discussion: *Muensterella* corrected from 1 to 0. *Belemnotheutis, Opisthoteuthis, Dorateuthis, Stauroteuthis, Haliphron, Octopus, Keuppia, Palaeoctopus,* and *Styletoctopus* recoded as "0" from "?"

25. Ventral folding of posterolateral gladius margin: folded but not fused (e.g., 'pseudocone') (0); folded and fused ventrally ('secondary cone') (1)
    - Sutton et al. (2016) #23
    - Dependency: Ventral folding of posterolateral gladius margin = folded (1)

26. Gladius length / gladius width: < 2 (0); ≥ 2 (1); ≥ 3 (2); ≥ 4 (3); ≥ 5 (4); ≥ 10 (5) [ordered character]
    - Sutton et al. (2016) #24
    - Dependency: Discrete proostracum developed = yes (1)
    - Discussion: Former state 0 (< 1) and 1 (≥ 1) combined into new state 0 (< 2). Recoded to inapplicable for *Nautilus,* and from unknown for *Proteroctopus* and *Vampyronassa.*

27. Vane length / rachis length: < 0.3 (0); ≥ 0.3 (1); ≥ 0.5 (2); ≥ 0.7 (3); ≥ 0.9 (4) [ordered character]
    - Sutton et al. (2016) #25
    - Dependency: Median field (or homologous rachis) = present (1)
    - Dependency: Hyperbolar zones (or homologous vanes) = present (1)
    - Discussion: Added coding for *Proteroctopus*.

28. Wing length / rachis length: < 0.3 (0); ≥ 0.3(1); ≥ 0.5 (2); ≥ 0.7 (3); ≥ 0.9 (4) [ordered character]
    - Sutton et al. (2016) #26
    - Dependency: Median field (or homologous rachis) = present (1)
    - Dependency: Lateral fields (or homologous wings) = present (1)
    - Discussion: Added coding for *Hibolithes*. *Dorateuthis* corrected from 0 to 2 (=0.69), and *Senefelderiteuthis* corrected from 1 to 3 (=0.79). Added coding for *Proteroctopus*.

29. Cone flags length / gladius length: < 0.3 (0); ≥ 0.3 (1)
    - Sutton et al. (2016) #29
    - Dependency: 'Cone flags' = present (1)
    - Discussion: Former state "< 0.1" removed because it does not apply to any included taxa. *Vampyroteuthis infernalis* corrected from 1 to 0.

30. Vane width / rachis width at vane: < 0.25 (0); < 0.75 (1); < 1.25 (2); < 2.5 (3); < 4 (4); ≥ 4 (5) [ordered character]
    - Sutton et al. (2016) #34
    - Dependency: Median field (or homologous rachis) = present (1)
    - Dependency: Hyperbolar zones (or homologous vanes) = present (1)
    - Discussion: Recoded from "?"in *Proteroctopus.*

31. Wing width / rachis width at wing: < 0.25 (0); < 0.75 (1); < 1.25 (2); ≥ 2.5 (3) [ordered character]
    - Sutton et al. (2016) #35
    - Dependency: Median field (or homologous rachis) = present (1)
    - Dependency: Lateral fields (or homologous wings) = present (1)
    - Discussion: Only one taxon was coded as former state 3 (< 2.5) and former state 4 (< 4); no taxa were coded as former state 5 (≥ 4). So, 3-5 were combined into one state here. Recoded from "?"in *Proteroctopus, Dorateuthis, Senefelderiteuthis,* and *Hibolithes*. *Boreopeltis smithi* (=0.13) and *Plesioteuthis prisca* (=0.06) corrected from 1 to 0. *Loligosepia aalensis* (=0.32) corrected from 0 to 1.

32. Position of greatest width of median field / rachis: at extreme anterior (0); posterior to extreme anterior (1)
    - Sutton et al. (2016) #38
    - Dependency: Median field (or homologous rachis) = present (1)

- Discussion: Character polarity reversed since the widest portion of the ancestral median field is the adapical portion of the cone. Also, this was conceived as an ordered character (here a contingent character), but the first two states in the list (at hyperbolar zone / vane insertion; anterior to hyperbolar zone / vane insertion but not at extreme anterior) depend on the presence of a hyperbolar zone, which is irrelevant to determining whether the extreme anterior is the widest portion. Recoded as “0” from “?” for *Vampyronassa*, *Proteroctopus*, and *Hibolithes*.

33. Position of greatest width of median field / rachis: anterior to hyperbolar zone / vane insertion but not at extreme anterior (0); at hyperbolar zone / vane insertion (1)
    - Sutton et al. (2016) #38
    - Dependency: Position of greatest width of median field / rachis = posterior to extreme anterior (1)
    - Dependency: Hyperbolar zones (or homologous vanes) = present (1)
    - Discussion: Added coding for *Sepia.*

34. Rachis width at vane insertion / rachis width 2/3 of the way between vane insertion and the anterior: ≤ 1 (0); ≤ 1.5 (1); ≤ 2.5 (2); > 2.5 (3) [ordered character]
    - Sutton et al. (2016) #39
    - Dependency: Median field (or homologous rachis) = present (1)
    - Dependency: Hyperbolar zones (or homologous vanes) = present (1)
    - Discussion: Added coding for *Sepia*.

35. Concave inflexion or inflexions in median field / rachis: absent (0); present (1)
    - Sutton et al. (2016) #40
    - Dependency: Median field (or homologous rachis) = present (1)

36. Outline of posterior margin of lateral field / wings: convex (0); straight (1); concave (2)
    - Sutton et al. (2016) #41
    - Dependency: Lateral fields (or homologous wings) = present (1)
    - Discussion: Added coding for *Dorateuthis* and *Hibolithes*.

37. Shape of posterior end of median field / rachis: concave (0), flat (1), convex (2)
    - Sutton et al. (2016) #42
    - Dependency: Median field (or homologous rachis) = present (1)
    - Discussion: Recoded from “?”in *Proteroctopus* and *Hibolithes*

38. Nature of convex end: rounded (0); pointed (1)
    - Sutton et al. (2016) #43
    - Dependency: Shape of posterior end of median field / rachis = convex (2)
    - Discussion: Recoded from “?”in *Proteroctopus* and *Hibolithes*

39. Shape of anterior tip of median field: concave (0), flat (1), convex (2)
    - Sutton et al. (2016) #44
    - Dependency: Median field (or homologous rachis) = present (1)

- Discussion: Clarified original character to more clearly indicate that it refers to the median field. Added coding for *Hibolithes.*

40. Nature of convex tip: rounded (0); pointed (1)
    - Sutton et al. (2016) #45
    - Dependency: Shape of anterior tip of gladius = convex (2)

41. Inflexion in gladius outline where lateral asymptote intersects margin: absent (0); present (1)
    - Sutton et al. (2016) #46
    - Dependency: Hyperbolar zones (or homologous vanes) = present (1)

42. Inflexion in gladius outline where lateral asymptote intersects margin: weak (0); strong/sharp (1)
    - Sutton et al. (2016) #46
    - Dependency: Inflexion in gladius outline where lateral asymptote intersects margin = present (1)

43. Tapering of the hyperbolar zone / vane both anteriorly and posteriorly (forming a spindle shape): absent (0); present (1)
    - Sutton et al. (2016) #47
    - Dependency: Hyperbolar zones (or homologous vanes) = present (1)
    - Discussion: Coded for *Hibolithes.*

44. Angle between inner/median asymptote and midline: ≤ 5 degrees (0); > 5 and ≤ 10 degrees (1); > 10 and ≤ 15 degrees (2); > 15 and ≤ 20 degrees (3); > 20 degrees (4) [ordered character]
    - Sutton et al. (2016) #48
    - Dependency: Median field (or homologous rachis) = present (1)
    - Discussion: This character is intended to measure an aspect of the median field, thus in taxa without hyperbolar zones, the angle is between the midline and the boundary between the median field and the lateral fields; in taxa without hyperbolar zones and lateral fields, the angle is measured between the midline and the outer (lateral) edge of the gladius/proostracum. Recoded from "?"in *Proteroctopus* and *Hibolithes. Senefelderiteuthis* (=5.87°) and *Dorateuthis* (=5.14°) corrected from 0 to 1

45. Angle between outer/lateral asymptote and midline: ≤ 5 degrees (0); > 5 and ≤ 10 degrees (1); > 10 and ≤ 15 degrees (2); > 15 and ≤ 20 degrees (3); > 20 degrees (4) [ordered character]
    - Sutton et al. (2016) #49
    - Dependency: Median field (or homologous rachis) = present (1)
    - Dependency: Hyperbolar zones (or homologous vanes) = present (1)
    - Dependency: Lateral fields (or homologous wings) = present (1)

- Discussion: Recoded as inapplicable in Prototeuthidina; added coding for *Proteroctopus*.

46. Ventral median field / rachis in transverse section: smooth (0); interrupted by a median or sub-median structure or structures (‘interruption(s)’) (1)
    - Sutton et al. (2016) #51
    - Dependency: Median field (or homologous rachis) = present (1)
    - Discussion: Recoded as “0” from “?” in *Vampyronassa, Hibolithes, Phragmoteuthis,* and *Belemnotheutis*.

47. Form of ventral interruption(s): concave (0); convex (1)
    - Sutton et al. (2016) #52
    - Dependency: Ventral median field / rachis in transverse section = interrupted by a median or sub-median structure or structures (‘interruption(s)’) (1)

48. Form of ventral interruption(s): line (0); rib/keel (1)
    - Sutton et al. (2016) #52
    - Dependency: Form of ventral interruption(s) = convex (1)

49. Form of ventral interruption(s): rib (0); keel (1)
    - Sutton et al. (2016) #52
    - Dependency: Form of ventral interruption(s) = rib/keel (1)

50. Anterior ventral interruption(s): absent (0); present (1)
    - Sutton et al. (2016) #53
    - Dependency: Ventral median field / rachis in transverse section = interrupted by a median or sub-median structure or structures (‘interruption(s)’) (1)

51. Posterior ventral interruption(s): absent (0); present (1)
    - Sutton et al. (2016) #53
    - Dependency: Ventral median field / rachis in transverse section = interrupted by a median or sub-median structure or structures (‘interruption(s)’) (1)

52. Ventral interruption bipartite (at any point anterior-posterior)?: no (0); yes (1)
    - Sutton et al. (2016) #54
    - Dependency: Ventral median field / rachis in transverse section = interrupted by a median or sub-median structure or structures (‘interruption(s)’) (1)

53. Dorsal median field / rachis in transverse section: smooth (0); interrupted by a median or sub-median structure or structures (‘interruption(s)’) (1)
    - Sutton et al. (2016) #55
    - Dependency: Median field (or homologous rachis) = present (1)
    - Discussion: Coded as “0” from “?” in *Vampyronassa*, *Hibolithes, Phragmoteuthis,* and *Belemnotheutis*. *Boreopeltis smithi* corrected from absent to present.

54. Form of dorsal interruption(s): concave (0); convex (1)
    - Sutton et al. (2016) #56
    - Dependency: Dorsal median field / rachis in transverse section = interrupted by a median or sub-median structure or structures (‘interruption(s)’) (1)

55. Form of dorsal interruption(s): line (0); rib/keel (1)
    - Sutton et al. (2016) #56
    - Dependency: Form of dorsal interruption(s): convex (1)

56. Form of dorsal interruption(s): rib (0); keel (1)
    - Sutton et al. (2016) #56
    - Dependency: Form of dorsal interruption(s): rib/keel (1)

57. Dorsal interruption(s) at 25% of rachis length from anterior: not present (0); present (1)
    - Sutton et al. (2016) #57
    - Dependency: Dorsal median field / rachis in transverse section = interrupted by a median or sub-median structure or structures (‘interruption(s)’) (1)

58. Dorsal interruption(s) at 25% of rachis length from anterior: one structure, not split (0); split in two structures (1)
    - Sutton et al. (2016) #57
    - Dependency: Dorsal interruption(s) at 25% of rachis length from anterior = present (1)
    - Discussion: No taxa were coded for the former state “split in three structures” (3), so it was removed. *Boreopeltis sagittate* corrected from 1 to 0.

59. Dorsal interruption(s) at 75% length of rachis length from anterior: not present (0); present (1)
    - Sutton et al. (2016) #58
    - Dependency: Dorsal median field / rachis in transverse section = interrupted by a median or sub-median structure or structures (‘interruption(s)’) (1)
    - Discussion: Recoded as present from “?”in *Proteroctopus* and *Boreopeltis.*

60. Dorsal interruption(s) at 75% length of rachis length from anterior: one structure, not split (0); split in two structures (1)
    - Sutton et al. (2016) #58
    - Dependency: Dorsal interruption(s) at 75% length of rachis length from anterior = present (1)
    - Discussion: No taxa were coded for the former state “split in three structures” (3), so it was removed. Recoded from unknown to “1” (split into two structures) in *Proteroctopus* and to “0” in *Boreopeltis.*

61. Lateral reinforcements on the median field / rachis: absent (0); present (1)
    - Sutton et al. (2016) #59

- Dependency: Median field (or homologous rachis) = present (1)
- Discussion: Added coding for belemnoids, *Vampyronassa*, and *Proteroctopus*. Corrected *Glyphiteuthis freijii* to absent (was present).

62. Extent of lateral reinforcements: anterior (0); majority/whole of the median field / rachis (1)
    - Sutton et al. (2016) #60
    - Dependency: Lateral reinforcements on the median field / rachis = present (1)
    - Discussion: No taxa were coded as posterior (former state 1), so it was removed.

63. Lateral reinforcement position: not at edge of median field (0); at edge of median field (1)
    - Sutton et al. (2016) #61
    - Dependency: Lateral reinforcements on the median field / rachis = present (1)

64. Lateral reinforcements subdivided: no (0); yes (1)
    - Sutton et al. (2016) #62
    - Dependency: Lateral reinforcements on the median field / rachis = present (1)

65. Thin lateral plates on the dorsal median field/rachis: absent (0); present (1)
    - Sutton et al. (2016) #63
    - Dependency: Median field (or homologous rachis) = present (1)
    - Discussion: Recoded belemnoids, *Vampyronassa*, *Palaeololigo*, and *Proteroctopus* as "0" from "?"

66. Granules (tubercles) on the gladius/proostracum: absent (0); present (1)
    - Sutton et al. (2016) #64
    - Dependency: Discrete proostracum developed: yes (1)
    - Discussion: Wording changed from original "on the dorsal surface of the shell" because cuttlefish have a granulated rostrum, while all other included taxa have a granulated proostracum/gladius. These are not homologous conditions.

67. Arrangement of granules in the anterior half: regular (0); irregular (1)
    - Sutton et al. (2016) #65
    - Dependency: Granules (tubercles) on the dorsal surface of the gladius/proostracum = present (1)

68. Arrangement of granules in the posterior half: regular (0); irregular (1)
    - Sutton et al. (2016) #66
    - Dependency: Granules (tubercles) on the dorsal surface of the gladius/proostracum = present (1)

69. Granule size-change from posterior to anterior: decreasing (0); no change (1); increasing (2) [ordered character]
    - Sutton et al. (2016) #67

- Dependency: Granules (tubercles) on the dorsal surface of the gladius/proostracum = present (1)

70. Granule size-change from midline towards lateral margin: decreasing (0); no change (1); increasing (2) [ordered character]
    - Sutton et al. (2016) #68
    - Dependency: Granules (tubercles) on the dorsal surface of the gladius/proostracum = present (1)

71. Row of relatively coarse granules along lateral edges of granulated area: absent (0); present (1)
    - Sutton et al. (2016) #69
    - Dependency: Granules (tubercles) on the dorsal surface of the gladius/proostracum = present (1)

72. Row of relatively coarse granules along median field / rachis: absent (0); present (1)
    - Sutton et al. (2016) #70
    - Dependency: Granules (tubercles) on the dorsal surface of the gladius/proostracum = present (1)

73. Lateral margins of the granulated area meet anteriorly at the anterior edge of the median field / rachis?: no (0); yes (1)
    - Sutton et al. (2016) #71
    - Dependency: Granules (tubercles) on the dorsal surface of the gladius/proostracum = present (1)

74. Lateral margins of posterior 1/3 of granulated area: straight (0); curved (1)
    - Sutton et al. (2016) #72
    - Dependency: Granules (tubercles) on the dorsal surface of the gladius/proostracum = present (1)

75. Width of granulated area at vane / rachis width at vane: < 0.5 (0); ≥ 0.5 (1)
    - Sutton et al. (2016) #73
    - Dependency: Granules (tubercles) on the dorsal surface of the gladius/proostracum = present (1)

76. Fins: absent (0); present (1)
    - Sutton et al. (2016) #74
    - Discussion: Recoded from unknown to absent in *Keuppia levante.*

77. Number of fin pairs: 1 pair (0); 2 pairs (1)
    - Sutton et al. (2016) #75
    - Dependency: Fins = present (1)

78. Shape of fins: lobate (0); rhomboidal (1); skirt-like (2)

- Sutton et al. (2016) #76
- Dependency: Fins = present (1)
- Discussion: Skirt-like condition added to account for sepiids, which cannot be considered to have lobate fins, even with a broad definition of the term. *Muensterella* also has a sepiid-like skirt-like fin; not a chtenopterygid fringing fin, which is unique to chtenopterygids.

79. Posterior fin termination: subterminal (0); terminal (1)
    - Sutton et al. (2016) #78
    - Dependency: Fins = present (1)

80. Maximum length of unmodified arms compared to mantle length: shorter (0); longer or approximately equivalent (1)
    - Sutton et al. (2016) #79
    - Dependency: Discrete proostracum developed = yes (1)
    - Discussion: The presence of a proostracum is irrelevant to this character, which is principally concerned with the length of the arms. Therefore, we reworded it from "Maximum length of unmodified arms compared to gladius length." Added coding for *Heteroteuthis, Spirula*, *Amphitretus*, *Argonauta*, *Japetella, Ocythoe, Trachyteuthis hastiformis, Proteroctopus* and *Vampyronassa*. *Senefelderiteuthis* recoded from 1 to 0 (Fuchs, 2020). *Architeuthis, Abralia, Gonatus, Lepidoteuthis, Cycloteuthis*, *and Glyphiteuthis freijii* changed from 0 to 1 (Jereb & Roper, 2010).

81. Maximum length of unmodified arms compared to mantle length: approximately the same, ±10% (0); longer (1)
    - Sutton et al. (2016) #79
    - Dependency: Maximum length of unmodified arms compared to mantle length = longer or approximately equivalent (1)
    - Discussion: *Architeuthis* and *Abralia* changed from inapplicable to 1. *Lepidoteuthis, Gonatus,* and *Cycloteuthis* changed from inapplicable to 0.

82. Number of appendage pairs: many (0); 4-5 (1)
    - Sutton et al. (2016) #80
    - Discussion: Distinguishing 4 arm pairs from 5 arm pairs here (as in Sutton et al., 2016) is redundant with the following two characters, which specifically reference the loss of arm pair II in octobrachians. Added coding for *Loligosepia aalensis* (Fuchs et al., 2013).

83. Reduction of appendage pair II: not reduced (0), reduced to filaments or absent (1)
    - Sutton et al. (2016) #81
    - Dependency: Modification of appendage pair II = modified (1)
    - Discussion: Added coding for *Loligosepia aalensis* (Fuchs et al., 2013), *Senefelderiteuthis, Palaeoctopus, Keuppia levante, Glyphiteuthis freijii, Dorateuthis*, *Rachiteuthis*, *Leptoteuthis gigas* (Fuchs, 2020), and *Jeletzkyteuthis* (Klug et al., 2021).

84. Loss of appendage pair II: retained (0); lost (1)
- New Character
- Dependency: Reduction of appendage II = reduced to filaments or absent (1)
- Discussion: Added coding for *Loligosepia aalensis* (Fuchs et al., 2013), *Senefelderiteuthis*, *Palaeoctopus*, *Keuppia levante*, *Glyphiteuthis freijii*, *Dorateuthis*, *Rachiteuthis*, and *Leptoteuthis gigas* (Fuchs, 2020).

85. Modification of appendage IV into a tentacle: absent (0); present (1)
- Sutton et al. (2016) #82
- Dependency: Number of appendage pairs = 4-5 (1)
- Discussion: *Lepidoteuthis* and *Octopoteuthis* have tentacles as paralarvae, so they should be coded as present. If they are coded as absent because the tentacles are lost in adulthood, the implication is that they are plesiomorphic for this character and thus strong candidates for being the basalmost decabrachians, which they are not. Rather this secondary loss is either an autapomorphy of each genus or a synapomorphy uniting them. A new character has been added to account for the possibly synapomorphic nature of this character. Added coding for *Loligosepia aalensis* (Fuchs et al., 2013), *Muensterella*, and *Glyphiteuthis freijii* (Fuchs, 2020).

86. Loss of tentacles in adulthood: no (0); yes (1)
- New Character
- Dependency: Modification of appendage IV into a tentacle = present (1)
- Discussion: Only present in *Lepidoteuthis* and *Octopoteuthis*

87. Retractile tentacles: absent (0); present (1)
- New Character
- Dependency: Modification of appendage IV into a tentacle = present (1)
- Discussion: Characteristic of sepiids, sepiolids, spirulids, and idiosepiids (Jereb & Roper, 2005)

88. Tentacle suckers: in up to 4 rows (0); in more than 4 rows (1)
- Sutton et al. (2016) #83
- Dependency: Modification of appendage IV into a tentacle = present (1)
- Dependency: Suckers on any appendage: present (1)

89. Tentacle locking apparatus: absent (0); present (1)
- Sutton et al. (2016) #84
- Dependency: Modification of appendage IV into a tentacle = present (1)

90. Tentacle locking apparatus: present on carpus only (0); present on manus and carpus (1)
- Sutton et al. (2016) #84
- Dependency: Tentacle locking apparatus = present (1)

91. Interbrachial web: absent (0); present (1)
    - Sutton et al. (2016) #85
    - Discussion: Recoded from unknown to absent in *Keuppia levante*.

92. True arm hooks: absent (0); present (1)
    - Sutton et al. (2016) #86
    - Discussion: The hooks of extant oegopsids are modified suckers – the gradation from a sucker to a hook can be observed within individual appendages (Kulicki & Szaniawski, 1972; Jereb & Roper, 2010). Contrarily, the hooks of belemnoids exist alongside and independent of their suckers (Fuchs et al., 2010). Although in theory it could be difficult to distinguish these hook types, in practice it is straightforward. Hooks are only known from belemnoids, oegopsids, and scaphitid ammonoids (Kruta et al., 2019), and these taxa are very easily distinguished. Hooks have been reported from the loligosepiid *Loligosepia aalensis* (Doguzhaeva & Mutvei, 1991), but for several reasons we consider this highly unlikely:
        i. Hooks have not been identified in any other specimens of *Loligosepia aalensis,* any other *Loligosepia* species, any other members of Loligosepiina, any other octobrachians, or indeed any other non-belemnoid fossil coleoids. Hooks tend to be rather robust taphonomically; many gladius bearing fossils preserve arms with the much more delicate suckers or cirri, but no hooks. Therefore, the absence of hooks in these fossils is likely a true absence.
        ii. The hooks and associated appendage appear to be orders of magnitude too small to belong to the associated specimen.
        iii. The appendage is preserved as a few isolated fragments. If taphonomic conditions allowed part of one appendage to be preserved, one would expect more appendage fragments, additional hooks, or some remains of the head.
        iv. The hooks and appendage are preserved beneath the gladius, not anterior to it.

      Given all this, we consider it far more likely that the appendage fragment and hooks are prey remnants, likely from a small belemnoid, preserved within the crop or stomach of the larger (hook-free) loligosepiid. Contra Sutton et al. (2016) and Kruta et al. (2016), we code *Loligosepia aalensis* as absent for both hook characters.

93. Cirri or spines on arms: absent (0): present (1)
    - Sutton et al. (2016) #87
    - Discussion: Recoded as absent from “?” for *Belemnotheutis*.

94. Suckers on any appendage: absent (0); present (1)
    - Sutton et al. (2016) #88

95. Sucker-hooks: absent (0); present (1)
    - New Character

- Dependency: Suckers on any appendage = present (1)
- Discussion: Suckers that have been secondarily modified into hooks; observed in extant decabrachians. See discussion of the character “true arm hooks”

96. Sucker symmetry: radial (0); bilateral (1)
    - Sutton et al. (2016) #89
    - Dependency: Suckers on any appendage = present (1)
    - Discussion: Recoded as radial from unknown in *Belemnotheutis*.

97. Sucker-stalk on arms: absent (sessile, unstalked) (0); present (1)
    - Sutton et al. (2016) #90
    - Dependency: Suckers on any appendage = present (1)

98. Sucker-stalk on arms: attached to arm muscles (0); not clearly attached to arm muscles (1)
    - Sutton et al. (2016) #90
    - Dependency: Sucker-stalk on arms = present (1)

99. Shape of stalks attached to the arm muscles: conical pillar with base and neck (0); cylinder (1)
    - Sutton et al. (2016) #91
    - Dependency: Suckers on any appendage = present (1)

100. Lining of arm suckers: neither horny nor cuticular ring (0); horny ring (1); cuticular ring (2)
    - Sutton et al. (2016) #92
    - Dependency: Suckers on any appendage = present (1)

101. Suckers (on arms) proximally: absent (0); present (1)
    - Sutton et al. (2016) #93
    - Dependency: Suckers on any appendage = present (1)

102. Suckers (on arms) proximally: 1 row (0); > 1 rows (1)
    - Sutton et al. (2016) #93
    - Dependency: Suckers (on arms) proximally = present (1)

103. Suckers (on arms) proximally: 2 rows (0); > 2 rows (1)
    - Sutton et al. (2016) #93
    - Dependency: Suckers (on arms) proximally: > 1 rows (1)

104. Suckers (on arms) medially: absent (0); present (1)
    - Sutton et al. (2016) #94
    - Dependency: Suckers on any appendage = present (1)

105. Suckers (on arms) medially: 1 row (0); > 1 rows (1)
    - Sutton et al. (2016) #94

- Dependency: Suckers (on arms) medially = present (1)

106. Suckers (on arms) medially: 2 rows (0); > 2 rows (1)
    - Sutton et al. (2016) #94
    - Dependency: Suckers (on arms) medially = > 1 rows (1)

107. Suckers (on arms) distally: absent (0); present (1)
    - Sutton et al. (2016) #95
    - Dependency: Suckers on any appendage = present (1)

108. Suckers (on arms) distally: 1 row (0); > 1 rows (1)
    - Sutton et al. (2016) #95
    - Dependency: Suckers (on arms) distally = > 0 row (1)

109. Suckers (on arms) distally: 2 rows (0); > 2 rows (1)
    - Sutton et al. (2016) #95
    - Dependency: Suckers (on arms) distally = > 1 rows (1)

110. Ink sac: absent (0); present (1)
    - Sutton et al. (2016) #96

111. Nuchal cartilage: absent (0); present (1)
    - Sutton et al. (2016) #97
    - Discussion: *Idiosepius* was incorrectly coded as present; changed to absent (Jereb & Roper, 2005)

112. Chromatophores: (0) absent; (1) present
    - Sutton et al. (2016) #98

113. Buccal crown: (0) absent; (1) present
    - Sutton et al. (2016) #99
    - Discussion: The decabrachian buccal crown is considered homologous to the inner arm crown of nautilids (Berthold & Engeser, 1987); so, we recode this as present in *Nautilus*.

114. Buccal membrane connective attachment to arm-pair V: dorsal (0); ventral (1)
    - Sutton et al. (2016) #100
    - Dependency: Buccal crown = (1) present

115. Number of buccal lappets (supports): 6 (0); 7 (1); 8 (2); Many (3)
    - Sutton et al. (2016) #101
    - Dependency: Buccal crown = (1) present
    - Discussion: State 3 added for the inner arms of nautilids

116. Suckers on buccal membrane: absent (0); present (1)

- Sutton et al. (2016) #102
- Dependency: Buccal crown = (1) present
- Dependency: Suckers on any appendage = present (1)

117. Hydrostatic organ (swim-bladder): absent (0); present (1)
    - Sutton et al. (2016) #103

118. Calcareous elements in the beak: absent (0); present (1)
    - Sutton et al. (2016) #104
    - Discussion: Added codings of "absent" for several taxa

119. Radula: absent (0); present (1)
    - Sutton et al. (2016) #105

120. Horizontal arm septa inserted in the arm muscles: absent (0); present (1)
    - Sutton et al. (2016) #106

121. Luminous autogenic organs: absent (0); present (1)
    - Sutton et al. (2016) #107

122. Photophores containing collagen light guides: absent (0); present (1)
    - Sutton et al. (2016) #108

123. Funnel connection to mantle: attached to ventral mantle (0); free, not attached to ventral mantle (1); fused to ventral mantle (2)
    - Sutton et al. (2016) #109

124. Funnel locking apparatus: absent (0); present (1)
    - Sutton et al. (2016) #110

125. Funnel locking apparatus morphology: simple, straight (0); triangular, round (1); inverted T or-|shaped (2); oval with projecting knobs (3)
    - Sutton et al. (2016) #111
    - Dependency: Funnel locking apparatus = present (1)

126. Funnel valve: absent (0); present (1)
    - Sutton et al. (2016) #112

127. Eye shape: hemispherical (0); other shapes (1)
    - Sutton et al. (2016) #113

128. Asymmetrical eyes, with one significantly enlarged: absent (0); present (1)
    - New Character
    - Discussion: Defining characteristic of Histioteuthidae

129. Cornea: absent (0); one-part decabrachian-type cornea present (1); two-part incirrate-like cornea present (2)
    - Sutton et al. (2016) #114
    - Discussion: *Cirrothauma* recoded from 2 (two-part) to 0 (absent); since the text of Sutton et al. (2014) seems to indicate absence of this character, this coding appears to have been a simple data entry error

130. Statocyst outer capsule: absent (0); present (1)
    - Sutton et al. (2016) #115

131. Photosensitive vesicles: within cephalic cartilage (0); above funnel (1); on stellate ganglia (2)
    - Sutton et al. (2016) #116

132. Inferior frontal lobe system of the brain: absent (0); present (1)
    - Sutton et al. (2016) #117

133. Inferior frontal lobe system of the brain: partially present (0); fully present (1)
    - Sutton et al. (2016) #117
    - Dependency: Inferior frontal lobe system of the brain = present (1)

134. Superior buccal lobe: widely separated from brain (0); adjacent/fused to brain (1)
    - Sutton et al. (2016) #118

135. Superior buccal lobe: adjacent to brain (0); fused to brain (1)
    - Sutton et al. (2016) #118
    - Dependency: Superior buccal lobe = adjacent/fused to brain (1)

136. Relative position of digestive gland duct appendages (DGDA) and the nephridial coelom: lies in nephridial coelom (0); not in nephridial coelom (1)
    - Sutton et al. (2016) #119

137. Posterior salivary gland: absent (0); present (1)
    - Sutton et al. (2016) #120

138. Posterior salivary gland position: posterior to brain (0); proximal to buccal mass (1)
    - Sutton et al. (2016) #121
    - Dependency: Posterior salivary gland = present (1)

139. Branchial canal: absent (0); present; (1)
    - Sutton et al. (2016) #122

140. Gill-lamellae attachment: free (0); sessile (1)
    - Sutton et al. (2016) #123

141. Nidamental glands: (0) absent; (1) present
    - Sutton et al. (2016) #124

142. Accessory nidamental glands: (0) absent; (1) present
    - New Character
    - Discussion: Present in all decabrachians except for oegopsids (Berthold & Engeser, 1987)

143. Right oviduct: absent (0); present (1)
    - Sutton et al. (2016) #125

144. Oviducal gland symmetry: radial (0); bilateral (1); asymmetrical (2)
    - Sutton et al. (2016) #126

145. Oviducal gland position: gland terminal (located at end of oviduct) (0); gland subterminal (1)
    - Sutton et al. (2016) #127

146. Arm-pair I hectocotylization or other sexual modifications: absent (0); present (1)
    - Sutton et al. (2016) #128
    - Dependency: Number of appendage pairs = 4-5 (1)

147. Arm-pair IV hectocotylization or other sexual modifications: absent (0); present (1)
    - Sutton et al. (2016) #129
    - Dependency: Number of appendage pairs = 4-5 (1)

148. Arm-pair V hectocotylization or other sexual modifications: absent (0); present (1)
    - Sutton et al. (2016) #130
    - Dependency: Number of appendage pairs = 4-5 (1)

149. Spermatophore type: with ejaculatory mechanism (0); sperm-packets as in cirrate octopods (1); with encapsulated coil (2)
    - Sutton et al. (2016) #131

150. Dorsal mantle cavity: absent (0); present (1)
    - New Character
    - Discussion: Unique to octopods (Young & Vecchione, 1996)

151. Collagenous tunic on mantle: absent (0); present (1)
    - New Character
    - Discussion: Unique to decabrachians (Young & Vecchione, 1996)

152. Stellar ganglia connected by a commissure: absent (0); present (1)
    - New Character
    - Discussion: Appears to be unique to oegopsids (Berthold & Engeser, 1987)

153. Development of a commissure between the mantle and visceral nerve: absent (0); present (1)
    - New Character
    - Discussion: Appears to be unique to oegopsids (Berthold & Engeser, 1987)

The following characters from Sutton et al. (2016) and Kruta et al. (2016) have been removed from the phylogenetic analysis; justifications for removal are provided.

1. Proostracum: one unit (0); two units (1)
    - Sutton et al. (2016) #10
    - Justification: This is a consequence of the presence of a patella but the absence of the conus and median field; all of which are independent characters.

2. Phragmocone wall (conotheca) thickened: no (0); yes (1)
    - Sutton et al. (2016) #21
    - Justification: It is unclear what this character refers to; the presence of a rostrum and primordial rostrum, as well as shell mineralization, are handled by separate characters.

3. Phragmocone (primary cone) length / gladius length: < 0.02 (0); < 0.1 (1); ≥ 0.1 (2); ≥ 0.3(3); ≥ 0.5 (4) [ordered character]
    - Sutton et al. (2016) #27
    - Justification: This character is difficult to measure. Since the ventral surface of the conus is rarely flat, a fair comparison would record the length of a median spline (not a line) on the ventral surface of the conus, whether the surface is oriented anteriorly ("funnel-like" and "cup-like"), ventrally ("open ventrally"), or posteriorly ("patella"). This character cannot simply be measured as the anteriormost extent of the conus, as that value changes based on the angle and shape of the conus apex, independent of the length of the ventral surface of the conus.

4. Secondary cone length / gladius length: < 0.1 (0); < 0.2 (1); ≥ 0.2 (2) [ordered character]
    - Sutton et al. (2016) #28
    - Justification: This could only be coded for three taxa, two of which were coded as zero.

5. Rachis width at vane / total wing width: < 0.25 (0); < 0.5 (1); < 0.75 (2); ≥ 0.75 (3) [ordered character]
    - Sutton et al. (2016) #30
    - Justification: This character is unnecessarily restrictive as defined. It describes the median field through reference to the lateral field, but it requires the presence of hyperbolar zones, which are otherwise irrelevant to the metric. This forces many taxa to be coded as inapplicable, not because the metric cannot exist, but because we cannot determine where the median field should be measured. Also, the information contained within this character overlaps with the information contained in the characters "Position of greatest width of median field / rachis" and "Wing width / rachis width at wing."

6. Wing length / vane length: < 0.9 (0); 0.9 - 1.1 (1); > 1.1 (2) [ordered character]
    - Sutton et al. (2016) #31

- Justification: The hyperbolar zone length and lateral field length have both already been measured via comparison to the median field length. This character is thus redundant. The few taxa lacking median fields as a reference do not justify duplicating this information in all other taxa.

7. Vane width / wing width: < 0.25 (0); < 0.5 (1); < 0.75 (2); < 1 (3) ≥ 1 (4) [ordered character]
    - Sutton et al. (2016) #32
    - Justification: The hyperbolar zone width and lateral field width have both already been measured via comparison to the median field width. This character is thus redundant. The few taxa lacking median fields as a reference do not justify duplicating this information in all other taxa.

8. Vane width / rachis width at wing: < 0.25 (0); < 0.75 (1); < 1.25 (2); < 2.5 (3); < 4 (4); ≥ 4 (5) [ordered character]
    - Sutton et al. (2016) #33
    - Justification: This character is unnecessarily restrictive as defined. It describes the hyperbolar zones through reference to the median field, but it requires the presence of lateral fields, which are otherwise irrelevant to the metric. This forces taxa to be coded as inapplicable, not because the metric cannot exist, but because we cannot determine where the median field should be measured. Also, the information contained within this character overlaps with the character "Vane width / rachis width at vane."

9. Constriction of the lateral fields / wings: absent (0); present (1)
    - Sutton et al. (2016) #36
    - Justification: The "constriction" reported for octopod stylets may be better understood as the narrowing of the gladius patella towards the apex. This would not be immediately obvious because octopods have lost the median field and the conus. Under this scenario, the morphology described by this character would be a direct result of the presence of lateral fields and a patella, along with the loss of the median field and conus. Therefore, conservatively, we decided not to code it as a distinct character. If the character is retained, it would only apply to stylet-bearing octopods, so removing it has little impact on the phylogeny.

10. Constriction of the lateral fields / wings: posterior (0); medial (1)
    - Sutton et al. (2016) #37
    - Justification: See 9

11. Median asymptotes posterior intersection: do not intersect (if extended would intersect posterior to gladius) (0); intersect at or very near to the posterior margin of the gladius (1); intersect within gladius (2) [ordered character]
    - Sutton et al. (2016) #50
    - Justification: This character captures the presence/absence of a patella, which is already accounted for

12. Single fin condition: discrete (0); fringing (1)
    - Sutton et al. (2016) #77
    - Justification: This character is an autapomorphy of *Chtenopteryx*. *Muensterella* has a skirt-like fin (as in sepiids), not a *Chtenopteryx*-like fringing fin (contra Sutton et al., 2014).

13. Decalcified septa: absent (0); present (1)
    - Sutton et al. (2016) #18
    - Justification: redundant with character “mineralized phragmocone” since no taxa have a mineralized conotheca but demineralized septa and vice versa.

14. Proostracum U-shaped: no (0); yes (1)
    - Sutton et al. (2016) #6
    - Justification: This character is a consequence of the presence of hyperbolar zones, lateral fields, and a conus, alongside the absence of a median field.

Character-taxon matrix:

Nautilus_pompilius 10--0-1111--------2--0-0-
0-0--------0000---------------0001-3-01100000-10000---0--0-0010120--020000

Hibolithes_semisulcatus 112111111?1011111100--00-0-4--00--?0011-??0?-0------0-------0---
00---------
????111?????????1??0??????????????1???????0????????????????????????????????????

Phragmoteuthis_bisinuata 112111111011111?0100--?0-?33?120-000??20110220------0-------
0---00---------
????????????????1?????????????????1???

Bathyteuthis_abyssicola 1120110---11010-00-??000-42--5-0-00-2121??00-10--1001110100-
101000---------10000-10-10010-10010110011101111111110110010001101100000-0-
0101010110000001??

Chtenopteryx_sicula 1120110---1100--00-??010-42-03-1110-2121??10-
1110110111110100---00---------10010-10-10010-
000101100111011011111111110010001101000000-0-0101010110000001??

Idiosepius_pygmaeus 1110110---1100--00-??000-1?--?-10??-2020??0?-111010110--110-
0---00---------10000-10-10100-10010110011101101101010--00010001131001000-0-
010101111000100???

Doryteuthis_amerigo_pealeii 1120110---1100--00-??000-43--2-1110-2121??11-10--010110-0-
100---00---------10110-10-10000-00010110011101101101111110110001101001000-0-
010101101000100100

Abralia_trigonura 1120110---1100--01-10-00-34--3-1110-2121??11-10--110111010100-
--00---------10110-10-1000100001111001110110110111102000100111?1000000-0-
010101011000100111

Architeuthis_dux 1120110---1100--01-10-10-42-?2-1110-2121??11-1110111111010100-
--00---------10010-10-100011000101100111011011011110100010001101000000-0-
010101011000100111

Cranchia_scabra 1120110---1100--01-??001151--3-1000-2121??00-10--01011110-
100---00---------10010-10-10000-000101100111011011010111100010002??1000000-0-
010101011000100111

Cycloteuthis_sirventi 1120110---1100--00-??001153--2-1020-2121??11-10--01011100-
100---00---------10000-10-100011000101100111011011011111100010001111000000-0-
010101011000000111

Gonatus_antarcticus 112011101011010001010-10-53-03-1000-2120??10-0------0-------
111000---------10110-10-10011100011110011111111111111110001000110100000-0-
010101011000100111

Histioteuthis_celetaria 1120110---1100--00-??000-33--4-1100-2121??00-10--010110-0-
10101000---------10011010-1001111001011001?????????1111010001010110111000-0-
010101011010000111

Histioteuthis_corona 1120110---1100--00-??000-43--3-1110-2121??10-10--010110-0-
10101000---------10011110-10011110010110011101101101011010001010110111000-0-
010101011010000111

Lepidoteuthis_grimaldii 1120110---1100--00-??001151--2-1000-2121??10-11101000-------
0---00---------10010-10-110???000111100111011011010111100010001101000000-0-
010101011000000111

Mastigoteuthis_agassizi 1120110---1100--00-??001151--3-1110-2121??11-0------0-------
101000---------10011010-10010-00010110011101101101011110001000113100000-0-
010101011000000111

Octopoteuthis_sicula 1120110---1100--00-??001154--3-1110-2121??10-1110010110-0-
100---00---------10011010-110???000111100111011011010111000010001101000000-0-
010101011000000111

Ommastrephes_bartramii 112011100-1101000100--00-52--0-1000-2121??11-11101000-----
--101010---------10110-10-1000110001011001110110110111101000100011210000000-0-
010101011000100111

Onychoteuthis_banksii 11201110??1101000100--10-52-01-1100-2121??11-10--
110111110100---00---------10110-10-
100011000101100111011011011111100010011101000000-0-010101011000000111

Thysanoteuthis_rhombus 1120110---1100--00-??000-33--5-1100-2121??01-11101100-------
101000---------10110-10-
1000101001011001110110110111111000100011210000????010101011000100111

Sepia_officinalis 11201011101100--1110--10-14-1?-10?0-2020??0?-0------0-------0---00-
--------10200-10-10110-100101100111011011011111010010001101001000-0-
010001101000100?00

Heteroteuthis_hawaiiensis 0---------------------------?------------------------------------0---------
1000??10-10110-1001011001110110110101110000100011010010000-0-010001101010000100

Rossia_pacifica 1100110---1100--01-??010-51-00-1100-2120??11210--11?0-------
111000---------10001010-10110-100101100111011111111011???00100011?1001000-0-
010001101010000100

Spirula_spirula 11110-1110---1110-1---------------------??---------------------------------
1001??10-10110-10010110011111111111111110000100011010000000-0-010001101000100100

Cirrothauma_murrayi 1110110---0010--01011000-0-----?---0----??----------------------0---
------1000111110-----1001000-1210-10-10-0010---00110010-0000121111111010000100011000

Opisthoteuthis_agassizi 1110110---0010--01011000-0---------0----??----------------------0-----
----1000111110-----1011000-1210-10-10-0010---00010010-000012111110-010000100011000

Stauroteuthis_syrtensis 1110110---0010--01011000-0---------0----??----------------------0------
---1000111110-----1011000-1210-10-10-0010---00010010-0000121111111010000100011000

Amphitretus_pelagicus 0--0---
??1110-----1001000-1210-10-11010?0---0010002??01021?????101100101010?1000

Argonauta_nodosa 0--0---
??1110-----1001000-121101101101010---0010001130002121111110110010101001000

Haliphron_atlanticus 1110110---0010--00011000-0-----?---2----??----------------------0-----
----0---111110-----1001000-1210-1101101010---1010001100002121111110110010101001000

Japetella_diaphana 0--0---
??1110-----1001000-1210-10-10-1010---00100000-01021211111101100101010?1000

Keuppia_hyperbolaris 1110110---0010--00011000-0---------0----??----------------------0----
-----????111??0-----10??0??????????????10???????????????????????????????????????1000

Keuppia_levante 1110110---0010--00011000-0---------0----??----------------------0-------
--0---111110-----00010?????1101101101??1000

Octopus_vulgaris 1110110---0010--00011000-0---------2----??----------------------0-------
--0---111110-----1001000-121101101101010---00100000-00021211111101100101010001000

Ocythoe_tuberculata 0--0---
??1110-----0001000-121101101101010---1010001110002121111110110010101001000

Palaeoctopus_newboldi 1110110---0010--00011000-0---------0----??----------------------0---
------10?0111110-----?0010?11????????????1??????0????????????????????????????????1000

Styletoctopus_aff_annae 1110110---0010--00011000-0---------2----??----------------------0-----
----????111??0-----???????????????????1??????0???????????????????????????????1000

Geopeltis_simplex 1120110---1110--01010-00-333-110-?00201-0-023???????110-
10100---?0---------
??

Jeletzkyteuthis_coriaceus 1120110---1110--01010-00-244-0111100211-11011???????1110????0---?1???????????????1100-----???

Leptoteuthis_gigas 1120110---1110--01010-00-243-1100201212011001???????111010100---00---------11100-11?0-----101100????????????????????00???????????????????????????????????

Loligosepia_aalensis 1120110---1110--01010-00-244-0111100201-11011???????1110????0---?1?????????????0-11?0-----?0??0??????????????1??????0????????????????????????????????????

Loligosepia_bucklandi 1120110---1110--01010-00-144-0011100201-11011???????1110????0---?0---------??

Parabelopeltis_flexuosa 1120110---1110--01010-00-133-010-?00201-1001211101111???????0---?1??

Proteroctopus_ribeti 1120110---1110--01010010-143000???0020????44110-110110-??110---00---------10001111?0-----00110011??1101101100??0---00????????00?????????????????000??0??

Boreopeltis_sagittata 1120110---1010--0100--10-3-31-10--00210----1-???????11101010111100---------??

Boreopeltis_smithi 1120110---1010--0100--10-3-21-00--00210----1-???????11101010111100---------???????????????????????????????????1???

Dorateuthis_syriaca 1120110---1010--0100--10-4-20-00--00210----1-10--11?11111010111000---------10000-11?0-----001?0??????????????1??????01????????????????????????????????????

Plesioteuthis_prisca 1120110---1010--0100--10-4-30-00--00210----0-10--10011111010101010---------100?0-1??0-----101100????10-10-???1???????1???????????????????????????????????

Plesioteuthis_subovata 1120110---10?0--0100--00-4-?--?0--0???20---0-??????11111111101010---------??????1??0-----??1???

Senefelderiteuthis_tricarinata 1120110---1010--0100--10-4-3?-00--00210----1-???????110-1010111000---------10000-11?0-----101?00????????????????????0????????????????????????????????????

Actinosepia_canadensis 11?0110---1110--01010-00-110-0110010202110044???????1110101011010100?001011??

Glyphidopsis_waagei 1120110---1110--01011000-111-1111100??2010023???????111110100---0101?000?01????10????????????????????????????1???

Glyphiteuthis_abisaadiorum 1120110---1110--01011000-300-011110020210-023???????111110100---01?1??0?101110?0-1????????001?0??????????????1????????????????00?????????????????????????

Glyphiteuthis_freijii 1120110---1110--01011000-200-011111020210-0231110110111110100---01???0111011001011?0-----00??0?????????????????????0?????????00?????????????????????????

Glyphiteuthis_libanotica 1120110---1110--01011000-311-011111020210-02310--110111110100---0111200010110?00-1??0-----00?10?0-???

Glyphiteuthis_minor 11?0110---1110--01011000-111-1111100202010034???????111110100---01??2?0??

Glyphiteuthis_ornata 1120110---1110--01011000-122-0111110202110033???????111110100---01?1??011?0??

Glyphiteuthis_rhinophora 1120110---1110--0?0??????2?????100?0??21??03?10--110111110100---01???

Muensterella_scutellaris 1120110---1110--01011100-001-121130020210-044???????1111100-0---00---------112?111??0-----100100???10-???1101??????0????????????????????????????????????

Palaeololigo_oblonga 1120110---1110--01011000-211-131120?1-2?0-
0121111110111110100---00---------1010??1??0-----
???????????????????1???

Rachiteuthis_donovani 1120110---1110--01011000-400-111000020211001110--
11011111010111000---------10110-11?0-----
00??0??????????????1??????01??????0????????????????????????????

Styloteuthis_convexa 11?0110---1110--01011000-4??-0111?000-
??10044???????1111??100---00---------
??

Teudopsinia_haasi 11?0110---1110--01011100-211-111120020210-
012111111?111110100---00---------
??

Teudopsis_bollensis 11?0110---1110--01011000-211-11111001-
2110023???????111110100---00---------
??

Teudopsis_bunelii 11?0110---1110--01011000-321-011130020210-
011???????111110100---00---------
??

Teudopsis_jeletzkyi 11?0110---1110--0?0???00-121-?111100212?1102410--
110111110100---00---------
??

Teudopsis_subcostata 11?0110---1110--01011000-121-
0111100202111034???????111110100---00---------
??

Trachyteuthis_bacchiai 11?0110---1110--0?0???00-211-11110002020100230------0-------0--
-
01??2???000??
??

Trachyteuthis_covacevichi 11?0110---1110--0?0?????111?01111001-210-034???????0-------
0---
01001100000???????????????????????????????????1??
???

Trachyteuthis_hastiformis 1120110---1110--01011000-111-01110001-200-0340------0-------0-
--
011020000001100??1???
????

Trachyteuthis_nusplingensis 11?0110---1110--0?0???00-111-11110001-200-034???????0-------0---01002111000???

Trachyteuthis_teudopsiformis 11?0110---1110--0101100??111-01111001-21100341???110111110100---010?1100000??

Vampyronassa_rhodanica 1120110---1??0--01010-???1?????0-????????????0------0-------0---00---------10000-1??0-----10110????10-10-10-0??0??

Vampyroteuthis_infernalis 1120110---1110--01010-10-2320021000121200-1330------10--100-0---00---------1100111100-----10110011?00--0--10-011????00100000-100011101011010001000000000

Acanthoteuthis_speciosus 11211111101??11?1100--?0-4?????0-????????????0------0-------0---00---------100?0-10-0-----010100????110-101100???????01??????????????????????????????????

Idahoteuthis_parisiana 1120110---1010--0100--?0-?-??-?1-?0???21??-?????????111110??0---00---------1001??????????????????????????????1???????0???????????????????????????????????

Belemnotheutis_antiquus 1121111111011?1111100--?0-1?????113????20?????0------0-------0---00---------????1010-0-----010100???????10-10-1???

Gordoniconus_beargulchensis 112110111?1001100100--00-4-----0--0-211----1-0------0-------0---00---------0---0-10-0-----?0??0??????????????0??????00???????????????????????????????????

Syllipsimopodi_bideni 1120110---1000--0100--00-2-2--00--00201----1-??????111010110---00---------10010-1?-0-----000100????1101101101??????00???????????????????????????????????

Tip date calibrations:

Nautilus_pompilius=Fixed(469.4)
Hibolithes_semisulcatus=Fixed(237.0)
Belemnotheutis_antiquus=Fixed(165.3)
Phragmoteuthis_bisinuata=Fixed(254.2)
Doryteuthis_amerigo_pealeii=Fixed(2.58)
Sepia_officinalis=Fixed(72.2)
Spirula_spirula=Fixed(154.8)
Argonauta_nodosa=Fixed(33.9)
Keuppia_hyperbolaris=Fixed(100.5)
Keuppia_levante=Fixed(100.5)
Palaeoctopus_newboldi=Fixed(85.7)
Styletoctopus_aff_annae=Fixed(100.5)
Geopeltis_simplex=Fixed(184.2)
Jeletzkyteuthis_coriaceus=Fixed(184.2)
Leptoteuthis_gigas=Fixed(154.8)
Loligosepia_aalensis=Fixed(184.2)
Loligosepia_bucklandi=Fixed(184.2)
Parabelopeltis_flexuosa=Fixed(184.2)
Boreopeltis_sagittata=Fixed(149.2)
Boreopeltis_smithi=Fixed(100.5)
Dorateuthis_syriaca=Fixed(126.5)
Plesioteuthis_prisca=Fixed(154.8)
Plesioteuthis_subovata=Fixed(149.2)
Senefelderiteuthis_tricarinata=Fixed(149.2)
Actinosepia_canadensis=Fixed(83.7)
Glyphidopsis_waagei=Fixed(100.5)
Glyphiteuthis_abisaadiorum=Fixed(100.5)
Glyphiteuthis_freijii=Fixed(100.5)
Glyphiteuthis_libanotica=Fixed(100.5)
Glyphiteuthis_minor=Fixed(93.9)
Glyphiteuthis_ornata=Fixed(93.9)
Glyphiteuthis_rhinophora=Fixed(100.5)
Muensterella_scutellaris=Fixed(154.8)
Palaeololigo_oblonga=Fixed(149.2)
Rachiteuthis_donovani=Fixed(100.5)
Styloteuthis_convexa=Fixed(93.9)
Teudopsinia_haasi=Fixed(149.2)
Teudopsis_bollensis=Fixed(184.2)
Teudopsis_bunelii=Fixed(184.2)
Teudopsis_jeletzkyi=Fixed(184.2)
Teudopsis_subcostata=Fixed(184.2)
Trachyteuthis_bacchiai=Fixed(100.5)
Trachyteuthis_covacevichi=Fixed(161.5)
Trachyteuthis_hastiformis=Fixed(154.8)

Trachyteuthis_nusplingensis=Fixed(154.8)
Trachyteuthis_teudopsiformis=Fixed(149.2)
Vampyronassa_rhodanica=Fixed(165.3)
Proteroctopus_ribeti=Fixed(165.3)
Gordoniconus_beargulchensis=Fixed(330.3)
Syllipsimopodi_bideni=Fixed(330.3)
Idahoteuthis_parisiana=Fixed(249.9)
Acanthoteuthis_speciosus=Fixed(165.3)

III. SUPPLEMENTARY NOTES

Character transitions in the Bayesian tip-dated neocoleoid cephalopod phylogeny; refer to Supplementary Figure 8.

- Node 4 – **Coleoidea**
    - 2. Shell location in relation to the rest of the body: 0/1 -» 1
    - 5. Discrete proostracum developed: 0/1 -» 1
    - 19. Shell coiled: 0/2 -» 0
    - 82. Number of appendage pairs: 0/1 -» 1
    - 118. Calcareous elements in the beak: 0/1 -» 0
- Node 158 – **Neocoleoidea**
    - 6. Anterodorsally extended proostracum: 0/1 -» 1
    - 76. Fins: 0 -» 1
    - 94. Suckers on any appendage: 0/1 -» 1
    - 110. Ink sac: 0 -» 1
    - 112. Chromatophores: 0/1 -» 1
    - 137. Posterior salivary gland: 0/1 -» 1
    - 149. Spermatophore type: 0/2 -» 0
- Node 55 – **Vampyropoda**
    - 4. Shell extent along D-V axis: 1 -» 0
    - 7. Septate phragmocone: 1 -» 0
    - 14. Primordial rostrum: 1 -» 0
    - 53. Dorsal median field / rachis in transverse section: 0 -» 1
- Node 56
    - 13. Lateral fields (or homologous wings): 0 -» 1
    - 39. Shape of anterior tip of median field: 1/2 -» 2
- Node 57 – **Octobrachia**
    - 46. Ventral median field/rachis in transverse section: 0/1 -» 1
    - 79. Posterior fin termination: 1 -» 0
    - 83. Reduction of appendage pair II: 0/1 -» 1
    - 93. Cirri or spines on arms: 0/1 -» 1
    - 102. Suckers (on arms) proximally: 0/1 -» 0
- ❖ Node 145 – **Prototeuthidina**
    - 26. Gladius length / gladius width: 1/4 -» 4
    - 38. Nature of convex end: 0/1 -» 1
    - 61. Lateral reinforcements on the median field/rachis: 0 -» 1
    - 118. Calcareous elements in the beak: 0 -» 1
- ❖ Node 153 – ***Plesioteuthis***
    - 44. Angle between inner/median asymptote and midline: 1 -» 0
    - 56. Form of dorsal interruption(s): 0/1 -» 1
    - 62. Extent of lateral reinforcements: 1 -» 0
    - 65. Thin lateral plates on the dorsal median field/rachis: 0 -» 1

- ❖ Node 146
  - 23. 'Cone flags': 0/1 -» 1
  - 39. Shape of anterior tip of median field: 0/2 -» 0
- ❖ Node 148
- ❖ Node 149
  - 28. Wing length / rachis length: 3 -» 2
- Node 58 – **Octopodiformes**
  - 12. Distinct hyperbolar zones (or homologous vanes): 0 -» 1
  - 20. Condition of the primary cone: 0 -» 1
  - 38. Nature of convex end: 0/1 -» 0
  - 98. Sucker-stalk on arms: 0/1 -» 1
  - 113. Buccal crown: 0/1 -» 0
  - 130. Statocyst outer capsule: 0/1 -» 1
  - 132. Inferior frontal lobe system of the brain: 0/1 -» 1
  - 134. Superior buccal lobe: 0/1 -» 1
  - 136. Relative position of digestive gland duct appendages (DGDA) and the nephridial coelom: 0/1 -» 1
  - 141. Nidamental glands: 0/1 -» 0
  - 144. Oviducal gland symmetry: 0/1/2 -» 0
- ❖ Node 128
  - 26. Gladius length / gladius width: 1/4 -» 1
  - 47. Form of ventral interruption(s): 0 -» 1
  - 81. Maximum length of unmodified arms compared to mantle length: 0/1 -» 1
- ❖ Node 129
  - 31. Wing width / rachis width at wing: 0/1 -» 1
  - 56. Form of dorsal interruption(s): 0/1 -» 0
  - 84. Loss of appendage pair II: 0/1 -» 0
- ➢ Node 130 – **Loligosepiina**
  - 39. Shape of anterior tip of median field: 2 -» 1
- ➢ Node 131 – **Geopeltidae**
  - 27. Vane length / rachis length: 3/4 -» 3
- ➢ Node 134 – **Loligosepiidae**
  - 27. Vane length / rachis length: 3/4 -» 4
  - 28. Wing length / rachis length: 3 -» 4
  - 32. Position of greatest width of median field / rachis: 0 -» 1
  - 34. Rachis width at vane insertion / rachis width 2/3 of the way between vane insertion and the anterior: 0/1 -» 1
  - 45. Angle between outer/lateral asymptote and midline: 1/3 -» 1
- ➢ Node 135
  - 26. Gladius length / gladius width: 1 -» 2
  - 66. Granules (tubercles) on the gladius/proostracum: 0 -» 1
- ❖ Node 139 – **Vampyromorphida**
  - 46. Ventral median field / rachis in transverse section: 1 -» 0

    - 91. Interbrachial web: 0/1 -» 1
    - 108. Suckers (on arms) distally: 0/1 -» 0
    - 110. Ink sac: 0/1 -» 0
- ❖ Node 140
    - 26. Gladius length / gladius width: 1 -» 2
    - 33. Position of greatest width of median field / rachis: 0/1 -» 0
    - 36. Outline of posterior margin of lateral field / wings: 0/1 -» 1
    - 38. Nature of convex end: 0/1 -» 1
    - 77. Number of fin pairs: 0 -» 1
- Node 59 – **Panoctopoda nov.**
    - 21. Primary cone open ventrally: 0 -» 1
    - 28. Wing length / rachis length: 3 -» 1
    - 31. Wing width / rachis width at wing: 0/1 -» 1
    - 32. Position of greatest width of median field/rachis: 0 -» 1
    - 40. Nature of convex tip: 0 -» 1
    - 56. Form of dorsal interruption(s): 0/1 -» 1
- ❖ Node 119 – **Teudopseina**
- ❖ Node 120
    - 26. Gladius length / gladius width: 1/3/4 -» 4
    - 42. Inflexion in gladius outline where lateral asymptote intersects margin: 0/1 -» 0
- ❖ Node 121
    - 30. Vane width / rachis width at vane: 0 -» 1
    - 78. Shape of fins: 0/1 -» 1
- ❖ Node 122
    - 26. Gladius length / gladius width: 4 -» 2
    - 27. Vane length / rachis length: 0/1/2 -» 1
    - 34. Rachis width at vane insertion / rachis width 2/3 of the way between vane insertion and the anterior: 0 -» 2
    - 41. Inflexion in gladius outline where lateral asymptote intersects margin: 1 -» 0
    - 45. Angle between outer/lateral asymptote and midline: 1/2/4 -» 2
    - 47. Form of ventral interruption(s): 0 -» 1
    - 49. Form of ventral interruption(s): 0/1 -» 1
- Node 60
    - 26. Gladius length / gladius width: 1/4 -» 1
    - 34. Rachis width at vane insertion / rachis width 2/3 of the way between vane insertion and the anterior: 0 -» 1
    - 44. Angle between inner/median asymptote and midline: 1 -» 2
    - 80. Maximum length of unmodified arms compared to mantle length: 0 -» 1
    - 97. Sucker-stalk on arms: 0/1 -» 0
- ❖ Node 92 – **Trachyteuthidae**
    - 27. Vane length / rachis length: 0/1/2 -» 1
    - 42. Inflexion in gladius outline where lateral asymptote intersects margin: 0/1 -» 0
- ❖ Node 93
    - 66. Granules (tubercles) on the gladius/proostracum: 0 -» 1

  - 77. Number of fin pairs: 0/1 -» 1
- Node 111 – ***Trachyteuthis***
  - 37. Shape of posterior end of median field / rachis: 1/2 -» 1
  - 44. Angle between inner/median asymptote and midline: 2 -» 3
  - 45. Angle between outer/lateral asymptote and midline: 3/4 -» 4
  - 68. Arrangement of granules in the posterior half: 0/1 -» 0
  - 75. Width of granulated area at vane / rachis width at vane: 0/1 -» 0
- Node 112
  - 69. Granule size-change from posterior to anterior: 2 -» 1
  - 70. Granule size-change from midline towards lateral margin: 0/1 -» 1
- Node 115
  - 34. Rachis width at vane insertion / rachis width 2/3 of the way between vane insertion and the anterior: 1 -» 0
  - 40. Nature of convex tip: 1 -» 0
  - 41. Inflexion in gladius outline where lateral asymptote intersects margin: 0/1 -» 0
  - 53. Dorsal median field / rachis in transverse section: 0/1 -» 0
- Node 94
  - 37. Shape of posterior end of median field / rachis: 1/2 -» 2
  - 70. Granule size-change from midline towards lateral margin: 0/1 -» 0
  - 81. Maximum length of unmodified arms compared to mantle length: 0/1 -» 0
- Node 106
  - 30. Vane width / rachis width at vane: 0 -» 1
  - 40. Nature of convex tip: 1 -» 0
- Node 107
- Node 95
  - 28. Wing length / rachis length: 1 -» 0
  - 35. Concave inflexion or inflexions in median field / rachis: 0 -» 1
- Node 96
  - 33. Position of greatest width of median field / rachis: 1 -» 0
  - 34. Rachis width at vane insertion / rachis width 2/3 of the way between vane insertion and the anterior: 1 -» 0
- Node 99 – ***Glyphiteuthis***
  - 45. Angle between outer/lateral asymptote and midline: 3/4 -» 3
  - 68. Arrangement of granules in the posterior half: 0/1 -» 1
  - 73. Lateral margins of the granulated area meet anteriorly at the anterior edge of the median field / rachis?: 0 -» 1
- Node 100
  - 26. Gladius length / gladius width: 1 -» 3
  - 41. Inflexion in gladius outline where lateral asymptote intersects margin: 0/1 -» 0
  - 74. Lateral margins of posterior 1/3 of granulated area: 0/1 -» 0
  - 75. Width of granulated area at vane / rachis width at vane: 0/1 -» 1
  - 80. Maximum length of unmodified arms compared to mantle length: 1 -» 0
- Node 103

  - 72. Row of relatively coarse granules along median field / rachis: 0/1 -» 1
  - 75. Width of granulated area at vane / rachis width at vane: 0/1 -» 0
- Node 61
  - 45. Angle between outer/lateral asymptote and midline: 1/3/4 -» 4
- ❖ Node 89
  - 27. Vane length / rachis length: 0/1/2 -» 2
  - 42. Inflexion in gladius outline where lateral asymptote intersects margin: 0/1 -» 1
- Node 62
  - 26. Gladius length / gladius width: 1 -» 0
  - 81. Maximum length of unmodified arms compared to mantle length: 0/1 -» 1
  - 91. Interbrachial web: 0/1 -» 1
  - 93. Cirri or spines on arms: 0/1 -» 0
- Node 63 – **Octopoda**
  - 3. Shell extent along A-P axis: 2 -» 1
  - 11. Median field (or homologous rachis): 1 -» 0
  - 12. Distinct hyperbolar zones (or homologous vanes): 1 -» 0
  - 18. Conus: 1 -» 0
  - 84. Loss of appendage pair II: 0/1 -» 1
  - 99. Shape of stalks attached to the arm muscles: 0/1 -» 1
  - 100. Lining of arm suckers: 0/1/2 -» 2
  - 111. Nuchal cartilage: 0/1 -» 0
  - 123. Funnel connection to mantle: 0/1 -» 1
  - 126. Funnel valve: 0/1 -» 0
  - 131. Photosensitive vesicles: 0/1/2 -» 2
  - 133. Inferior frontal lobe system of the brain: 0/1 -» 1
  - 135. Superior buccal lobe: 0/1 -» 1
  - 140. Gill-lamellae attachment: 0/1 -» 1
  - 145. Oviducal gland position: 0/1 -» 1
  - 150. Dorsal mantle cavity: 0/1 -» 1
- ❖ Node 64
- ❖ Node 65
  - 77. Number of fin pairs: 0/1 -» 0
- ❖ Node 66 – **Cirrata**
  - 18. Conus: 0 -» 1
  - 105. Suckers (on arms) medially: 0/1 -» 0
  - 108. Suckers (on arms) distally: 0/1 -» 0
  - 110. Ink sac: 1 -» 0
  - 120. Horizontal arm septa inserted in the arm muscles: 0/1 -» 1
  - 138. Posterior salivary gland position: 0/1 -» 1
  - 139. Branchial canal: 0/1 -» 0
  - 143. Right oviduct: 0/1 -» 0
  - 149. Spermatophore type: 0/1 -» 1
- ❖ Node 68

  - 93. Cirri or spines on arms: 0 -» 1
  - 119. Radula: 1 -» 0
- Node 73
  - 76. Fins: 1 -» 0
  - 102. Suckers (on arms) proximally: 0 -» 1
  - 105. Suckers (on arms) medially: 0/1 -» 1
- Node 74
  - 36. Outline of posterior margin of lateral field / wings: 0 -» 2
- Node 75 – **Incirrata**
  - 129. Cornea: 0/2 -» 2
  - 139. Branchial canal: 0/1 -» 1
  - 147. Arm-pair IV hectocotylization or other sexual modifications: 0/1 -» 1
- Node 76
- ❖ Node 82
  - 117. Hydrostatic organ (swim-bladder): 0 -» 1
  - 124. Funnel locking apparatus: 0/1 -» 1
- Node 77
  - 123. Funnel connection to mantle: 1 -» 0
  - 124. Funnel locking apparatus: 0/1 -» 0
- Node 78
  - 1. Shell/gladius/vestigial shell: 0/1 -» 0
  - 102. Suckers (on arms) proximally: 1 -» 0
  - 105. Suckers (on arms) medially: 1 -» 0
  - 127. Eye shape: 0 -» 1

Belemnoidea + Decabrachia

- Node 6
  - 6. Anterodorsally extended proostracum: 0/1 -» 1
  - 10. Position of siphuncle within the shell: 0/1 -» 0
  - 12. Distinct hyperbolar zones (or homologous vanes): 0 -» 1
  - 39. Shape of anterior tip of median field: 1/2 -» 2
  - 45. Angle between outer/lateral asymptote and midline: 1/2/3/4 -»2
  - 76. Fins: 0 -» 1
  - 94. Suckers on any appendage: 0/1 -» 1
  - 110. Ink sac: 0 -» 1
  - 112. Chromatophores: 0/1 -» 1
  - 137. Posterior salivary gland: 0/1 -» 1
  - 149. Spermatophore type: 0/2 -» 0
- ❖ Node 7 – **Belemnoidea**
  - 13. Lateral fields (or homologous wings): 0 -» 1
  - 92. True arm hooks: 0 -» 1
- ❖ Node 8
  - 16. Primordial rostrum length: 0/1 -» 1
  - 17. Rostrum or guard: 0 -» 1
- ❖ Node 10

- Node 14 – **Decabrachia**
    - 85. Modification of appendage IV into a tentacle: 0 -» 1
    - 91. Interbrachial web: 0 -» 1
    - 96. Sucker symmetry: 0 -» 1
    - 98. Sucker-stalk on arms: 0/1 -» 0
    - 99. Shape of stalks attached to the arm muscles: 0/1 -» 0
    - 100. Lining of arm suckers: 0/1/2 -» 1
    - 111. Nuchal cartilage: 0/1 -» 1
    - 115. Number of buccal lappets (supports): 0/3 -» 0
    - 123. Funnel connection to mantle: 0/1 -» 1
    - 124. Funnel locking apparatus: 0/1 -» 1
    - 131. Photosensitive vesicles: 0/1/2 -» 0
    - 132. Inferior frontal lobe system of the brain: 0/1 -» 0
    - 136. Relative position of digestive gland duct appendages (DGDA) and the nephridial coelom: 0/1 -» 0
    - 142. Accessory nidamental glands: 0/1 -» 1
    - 143. Right oviduct: 0/1 -» 0
    - 144. Oviducal gland symmetry: 0/1/2 -» 1
    - 148. Arm-pair V hectocotylization or other sexual modifications: 0/1 -» 1
    - 151. Collagenous tunic on mantle: 0/1 -» 1
- Node 15
    - 4. Shell extent along D-V axis: 1 -» 0
    - 14. Primordial rostrum: 1 -» 0
    - 32. Position of greatest width of median field / rachis: 0/1 -» 1
    - 129. Cornea: 0 -» 1
- Node 16
    - 7. Septate phragmocone: 1 -» 0
    - 46. Ventral median field/rachis in transverse section: 0 -» 1
    - 81. Maximum length of unmodified arms compared to mantle length: 0/1 -» 0
- ❖ Node 48
    - 79. Posterior fin termination: 0/1 -» 0
    - 111. Nuchal cartilage: 1 -» 0
    - 116. Suckers on buccal membrane: 0/1 -» 0
- ❖ Node 50 – **Sepiolida**
    - 88. Tentacle suckers: 0/1 -» 1
    - 139. Branchial canal: 0/1 -» 0
    - 146. Arm-pair I hectocotylization or other sexual modifications: 0 -» 1
    - 148. Arm-pair V hectocotylization or other sexual modifications: 1 -» 0
- Node 17
    - 18. Conus (primary cone): 0/1 -» 0
    - 26. Gladius length / gladius width: 1/4 -» 4
    - 27. Vane length / rachis length: 1/3/4 -» 3
    - 40. Nature of convex tip: 0 -» 1
    - 43. Tapering of the hyperbolar zone / vane both anteriorly and posteriorly (forming a spindle shape): 0/1 -» 1
    - 53. Dorsal median field / rachis in transverse section: 0/1 -» 1

  - 79. Posterior fin termination: 0/1 -» 1
  - 87. Retractile tentacles: 1 -» 0
  - 91. Interbrachial web: 1 -» 0
  - 115. Number of buccal lappets (supports): 0 -» 1
  - 139. Branchial canal: 0/1 -» 1
- Node 18
  - 15. Mineralized primordial rostrum: 0/1 -» 0
  - 30. Vane width / rachis width at vane: 0/2/3 -» 3
  - 62. Extent of lateral reinforcements: 0/1 -» 0
  - 129. Cornea: 1 -» 0
  - 142. Accessory nidamental glands: 1 -» 0
  - 143. Right oviduct: 0 -» 1
- Node 19 – **Bathyteuthida**
  - 27. Vane length / rachis length: 3 -» 2
  - 44. Angle between inner/median asymptote and midline: 0/1 -» 0
  - 88. Tentacle suckers: 0/1 -» 1
  - 109. Suckers (on arms) distally: 0 -» 1
  - 116. Suckers on buccal membrane: 0/1 -» 1
  - 148. Arm-pair V hectocotylization or other sexual modifications: 0/1 -» 0
- Node 22 – **Oegopsida**
  - 89. Tentacle locking apparatus: 0 -» 1
  - 116. Suckers on buccal membrane: 0/1 -» 0
  - 152. Stellar ganglia connected by a commissure: 0/1 -» 1
  - 153. Development of a commissure between the mantle and visceral nerve: 0/1 -» 1
- Node 23
  - 78. Shape of fins: 0 -» 1
  - 148. Arm-pair V hectocotylization or other sexual modifications: 0/1 -» 1
- Node 24
  - 44. Angle between inner/median asymptote and midline: 0/1 -» 1
  - 88. Tentacle suckers: 0/1 -» 0
- Node 25
  - 18. Conus (primary cone): 0/1 -» 1
  - 20. Condition of the primary cone: 0/1 -» 1
  - 34. Rachis width at vane insertion / rachis width 2/3 of the way between vane insertion and the anterior: 0 -» 1
  - 53. Dorsal median field / rachis in transverse section: 0/1 -» 1
  - 56. Form of dorsal interruption(s): 0/1 -» 0
  - 61. Lateral reinforcements on the median field / rachis: 0/1 -» 0
  - 114. Buccal membrane connective attachment to arm-pair V: 1 -» 0
- Node 29
  - 7. Septate phragmocone: 0 -» 1
  - 8. Mineralized phragmocone: 0/1 -» 0
  - 14. Primordial rostrum: 0 -» 1
  - 16. Primordial rostrum length: 0/1 -» 0
  - 18. Conus (primary cone): 0/1 -» 1

  - 19. Shell coiled: 0/1 -» 0
  - 26. Gladius length / gladius width: 3/4/5 -» 5
- Node 31
  - 20. Condition of the primary cone: 0/1 -» 0
  - 27. Vane length / rachis length: 3 -» 2
  - 44. Angle between inner/median asymptote and midline: 0/1 -» 1
  - 88. Tentacle suckers: 0/1 -» 0
- Node 34
  - 50. Anterior ventral interruption(s): 1 -» 0
  - 57. Dorsal interruption(s) at 25% of rachis length from anterior: 1 -» 0
  - 148. Arm-pair V hectocotylization or other sexual modifications: 0/1 -» 0
- Node 44 – ***Histioteuthis***
  - 44. Angle between inner/median asymptote and midline: 0/1 -» 0
  - 55. Form of dorsal interruption(s): 1 -» 0
  - 61. Lateral reinforcements on the median field / rachis: 0/1 -» 1
  - 80. Maximum length of unmodified arms compared to mantle length: 0 -» 1
  - 88. Tentacle suckers: 0/1 -» 1
  - 91. Interbrachial web: 0 -» 1
  - 114. Buccal membrane connective attachment to arm-pair V: 1 -» 0
  - 121. Luminous autogenic organs: 0 -» 1
  - 127. Eye shape: 0 -» 1
  - 128. Asymmetrical eyes, with one significantly enlarged: 0 -» 1
  - 146. Arm-pair I hectocotylization or other sexual modifications: 0 -» 1
- Node 35
  - 24. Ventral folding of posterolateral gladius margin: 0 -» 1
  - 26. Gladius length / gladius width: 3/4/5 -» 5
  - 61. Lateral reinforcements on the median field / rachis: 0/1 -» 0
- Node 36
  - 27. Vane length / rachis length: 3 -» 1
  - 111. Nuchal cartilage: 1 -» 0
- Node 37
  - 89. Tentacle locking apparatus: 0/1 -» 0
- Node 40
  - 44. Angle between inner/median asymptote and midline: 0/1 -» 0
  - 47. Form of ventral interruption(s): 0 -» 1
  - 86. Loss of tentacles in adulthood: 0 -» 1
  - 95. Sucker-hooks: 0 -» 1

IV. SUPPLEMENTARY DISCUSSION

Taxonomic revisions following results of the phylogenetic analysis (Figure 6).

Class: CEPHALOPODA Cuvier 1795
Subclass COLEOIDEA Bather, 1888
Clade: NEOCOLEOIDEA Haas, 1997
Clade: VAMPYROPODA von Boletzky, 1992
Superorder OCTOBRACHIA Haeckel, 1866
Clade: OCTOPODIFORMES Berthold & Engeser, 1987
Suborder TEUDOPSEINA Starobogatov, 1983

Family TRACHYTEUTHIDAE Naef, 1921

Genus *Justinianiteuthis* gen. nov.

Type species: *Glyphiteuthis rhinophora* Fuchs et al., 2010

Etymology: Named after Roman Emperor Justinian II Rhinotmetos, because of the name of the type species.

Description: The same as for the type and only species.

Comparison: Unlike in *Glyphiteuthis*, the median field is widest anterior to the hyperbolar zone insertion.

Included species: *Justinianiteuthis rhinophora* Fuchs et al., 2010

Genus *Fisheriteuthis* gen. nov.

Type species: *Glyphiteuthis minor* Fritsch & Schlönbach, 1872

Etymology: After the palaeontologist Daniel C. Fisher, the undergraduate mentor of Christopher D. Whalen.

Description: The same as for the type and only species.

Comparison: Unlike in *Glyphiteuthis*, the ratio of hyperbolar zone width to median field width is greater than 0.25. The convex tip is rounded in *Fisheriteuthis*, not pointed as in *Glyphiteuthis*. Unlike *Glyphiteuthis*, the angle between the inner and median asymptotes is greater than 20°.

Included species: *Fisheriteuthis minor* Fritsch & Schlönbach, 1872

Genus *Edmunditeuthis* gen. nov.

Type species: *Trachyteuthis bacchiai* Fuchs and Larson, 2011b

Etymology: After St. Edmund the Martyr, the patron saint of pandemics, in memory of all those who lost their lives to COVID-19.

Description: The same as for the type and only species.

Comparison: Unlike in *Trachyteuthis*, the ratio of gladius length to gladius width is greater than 3, the angle between the inner/median asymptote and the midline is less than 15 degrees, and the angle between the outer/lateral asymptote and the midline is less than 20 degrees.

Included species: *Edmunditeuthis bacchiai* Fuchs and Larson, 2011b

Family INCERTAE SEDIS

Genus *Briggsiteuthis* gen. nov.

Type species: *Teudopsis bollensis* Voltz, 1836

Etymology: After the palaeontologist Derek E. G. Briggs, the PhD advisor of Christopher D. Whalen.

Description: The same as for the type and only species.

Comparison: Unlike in *Teudopsis*, there is a weak inflexion in the gladius outline where the lateral asymptote intersects the margin. Unlike in *Teudopsis*, *Fuchsiteuthis*, and *Suttoniteuthis*, the ratio of gladius length to gladius width is greater than or equal to 4, and the ratio of hyperbolar zone length to median field length is less than 0.5. Unlike in *Teudopsis* and *Suttoniteuthis*, the ratio of hyperbolar zone width to median field width at the hyperbolar zone is greater than 0.25. The median field width at the hyperbolar zone insertion relative to the median field width 2/3 of the way between the hyperbolar zone insertion and the anterior is less than 1; this ratio is greater than 2.5 in *Teudopsis*. The posterior end of the median field is flat in *Briggsiteuthis*, but it is convex in *Teudopsis*, *Fuchsiteuthis*, and *Suttoniteuthis*. Unlike in *Teudopsis* and *Suttoniteuthis*, the angle between the inner/median asymptote and the midline is between 10° and 15°. Unlike in *Teudopsis*, *Fuchsiteuthis*, and *Suttoniteuthis*, the angle between the outer/lateral asymptote and the midline is between 10° and 20°

Included species: *Briggsiteuthis bollensis* Voltz, 1836

Genus *Fuchsiteuthis* gen. nov.

Type species: *Teudopsis jeletzkyi* Riccardi, 2005

Etymology: After the palaeontologist Dirk Fuchs, for his extensive body of research on fossil octobrachians.

Description: The same as for the type and only species.

Comparison: Unlike in *Teudopsis,* there is a strong inflexion in the gladius outline where the lateral asymptote intersects the margin. Unlike in *Teudopsis* and *Briggsiteuthis*, the ratio of gladius length to gladius width is greater than or equal to 2 but less than 3. The median field width at the hyperbolar zone insertion relative to the median field width 2/3 of the way between the hyperbolar zone insertion and the anterior is less than 1; this ratio is greater than 2.5 in *Teudopsis*. Unlike in *Teudopsis*, *Briggsiteuthis,* and *Suttoniteuthis,* the convex end is pointed. Unlike in *Teudopsis* and *Suttoniteuthis*, the angle between the inner/median asymptote and the midline is between 10° and 15°. Unlike in *Teudopsis* and *Briggsiteuthis*, the angle between the outer/lateral asymptote and the midline is greater than 20°.

Included species: *Fuchsiteuthis jeletzkyi* Riccardi, 2005

Genus *Suttoniteuthis* gen. nov.

Type species: *Teudopsis subcostata* Münster, 1843

Etymology: After the palaeontologist Mark Sutton, for his pioneering work on neocoleoid interrelationships.

Description: The same as for the type and only species.

Comparison: Unlike in *Teudopsis,* there is a strong inflexion in the gladius outline where the lateral asymptote intersects the margin. Unlike in *Teudopsis* and *Briggsiteuthis*, the ratio of gladius length to gladius width is greater than or equal to 2 but less than 3. The median field width at the hyperbolar zone insertion relative to the median field width 2/3 of the way between the hyperbolar zone insertion and the anterior is less than 1; this ratio is greater than 2.5 in *Teudopsis*. Unlike in *Teudopsis, Briggsiteuthis*, and *Fuchsiteuthis*, the angle between the inner/median asymptote and the midline is greater than 15°. Unlike in *Teudopsis* and *Briggsiteuthis*, the angle between the outer/lateral asymptote and the midline is greater than 20°.

Included species: *Suttoniteuthis subcostata* Münster, 1843

V. SUPPLEMENTARY REFERENCES

Arkhipkin, A. I., Bizikov, V. A. & Fuchs, D. Vestigial phragmocone in the gladius points to a deepwater origin of squid (Mollusca: Cephalopoda). *Deep-Sea Research I* **61**, 109–122 (2012).

Bather, F. A. Shell-growth in Cephalopoda (Siphonopoda). *J. Nat. Hist.* **1**, 298–309 (1888).

Berthold, T. & Engeser, T. Phylogenetic analysis and systematization of the Cephalopoda (Mollusca). *Verhandlungen des Naturwissenschaftlichen Vereins in Hamburg* **29**, 187–220 (1987).

Brazeau, M. D. Problematic character coding methods in morphology and their effects. *Biol. J. Linn. Soc.* **104**, 489–498 (2011).

Cuvier, G. Second Mémoire sur l'organisation et les rapports des animaux à sang blanc, dans lequel on traite de la structure des Mollusques et de leur division en ordre, lu à la société d'Histoire Naturelle de Paris, le 11 prairial an troisième. *ou J. des Sci. des Lettres des Arts* **2**, 433–449 (1795).

Doguzhaeva, L. A. Two Early Cretaceous spirulid coleoids of the north-western Caucasus: their shell ultrastructure and evolutionary implications. *Palaeontology* **39**, 681–707 (1996).

Doguzhaeva, L. A. A rare coleoid mollusc from the Upper Jurassic of Central Russia. *Acta Palaeontol. Pol.* **45**, 389–406 (2000).

Doguzhaeva, L. A. *et al*. An Early Triassic gladius associated with soft tissue remains from Idaho, USA—a squid-like coleoid cephalopod at the onset of Mesozoic Era. *Acta Palaeontol. Pol.* **63**, 341-355 (2018).

Doguzhaeva, L. A. & Mutvei, H. Gladius composition and ultrastructure in extinct squid-like coleoids: *Loligosepia, Trachyteuthis* and *Teudopsis*. *Rev. Paléobiol.* **22**, 877–894 (1991).

Doguzhaeva, L. A. & Summesberger, H. Pro-ostraca of Triassic belemnoids (Cephalopoda) from Northern Calcareous Alps, with observations on their mode of preservation in an environment of northern Tethys which allowed for carbonization of non-biomineralized structures. *N. Jb. Geol. Paläont. Abh.* **266**, 31–38 (2012).

Donovan, D. T. Part M, Chapter 9C: Composition and structure of gladii in fossil Coleoidea. *Treatise Online* **75** (2016).

Doyle, P. & Shakides, E. V. The Jurassic belemnite suborder Belemnotheutina. *Palaeontology* **47**, 983-998 (2004).

Dzik, J. & Korn, D. Devonian ancestors of *Nautilus. Pal. Z.* **66**, 81–98 (1992).

Fritsch, A., & Schlönbach, G. J. C. U. *Cephalopoden der böhmischen Kreideformation*. (Řivnáč, 1872).

Fuchs, D. *et al. Glyphiteuthis rhinophora* n. sp., a trachyteuthidid (Coleoidea, Cephalopoda) from the Cenomanian (Late Cretaceous) of Mexico. *Palaontologische Zeitschrift* **84**, 523–532 (2010).

Fuchs, D. The "rostrum" - problem in coleoid terminology - an attempt to clarify inconsistencies. *Geobios* **45**, 29–39 (2012).

Fuchs, D. Part M, Chapter 23G: Systematic Descriptions: Octobrachia. *Treatise Online* **111** (2020).

Fuchs, D. & Donovan, D. Part M, Chapter 23C: Systematic Descriptions: Phragmoteuthida. *Treatise Online* **138** (2018).

Fuchs, D., Engeser, T. & Keupp, H. Gladius shape variation in coleoid cephalopod *Trachyteuthis* from the Upper Jurassic Nusplingen and Solnhofen Plattenkalks. *Acta Palaeontol. Pol.* **52**, 575– 589 (2007c).

Fuchs, D. & Iba, Y. The gladiuses in coleoid cephalopods: homology, parallelism, or convergence? *Swiss J. Palaeontol.* **134**, 187–197 (2015).

Fuchs, D. *et al*. The Muensterelloidea: phylogeny and character evolution of Mesozoic stem octopods. *Pap. Palaeontol.* **6**, 31–92 (2020).

Fuchs, D., Keupp, H. & Schweigert, G. First record of a complete arm crown of the Early Jurassic coleoid *Loligosepia* (Cephalopoda). *Pal. Z.* **87**, 431–435 (2013).

Fuchs, D., Keupp, H., Mitta, V. & Engeser, T., Ultrastructural analyses on the conotheca of the genus Belemnotheutis (Belemnitida: Coleoidea). in *Cephalopods Present and Past: New Insights and Fresh Perspectives* (eds. Landman, N. H., Davis, R. A. & Mapes, R. H.) 299–314 (Springer, 2007a).

Fuchs, D., Klinghammer, A. & Keupp, H. Taxonomy, morphology and phylogeny of plesioteuthidid coleoids from the Upper Jurassic (Jurassic, Tithonian) Plattenkalks of Solnhofen. *N. Jb. Geol. Paläont. Abh.* **245**, 239–252 (2007b).

Fuchs, D. & Larson, N. Diversity, morphology, and phylogeny of coleoid cephalopods from the Upper Cretaceous plattenkalks of Lebanon - part I: Prototeuthidina. *J. Paleontol.* **85**, 234–249 (2011a).

Fuchs, D. & Larson, N. Diversity, morphology, and phylogeny of coleoid cephalopods from the Upper Cretaceous plattenkalks of Lebanon - Part II: Teudopsina. *J. Paleontol.* **85**, 815–834 (2011b).

Fuchs, D. & Schultze, H.-P. *Trachyteuthis covacevichi* n. sp., a Late Jurassic coleoid cephalopod from the Paleopacific. *Fossil Record*, **11**, 39–49 (2008).

Fuchs, D. & Schweigert, G. First Middle – Late Jurassic gladius vestiges provide new evidence on the detailed origin of incirrate and cirrate octopuses (Coleoidea). *Pal. Z.* **92**, 203–217 (2018).

Fuchs, D., Stinnesbeck, W., Ifrim, C., Giersch, S., Gutierrez, J. M. P. & Frey, E. *Glyphiteuthis rhinophora* n. sp., a trachyteuthidid (Coleoidea, Cephalopoda) from the Cenomanian (Late Cretaceous) of Mexico. *Pal. Z.*, (2010).

Fuchs, D., von Boletzky, S. & Tischlinger, H. New evidence of functional suckers in belemnoid coleoids (Cephalopoda) weakens support for the 'Neocoleoidea' concept. *J. Molluscan Stud.* **76**, 404–406 (2010).

Fuchs, D. & Weis, R. Taxonomy, morphology and phylogeny of Lower Jurassic loligosepiid coleoids (Cephalopoda). *N. Jb. Geol. Paläont. Abh.* **249**, 93–112 (2008).

Fuchs, D. & Weis, R. Taxonomy, morphology and phylogeny of Lower Jurassic teudopseid coleoids (Cephalopoda). *N. Jb. Geol. Paläont. Abh.* **257**, 351–366 (2010).

Haeckel, E. Generelle morphologie der organismen, zweite band. Allgemeine entwick-lungsgeschichte der organismen. *Georg Reimer. Berlin.* **1** (1866).

Iba, Y., S. Sano, J. Mutterlose, & Kondo, Y. Belemnites originated in the Triassic — A new look at an old group. *Geology* **40**, 911–914 (2012).

Jereb, P. & Roper, C. F. E. Cephalopods of the world. An annotated and illustrated catalogue of cephalopod species known to date. Volume 1. Chambered nautiluses and sepioids (Nautilidae, Sepiidae, Sepiolidae, Sepiadariidae, Idiosepiidae and Spirulidae). *FAO Species Catalogue for Fishery Purposes* **4** (2005).

Jereb, P. & Roper, C. F. E. Cephalopods of the world. An annotated and illustrated catalogue of cephalopod species known to date. Volume 2. Myopsid and oegopsid squids. *FAO Species Catalogue for Fishery Purposes* **4** (2010).

Jereb, P., Roper, C. F. E., Norman, M. D., Julian, K. F. Cephalopods of the world. An annotated and illustrated catalogue of cephalopod species known to date. Volume 3. Octopods and vampire squids. *FAO Species Catalogue for Fishery Purposes* **4** (2014).

Klug, C. *et al*. Anatomy and evolution of the first Coleoidea in the Carboniferous. *Commun. Biol.* **2**, 280 (2019).

Klug, C., Günter, S., Fuchs, D., Kruta, I. & Tischlinger, H. Adaptations to squid-style high-speed swimming in Jurassic belemnitids. *Biol. Lett.* **12**, 20150877 (2016).

Klug, C., Schweigert, G., Fuchs, D. & De Baets, K. Distraction sinking and fossilized coleoid predatory behaviour from the German Early Jurassic. *Swiss J. Palaeontol.* **140**, (2021).

Košťák, M. Teuthoidea from the Bohemian Cretaceous Basin (Czech Republic) – a critical review. in *Cephalopods – Present and Past* (ed. Summerberger, H., Histon, K. & Daurer, A.) **57**, 359–369 (Abh. Geol. B.-A., 2002).

Košťák, M., Jagt, J. W. M., Speijer, R. P., Stassen, P. & Steurbaut, E. New Paleocene sepiid coleoids (Cephalopoda) from Egypt: evolutionary significance and origin of the sepiid "rostrum." *PLOS One* **8**, e81180 (2013).

Kruta, I., Bardin, J., Smith, C. P. A., Tafforeau, P. & Landman, N. H. Enigmatic hook-like structures in Cretaceous ammonites (Scaphitidae). *Palaeontology* **63**, 301–312, (2019).

Kruta, I. *et al*. *Proteroctopus ribeti* in coleoid evolution. *Palaeontology* **59**, 767–773 (2016).

Kulicki, C. & Szaniawski, K. Cephalopod arm hooks from the Jurassic of Poland. *Acta Palaeontol. Pol.* **17**, (1972).

Lindgren, A. R., Giribet, G. & Nishiguchi, M. K. A combined approach to the phylogeny of Cephalopoda (Mollusca). *Cladistics* **20**, 454–486 (2004).

Mapes, R. H. & Doguzhaeva, L. A. New Pennsylvanian coleoids (Cephalopoda) from Nebraska and Iowa, USA. *J. Paleontol.* **92**, 146–156 (2018).

Münster, G. & Graf Z. U. Die schalenlosen cephalopoden im unteren Jura, den Lias-Schiefern von Franken und Schwaben. *Beiträge zur Petrefaktenkunde* **6**, 57-77 (1843).

Naef, A. Das system der dibranchiaten cephalopoden und die mediterranen arten derselben. *Mitteilungen aus der zoologischen Station zu Neapel* **22**, 527-542 (1921).

Neige, P., Lapierre, H. & Merle, D. New Eocene coleoid (Cephalopoda) diversity from statolith remains: taxonomic assignation, fossil record analysis, and new data for calibrating molecular phylogenies. *PLOS One* **11**, e0154062 (2016).

Oudot, M. *et al*. The shell matrix and microstructure of the Ram's Horn squid: Molecular and structural characterization. *J. Struct. Biol.* **211**, 107507 (2020).

Reid, A. L. & Strugnell, J. M. A new pygmy squid, *Idiosepius hallami* n. sp. (Cephalopoda: Idiosepiidae) from eastern Australia and elevation of the southern endemic "notoides" clade to a new genus, *Xipholeptos* n. gen. *Zootaxa* **4369**, 451–486 (2018).

Riccardi, A. C. First teuthid cephalopod from the Lower Jurassic of South America (Neuquén Basin, Argentina). *Geol. Acta* **3**, 179–184 (2005).

Starobogatov, Y. I. Sistema golovonogikh molliuskov. in *Sistematika i Ekologiia Golovonogikh Molliuskov* (ed. Starobogatov, Y. I. & Nesis, K. N.). 4–7. (Zoological Institute of the USSR Academy of Sciences, 1983).

Sutton, M., Perales-Raya, C. & Gilbert, I. A phylogeny of fossil and living neocoleoid cephalopods. *Cladistics* **32**, 297–307 (2016).

Voltz, P. L. Notice sur les rapports des bélemnites avec d'autres coquilles internes de céphalopodes. *L'Institute, journal générale des sociétés et travaux scientifique de la France, 1ère section* **147**, 148-149 (1836).

von Boletzky, S. Evolutionary aspects of development, life style, and reproductive mode in incirrate octopods (Mollusca, Cephalopoda). *Rev. suisse Zool.* **99**, 755–770 (1992).

Yancey, T. E. & Garvie, C. L. Redescription of Anomalosaepia (Cephalopoda: Coleoida): a sepioid with a bimineralic calcite and aragonite skeleton. *J. Paleontol.* **85**, 904–915 (2011).

Young, R. E. & M. Vecchione. Analysis of morphology to determine primary sister-taxon relationships within coleoid cephalopods. *Am. Malacol. Bull.* **12**, 91–112 (1996).
