# Supplementary material for: Fossil coleoid cephalopod from the Mississippian Bear Gulch Lagerstätte sheds light on early vampyropod evolution
Source: Nat Commun. 2022 Mar 8;13:1107. doi: 10.1038/s41467-022-28333-5 (PMC8904582; doi:10.1038/s41467-022-28333-5)
Supplement: Supplementary file 2 — Description of Additional Supplementary Files [file 41467_2022_28333_MOESM2_ESM.pdf]

Description of Additional Supplementary Files

File Name: Supplementary Data 1

Description: NEXUS file for the phylogenetic analysis, including character-taxon matrix, tip-dates, and MrBayes settings.
