# Supplementary material for: Fossil coleoid cephalopod from the Mississippian Bear Gulch Lagerstätte sheds light on early vampyropod evolution
Source: Nat Commun. 2022 Mar 8;13:1107. doi: 10.1038/s41467-022-28333-5 (PMC8904582; doi:10.1038/s41467-022-28333-5)
Supplement: Supplementary file 4 — Reporting Summary [file 41467_2022_28333_MOESM4_ESM.pdf]

nature portfolio

Corresponding author(s): Christopher D. Whalen

Last updated by author(s): Dec 24, 2021

Reporting Summary

Nature Portfolio wishes to improve the reproducibility of the work that we publish. This form provides structure for consistency and transparency in reporting. For further information on Nature Portfolio policies, see our Editorial Policies and the Editorial Policy Checklist.

Statistics

For all statistical analyses, confirm that the following items are present in the figure legend, table legend, main text, or Methods section.

n/a	Confirmed	
☐	☒	The exact sample size (n) for each experimental group/condition, given as a discrete number and unit of measurement
☐	☒	A statement on whether measurements were taken from distinct samples or whether the same sample was measured repeatedly
☐	☒	The statistical test(s) used AND whether they are one- or two-sided<br>*Only common tests should be described solely by name; describe more complex techniques in the Methods section.*
☒	☐	A description of all covariates tested
☐	☒	A description of any assumptions or corrections, such as tests of normality and adjustment for multiple comparisons
☐	☒	A full description of the statistical parameters including central tendency (e.g. means) or other basic estimates (e.g. regression coefficient) AND variation (e.g. standard deviation) or associated estimates of uncertainty (e.g. confidence intervals)
☒	☐	For null hypothesis testing, the test statistic (e.g. F, t, r) with confidence intervals, effect sizes, degrees of freedom and P value noted<br>*Give P values as exact values whenever suitable.*
☐	☒	For Bayesian analysis, information on the choice of priors and Markov chain Monte Carlo settings
☒	☐	For hierarchical and complex designs, identification of the appropriate level for tests and full reporting of outcomes
☒	☐	Estimates of effect sizes (e.g. Cohen's d, Pearson's r), indicating how they were calculated

Our web collection on statistics for biologists contains articles on many of the points above.

Software and code

Policy information about availability of computer code

Data collection	n/a
Data analysis	MrBayes 3.2.7

For manuscripts utilizing custom algorithms or software that are central to the research but not yet described in published literature, software must be made available to editors and reviewers. We strongly encourage code deposition in a community repository (e.g. GitHub). See the Nature Portfolio guidelines for submitting code & software for further information.

Data

Policy information about availability of data

All manuscripts must include a data availability statement. This statement should provide the following information, where applicable:

- Accession codes, unique identifiers, or web links for publicly available datasets
- A description of any restrictions on data availability
- For clinical datasets or third party data, please ensure that the statement adheres to our policy

All data generated or analysed during this study are included in this published article (and its supplementary information files).

Field-specific reporting

Please select the one below that is the best fit for your research. If you are not sure, read the appropriate sections before making your selection.

☐ Life sciences ☐ Behavioural & social sciences ☒ Ecological, evolutionary & environmental sciences

For a reference copy of the document with all sections, see nature.com/documents/nr-reporting-summary-flat.pdf

Ecological, evolutionary & environmental sciences study design

All studies must disclose on these points even when the disclosure is negative.

Study description	We describe an exceptionally well-preserved fossil coleoid cephalopod, Syllipsimopodi bideni gen. et sp. nov., and place it in a phylogenetic context using the Bayesian fossilized birth-death process.
Research sample	We added Gordon icon us, Idahoteuthis, Acanthoteuthis, and Syllipsimopodi ge. nov. to the taxa sampled in Sutton et al. (2016) and Kruta et al. (2016).
Sampling strategy	We added Syllipsimopodi gen. nov. (the focus of the study), the co-occuring Gordoniconus, the enigmatic Idahoteuthis, and the well-preserved belemnoid Acanthoteuthis to the cited matrices.
Data collection	Morphological character data was collected by Christopher D. Whalen based on primary observations and cited literature, as documented in the Supplementary Information.
Timing and spatial scale	n/a
Data exclusions	Pohlsepia was excluded because it is almost certainly not a cephalopod, as described in the text. Jeletkya was excluded because it is very poorly understood and largely irrelevant, as discussed in the text. Marekites and Eoteuthoides were excluded because they are poorly preserved fossils, as mentioned in the text.
Reproducibility	In addition to providing the NEXUS and TRE files, all characters and taxa are discussed in detail in the text and associated Supplementary Information.
Randomization	n/a
Blinding	n/a

Did the study involve field work? ☐ Yes ☒ No

Reporting for specific materials, systems and methods

We require information from authors about some types of materials, experimental systems and methods used in many studies. Here, indicate whether each material, system or method listed is relevant to your study. If you are not sure if a list item applies to your research, read the appropriate section before selecting a response.

Materials & experimental systems

n/a	Involved in the study	
☒	☐	Antibodies
☒	☐	Eukaryotic cell lines
☐	☒	Palaeontology and archaeology
☒	☐	Animals and other organisms
☒	☐	Human research participants
☒	☐	Clinical data
☒	☐	Dual use research of concern

Methods

n/a	Involved in the study	
☒	☐	ChIP-seq
☒	☐	Flow cytometry
☒	☐	MRI-based neuroimaging

Palaeontology and Archaeology

Specimen provenance	The material is from the Bear Gulch Limestone, Heath Formation, Big Snowy Group in Fergus County, Montana, USA. The authors did not collect any new material. All analysed fossils are museum specimens.
Specimen deposition	Royal Ontario Museum, Toronto, Ontario, Canada. ROMIP 64897
Dating methods	No new dates are provided - references for stratigraphic dates are included in the text and the associated Supplementary Information

☐ Tick this box to confirm that the raw and calibrated dates are available in the paper or in Supplementary Information.

Ethics oversight	No ethical approval or guidance was required because this study only analysed invertebrate fossils in museum collections; no new material was collected. This study does not include any archaeological remains.

Note that full information on the approval of the study protocol must also be provided in the manuscript.
